# Real-world safety assessment of Ixekizumab based on the FDA Adverse Event Reporting System (FAERS)

**DOI:** 10.1371/journal.pone.0323973

**Published:** 2025-05-23

**Authors:** Yuzhe Cheng, Jingyi Ma, Jun Niu

**Affiliations:** 1 Department of Dermatology, General Hospital of Northern Theater Command, Shenyang, China; 2 Graduate School, China Medical University, Shenyang, China; Northwestern University Feinberg School of Medicine Galter Health Sciences Library, UNITED STATES OF AMERICA

## Abstract

**Background:**

Ixekizumab, a monoclonal antibody targeting IL-17A, is approved for psoriasis (PsO) and psoriatic arthritis (PsA). While clinical trials demonstrate its efficacy, real-world safety insights remain critical due to limitations in detecting rare or delayed adverse events (AEs).

**Methods:**

This study analyzed 28,889 ixekizumab-associated AE reports from the first quarter of 2016 to the third quarter of 2024 in the FDA Adverse Event Reporting System (FAERS) using disproportionality methods (ROR, PRR, MGPS, BCPNN) and Weibull distribution modeling. Subgroup and sensitivity analyses were conducted to evaluate demographic variations and confounding factors.

**Results:**

Common AEs included injection site reaction, fungal infections, and upper respiratory infections. Novel signals included myocardial infarction, herpes zoster, and inflammatory bowel disease. Subgroup analyses revealed male-predominant cardiac risks and age-dependent patterns (pediatric injection reactions vs elderly herpes zoster). Median time-to-onset was 56 days (IQR:12–205), with early risk escalation (Weibull β = 0.60).

**Conclusions:**

This FAERS analysis confirms ixekizumab’s established safety profile while identifying critical demographic-specific and delayed-onset signals. Continuous pharmacovigilance is warranted to optimize risk management, particularly for cardiovascular monitoring in high-risk males and antiviral prophylaxis in elderly patients.

## 1. Introduction

Psoriasis (PsO) and psoriatic arthritis (PsA) are chronic, immune-mediated inflammatory conditions that impose significant physical, psychological, and socioeconomic burdens on patients [1]. PsO affects approximately 2–3% of the global population, with up to 30% of these patients developing PsA, a debilitating musculoskeletal disorder characterized by joint damage and functional impairment [2]. Current therapeutic strategies include biologics targeting key inflammatory pathways, such as tumor necrosis factor (TNF), interleukin (IL)-12/23, IL-17, and IL-23 [3]. Four IL-17 inhibitors (secukinumab, brodalumab, ixekizumab, bimekizumab) and three IL-23 inhibitors (guselkumab, tildrakizumab, risankizumab) are currently approved by the European Medicines Agency and US Food and Drug Administration. Among these, ixekizumab, a monoclonal antibody targeting IL-17A, has demonstrated robust efficacy in clinical trials for both PsO and PsA, with rapid and sustained improvements in skin and joint symptoms [4,5].

These drugs showed very good results in randomised clinical trials [6,7]. However, this does not necessarily reflect their effectiveness in daily practice. While randomized controlled trials (RCTs) provide critical evidence for drug efficacy and safety, real-world evidence (RWE) is increasingly recognized as essential for understanding treatment outcomes in broader, more heterogeneous patient populations. RWE complements RCTs by capturing data from clinical practice, including long-term safety, treatment persistence, and patient-reported outcomes. Post-marketing surveillance systems, such as the FDA Adverse Event Reporting System (FAERS), play a pivotal role in identifying rare or delayed adverse events (AEs) that may not emerge during controlled trials. The FAERS database comprises an extensive repository of over 28 million reports on AEs, contributed by healthcare providers, patients, and drug manufacturers, providing a rich source of data on drug safety signals [8]. Despite ixekizumab’s established safety profile in RCTs, continuous pharmacovigilance is necessary to detect potential signals of harm in real-world settings, particularly for newer biologics with evolving therapeutic landscapes.

This study leverages FAERS data to conduct a comprehensive safety assessment of ixekizumab, employing disproportionality analyses to identify AE signals, characterize their temporal patterns, and explore demographic variations. By integrating these findings with existing RWE on ixekizumab’s clinical effectiveness and persistence, this analysis aims to refine risk-benefit evaluations and inform clinical decision-making for PsO and PsA management.

## 2. Materials and Methods

### 2.1. Data source

**Fig 1:**
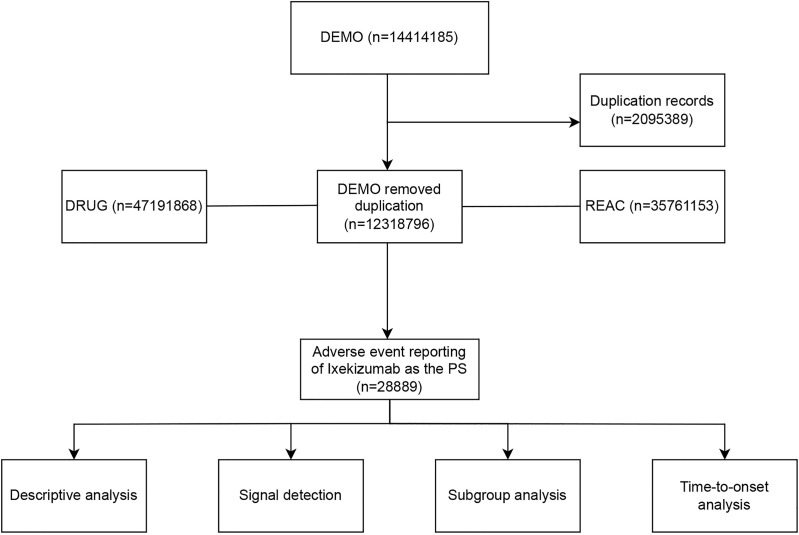
Flowchart demonstrating the adverse event analysis process for Ixekizumab using the FDA Adverse Event Reporting System database.

Fig 1 presents a comprehensive flowchart illustrating the study design. This research utilized data derived from the publicly available FAERS database, which is based on voluntary submissions from consumers, physicians, and pharmacists. Our analysis encompassed all AEs reports where Ixekizumab was identified as the primary suspected medication, spanning from the first quarter of 2016 to the third quarter of 2024. The data preprocessing involved eliminating duplicate records and harmonizing AE terminology in line with FDA-recommended standards. For reports with identical case identifiers (CASEIDs), we selected those with the latest FDA receipt date (FDA_DT). When both CASEID and FDA_DT were identical, the report with the highest PRIMARYID (a distinct identifier for each report) was chosen. AE terms were normalized utilizing the MedDRA dictionary (version 26.1), thus bolstering the accuracy of the ensuing statistical analyses.

### 2.2. Statistical analysis

We employed four disproportionality analysis methods to detect signals of potential adverse reactions link to ixekizumab: reporting odds ratio (ROR) [9], proportional reporting ratio (PRR) [10], multi-item gamma Poisson shrinker (MGPS) [11], and Bayesian confidence propagation neural network (BCPNN) [12]. These methods are commonly employed in the analysis of the FAERS database. The onset time of AEs linked to ixekizumab was determined as the interval between the occurrence of the event (documented in the DEMO file) and the commencement of ixekizumab treatment (recorded in the THER file). An event was deemed a potential adverse reaction if it satisfied the positivity criterion for at least one of the analysis methods. S1 Table presents detailed 2x2 contingency tables for reference purposes. The formulas and thresholds utilized in the disproportionality analyses are detailed in S2 Table. To enhance the accuracy of our results, we also conducted subgroup and sensitivity analyses. To model the temporal fluctuations in the incidence of AEs, we applied the Weibull distribution. All analyses were executed using R software (version 4.3.2).

## 3. Result

### 3.1. Descriptive analysis

After data deduplication, the study analyzed 28,889 AE reports where ixekizumab was the primary suspected drug from the first quarter of 2016 to the third quarter of 2024 in the FAERS database. Females accounted for 55.6% of cases, while males represented 36.0%. The majority of reports (41.6%) involved individuals aged 18–65 years, with only 0.2% of cases occurring in patients over 85 years. Non-healthcare professionals submitted 54.3% of reports, and 93.3% originated from the United States. Reporting frequency increased over time, peaking in 2022 (15.7%). Detailed demographic and reporting characteristics are summarized in Table 1.

**Table 1:** Clinical characteristics of Ixekizumab adverse event reports from the FAERS database (Q1 2016 – Q3 2024).

Characteristics	Case numbers	Case proportion (%)
Number of events	28889	
**Gender**		
Male	10400	36.0
Female	16050	55.6
Miss	2439	8.4
**Age**		
<18	255	0.9
18–65	12004	41.6
65–85	2456	8.5
>85	66	0.2
Miss	14108	48.8
**Top 5 Reported Countries**		
United States	26952	93.3
Germany	231	0.8
France	208	0.7
Japan	181	0.6
Canada	129	0.4
**Reporter**		
Healthcare professional	9927	34.4
Non-healthcare professional	15674	54.3
Miss	3288	11.4
**Reporting year**		
2016	360	1.2
2017	2020	7.0
2018	3583	12.4
2019	4487	15.5
2020	3138	10.9
2021	3598	12.5
2022	4529	15.7
2023	4036	14.0
2024	3138	10.9

### 3.2. Classification of AEs by system organ class (SOC) and preferred term (PT) level

**Fig 2:**
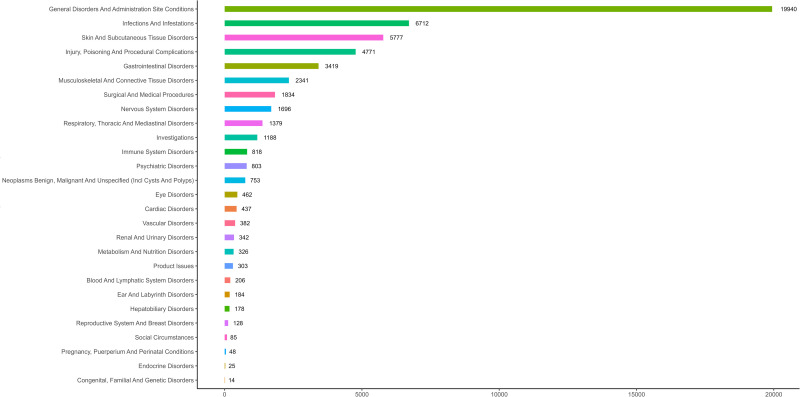
Numbers of adverse events by system organ class for Ixekizumab.

Ixekizumab-associated AEs spanned 27 SOCs, with statistically significant signals identified in infections and infestations, general disorders and administration site conditions, immune system disorders, skin and subcutaneous tissue disorders, and surgical and medical procedures (Fig 2 and Table 2). At the PT level, the top 50 AEs included injection site reactions (pain, erythema, swelling), infections (fungal infection, upper respiratory tract infection), hypersensitivity, and inflammatory bowel disease. Notably, label-uncharacterized signals such as rash, arthralgia, pruritus, arthritis, herpes zoster, swelling, myocardial infarction, stress and visual impairment were detected. S3 Table lists all PT-level AEs meeting signal thresholds, while Table 3 highlights the most frequent events.

**Table 2:** Signal strength of Ixekizumab AEs across System Organ Classes (SOC) in the FAERS database.

System Organ Class (SOC)	Case numbers	ROR(95%CI)	PRR(^2^)	EBGM(EBGM05)	IC(IC025)
Infections and infestations^*^	6712	2.42 (2.36 − 2.48)	2.24 (4882.58)	2.24 (2.19)	1.16 (1.13)
General disorders and administration site conditions^*^	19940	2.66 (2.61 − 2.7)	2.05 (13022.97)	2.05 (2.02)	1.03 (1.01)
Investigations	1188	0.36 (0.34 − 0.38)	0.38 (1307.97)	0.38 (0.36)	-1.41 (-1.5)
Respiratory, thoracic and mediastinal disorders	1379	0.53 (0.51 − 0.56)	0.55 (543.34)	0.55 (0.52)	-0.87 (-0.95)
Skin and subcutaneous tissue disorders^*^	5777	1.95 (1.89 − 2)	1.85 (2367.2)	1.84 (1.8)	0.88 (0.84)
Gastrointestinal disorders	3419	0.74 (0.72 − 0.77)	0.76 (288.82)	0.76 (0.74)	-0.4 (-0.45)
Immune system disorders^*^	818	1.23 (1.15 − 1.32)	1.23 (34.21)	1.22 (1.16)	0.29 (0.19)
Blood and lymphatic system disorders	206	0.22 (0.19 − 0.26)	0.23 (554.33)	0.23 (0.2)	-2.14 (-2.34)
Nervous system disorders	1696	0.38 (0.36 − 0.4)	0.4 (1673.1)	0.4 (0.38)	-1.33 (-1.4)
Musculoskeletal and connective tissue disorders	2341	0.83 (0.79 − 0.86)	0.83 (81.44)	0.83 (0.81)	-0.26 (-0.32)
Injury, poisoning and procedural complications	4771	0.75 (0.73 − 0.77)	0.77 (371.3)	0.77 (0.75)	-0.38 (-0.42)
Psychiatric disorders	803	0.26 (0.25 − 0.28)	0.27 (1623.52)	0.28 (0.26)	-1.86 (-1.96)
Social circumstances	85	0.34 (0.28 − 0.43)	0.35 (105.63)	0.35 (0.29)	-1.53 (-1.84)
Eye disorders	462	0.42 (0.38 − 0.46)	0.43 (363.42)	0.43 (0.4)	-1.23 (-1.36)
Hepatobiliary disorders	178	0.39 (0.34 − 0.45)	0.39 (169.36)	0.39 (0.35)	-1.35 (-1.57)
Metabolism and nutrition disorders	326	0.29 (0.26 − 0.32)	0.29 (562.35)	0.29 (0.27)	-1.76 (-1.92)
Cardiac disorders	437	0.38 (0.35 − 0.42)	0.39 (430.78)	0.39 (0.36)	-1.36 (-1.5)
Vascular disorders	382	0.36 (0.32 − 0.4)	0.36 (434.77)	0.36 (0.33)	-1.46 (-1.61)
Product issues	303	0.3 (0.27 − 0.34)	0.3 (489.36)	0.31 (0.28)	-1.71 (-1.88)
Surgical and medical procedures^*^	1834	2.39 (2.28 − 2.5)	2.34 (1424.74)	2.34 (2.25)	1.22 (1.16)
Renal and urinary disorders	342	0.31 (0.28 − 0.35)	0.32 (510.66)	0.32 (0.29)	-1.65 (-1.81)
Reproductive system and breast disorders	128	0.33 (0.28 − 0.39)	0.33 (175.04)	0.33 (0.29)	-1.6 (-1.85)
Neoplasms benign, malignant and unspecified (incl cysts and polyps)	753	0.44 (0.41 − 0.47)	0.45 (531.47)	0.45 (0.42)	-1.16 (-1.27)
Ear and labyrinth disorders	184	0.77 (0.67 − 0.89)	0.77 (12.6)	0.77 (0.68)	-0.38 (-0.59)
Endocrine disorders	25	0.17 (0.12 − 0.26)	0.17 (98.06)	0.17 (0.13)	-2.52 (-3.09)
Congenital, familial and genetic disorders	14	0.09 (0.06 − 0.16)	0.09 (122.33)	0.09 (0.06)	-3.41 (-4.15)
Pregnancy, puerperium and perinatal conditions	48	0.23 (0.17 − 0.3)	0.23 (126.78)	0.23 (0.18)	-2.14 (-2.55)

Abbreviation: Asterisks (

*) indicate statistically significant signals in algorithm; ROR, reporting odds ratio; PRR, proportional reporting ratio; EBGM, empirical Bayesian geometric mean; EBGM05, the lower limit of the 95% CI of EBGM; IC, information component; IC025, the lower limit of the 95% CI of the IC; CI, confidence interval; AEs, adverse events.

**Table 3:** Top 50 frequency of adverse events at the PT level for Ixekizumab.

PT	Case numbers	ROR(95%CI)	PRR(^2^)	EBGM(EBGM05)	IC(IC025)
Injection site pain^*^	3,581	16.05 (15.51 − 16.61)	15.06 (46163.17)	14.75 (14.33)	3.88 (3.83)
Psoriasis^*^	2,205	18.07 (17.31 − 18.87)	17.38 (33236.44)	16.96 (16.35)	4.08 (4.02)
Drug ineffective^*^	2,054	1.6 (1.54 − 1.68)	1.58 (449.53)	1.58 (1.52)	0.66 (0.6)
Injection site erythema^*^	2,014	26.19 (25.03 − 27.41)	25.26 (45249.64)	24.36 (23.45)	4.61 (4.54)
Injection site swelling^*^	1,519	28.29 (26.86 − 29.8)	27.53 (37306.84)	26.46 (25.33)	4.73 (4.65)
Injection site reaction^*^	1,459	30.4 (28.83 − 32.06)	29.62 (38626.91)	28.37 (27.14)	4.83 (4.75)
Covid-19^*^	775	3.25 (3.03 − 3.49)	3.22 (1186.58)	3.21 (3.03)	1.68 (1.58)
Incorrect dose administered^*^	770	3.81 (3.54 − 4.09)	3.77 (1561.42)	3.75 (3.53)	1.91 (1.8)
Product dose omission issue^*^	712	2.39 (2.22 − 2.57)	2.37 (565.01)	2.37 (2.22)	1.24 (1.13)
Therapy interrupted^*^	711	9.91 (9.2 − 10.68)	9.8 (5539.53)	9.67 (9.08)	3.27 (3.16)
Injection site pruritus^*^	695	14.49 (13.44 − 15.63)	14.32 (8434.19)	14.03 (13.17)	3.81 (3.7)
Rash^*^	611	1.56 (1.44 − 1.69)	1.56 (122.11)	1.56 (1.45)	0.64 (0.52)
Injection site urticaria^*^	606	32.49 (29.93 − 35.27)	32.14 (17432.15)	30.68 (28.64)	4.94 (4.82)
Diarrhoea	571	0.95 (0.87 − 1.03)	0.95 (1.76)	0.95 (0.88)	-0.08 (-0.2)
Injection site mass^*^	549	15.18 (13.94 − 16.52)	15.03 (7034.93)	14.72 (13.71)	3.88 (3.75)
Arthralgia^*^	525	1.39 (1.28 − 1.51)	1.39 (56.71)	1.39 (1.29)	0.47 (0.34)
Pruritus^*^	523	1.59 (1.46 − 1.74)	1.59 (113.97)	1.59 (1.48)	0.67 (0.54)
Injection site haemorrhage^*^	512	7.79 (7.13 − 8.5)	7.72 (2964.55)	7.64 (7.1)	2.93 (2.81)
Nasopharyngitis^*^	475	2.81 (2.56 − 3.07)	2.79 (545.06)	2.78 (2.58)	1.48 (1.34)
Injection site rash^*^	468	20.43 (18.63 − 22.41)	20.27 (8317.37)	19.69 (18.22)	4.3 (4.16)
Inappropriate schedule of product administration^*^	467	2.28 (2.08 − 2.49)	2.26 (329.84)	2.26 (2.09)	1.18 (1.04)
Pain	462	0.79 (0.72 − 0.86)	0.79 (25.87)	0.79 (0.73)	-0.34 (-0.47)
Injection site warmth^*^	451	38.5 (35 − 42.35)	38.19 (15434)	36.13 (33.36)	5.18 (5.04)
Nausea	437	0.65 (0.59 − 0.71)	0.65 (81.22)	0.65 (0.6)	-0.61 (-0.75)
Urticaria^*^	436	3.12 (2.84 − 3.43)	3.1 (618.94)	3.09 (2.86)	1.63 (1.49)
Injection site bruising^*^	415	6.66 (6.05 − 7.34)	6.62 (1962.04)	6.56 (6.05)	2.71 (2.57)
Sinusitis^*^	397	4.34 (3.93 − 4.79)	4.31 (1005.2)	4.29 (3.95)	2.1 (1.96)
Malaise	383	0.97 (0.87 − 1.07)	0.97 (0.48)	0.97 (0.89)	-0.05 (-0.2)
Infection^*^	382	2.88 (2.61 − 3.19)	2.87 (465.06)	2.86 (2.63)	1.52 (1.37)
Therapy cessation^*^	378	6.19 (5.59 − 6.85)	6.15 (1618.05)	6.11 (5.61)	2.61 (2.46)
Fatigue	376	0.51 (0.46 − 0.56)	0.51 (176.47)	0.51 (0.47)	-0.96 (-1.11)
Headache	340	0.61 (0.55 − 0.68)	0.62 (82.48)	0.62 (0.56)	-0.7 (-0.86)
Hypersensitivity^*^	338	1.99 (1.78 − 2.21)	1.98 (164.06)	1.98 (1.81)	0.98 (0.83)
Psoriatic arthropathy^*^	328	8.14 (7.3 − 9.08)	8.1 (2017.34)	8.01 (7.31)	3 (2.84)
Urinary tract infection^*^	325	2.11 (1.89 − 2.35)	2.1 (187.59)	2.1 (1.91)	1.07 (0.91)
Illness^*^	311	2.91 (2.6 − 3.25)	2.9 (385.31)	2.89 (2.63)	1.53 (1.37)
Influenza^*^	293	2.81 (2.51 − 3.16)	2.81 (339.55)	2.8 (2.54)	1.48 (1.32)
Pyrexia	292	1 (0.89 − 1.12)	1 (0)	1 (0.91)	0 (-0.17)
Death	277	0.34 (0.31 − 0.39)	0.35 (345.66)	0.35 (0.31)	-1.53 (-1.7)
Condition aggravated	270	0.9 (0.8 − 1.02)	0.9 (2.76)	0.9 (0.82)	-0.15 (-0.32)
Pneumonia	264	0.91 (0.81 − 1.03)	0.91 (2.11)	0.91 (0.83)	-0.13 (-0.31)
Cellulitis^*^	261	6.08 (5.38 − 6.87)	6.06 (1093.16)	6.01 (5.43)	2.59 (2.41)
Underdose^*^	241	3.16 (2.78 − 3.58)	3.15 (352.03)	3.14 (2.82)	1.65 (1.46)
Off label use	234	0.24 (0.21 − 0.27)	0.24 (568.35)	0.24 (0.22)	-2.05 (-2.24)
Pain in extremity	215	0.85 (0.75 − 0.98)	0.85 (5.35)	0.85 (0.76)	-0.23 (-0.42)
Erythema	215	1.1 (0.96 − 1.25)	1.1 (1.8)	1.1 (0.98)	0.13 (-0.07)
Ear infection^*^	213	8.66 (7.56 − 9.91)	8.63 (1417.93)	8.53 (7.61)	3.09 (2.89)
Alopecia	212	1.02 (0.89 − 1.17)	1.02 (0.12)	1.02 (0.91)	0.03 (-0.16)
Accidental underdose^*^	196	13.22 (11.48 − 15.23)	13.18 (2162.69)	12.94 (11.49)	3.69 (3.49)
Cough	195	0.75 (0.65 − 0.86)	0.75 (16.1)	0.75 (0.67)	-0.41 (-0.62)

Abbreviation: Asterisks (

*) indicate statistically significant signals in algorithm; ROR, reporting odds ratio; PRR, proportional reporting ratio; EBGM, empirical Bayesian geometric mean; EBGM05, the lower limit of the 95% CI of EBGM; IC, information component; IC025, the lower limit of the 95% CI of the IC; CI, confidence interval; PT, preferred term.

### 3.3. Subgroup analysis

Subgroup analysis revealed, In addition to more adverse cardiac effects in men(cardiac failure congestive, myocardial infarction, ejection fraction decreased), minimal gender-based differences in AE profiles (S4 and S5 Tables). In pediatric patients (<18 years), common signals included injection site reactions, fatigue, diarrhoea, nasopharyngitis, pharyngitis streptococcal, covid-19, urticaria, alopecia, eye infection, cough and oropharyngeal pain. Adults (18–65 years) frequently reported injection site reactions, nasopharyngitis, rash, sinusitis, arthralgia, infection, malaise, pruritus, urticaria, influenza and urinary tract infection. Older adults (>65 years) frequently reported injection site reactions, covid-19, urinary tract infection, pain, arthralgia, pruritus, rheumatoid arthritis, infection, drug hypersensitivity, urticaria, herpes zoster and skin exfoliation. Age-stratified results are detailed in S6–S8 Tables.

### 3.4. Time to onset and Weibull distribution analysis of AEs

**Fig 3:**
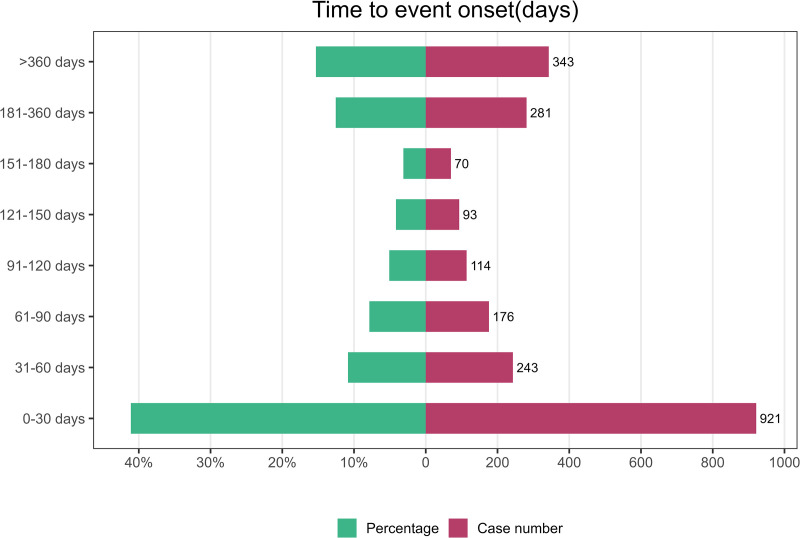
Time to onset of adverse events induced by Ixekizumab.

**Fig 4:**
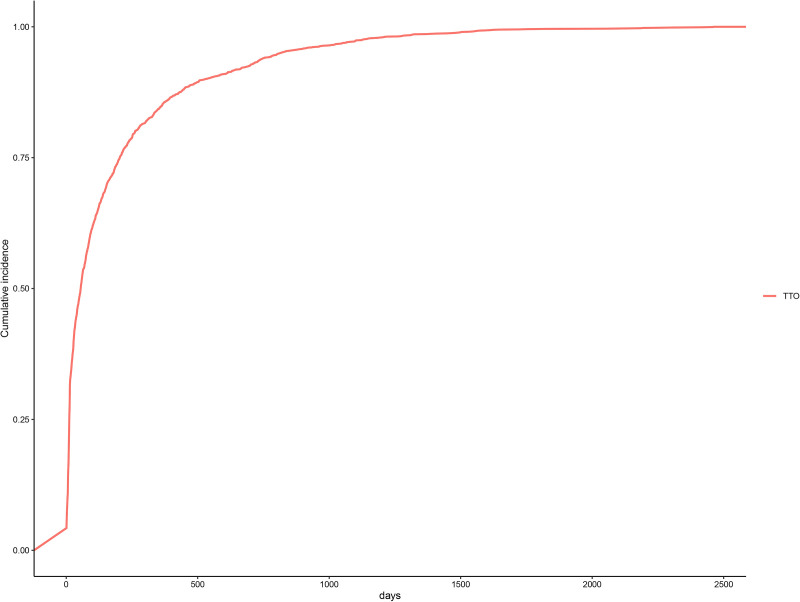
Cumulative incidence of adverse events related to Ixekizumab over time.

The median time-to-onset (TTO) of ixekizumab-associated AEs was 56 days (IQR: 12–205 days). Weibull distribution analysis confirmed an early failure pattern (shape parameter β = 0.60, 95% CI: 0.58–0.61), indicating higher risk shortly after treatment initiation. Fig 3 illustrates the temporal distribution of these events with clarity. Cumulative incidence curves and temporal distributions are shown in Fig 4 and Table 4.

**Table 4:** Time to onset of -associated adverse events and Weibull distribution analysis.

Drug	TTO(days)		Weibull distribution		
Ixekizumab	Case reports	Median(d)(IQR)	Scale parameter: α(95%CI)	Shape parameter: β(95%CI)	Type
	2241	56(12-205)	121.58 (112.63-130.52)	0.60 (0.58-0.61)	Early failure

Abbreviation: TTO,time to onset; CI, confidence interval; IQR, interquartile range.

### 3.5. Sensitivity analysis

After excluding cases involving concomitant therapies (e.g., secukinumab, adalimumab, methotrexate, etanercept, methotrexate, prednisone, ustekinumab, sulfasalazine, apremilast, infliximab, tofacitinib, hydroxychloroquine, adalimumab, leflunomide), 27204 AE reports remained. Consistent signals included, injection site reactions, infection, rash, diarrhoea, pruritus, arthralgia, nasopharyngitis, urticaria, hypersensitivity. Full results are provided in S9 Table.

## 4. Discussion

The present study analyzed 28,889 ixekizumab-associated AE reports from the FAERS database, employing disproportionality methods to identify safety signals and temporal trends. Our findings align with known ixekizumab safety profiles while uncovering novel insights into underreported AEs, demographic variations, and timing of onset. These results complement existing RWE on ixekizumab’s clinical efficacy and persistence, providing a holistic view of its real-world performance.

Previous studies underscore ixekizumab’s robust efficacy in PsO and PsA, with high treatment persistence rates (81.3% at 1 year in Swedish registries) and superior clinical outcomes compared to anti-TNF agents like adalimumab [13]. Real-world studies consistently report PASI75/90/100 responses exceeding 70%, 50%, and 38%, respectively, at 12–24 weeks, aligning with clinical trial data. Notably, biologic-naïve patients demonstrate enhanced responses, with PASI90 rates of 71.4% versus 43.8% in anti-IL-17A-experienced individuals. These findings validate ixekizumab’s position as a first-line biologic for moderate-to-severe PsO, particularly in patients without prior biologic exposure. Safety profiles from real-world studies largely mirror those observed in randomized controlled trials (RCTs). Injection site reactions and infections remain the most frequently reported AEs, consistent with ixekizumab’s IL-17A inhibition mechanism. However, the FAERS analysis expands this understanding by identifying novel signals, including myocardial infarction, herpes zoster, and inflammatory bowel disease, which were less prominent in RCTs. Geographic and ethnic variances warrant attention, as FAERS data predominantly reflect European populations (93.3% US-originated reports), whereas Asian clinical trials reveal distinct AE profiles. For instance, a two-year real-world study in China reported local injection reactions (31.8%), allergies (11.6%), and infections (6.1%) as the most common AEs, with no disease activation observed in patients with latent tuberculosis or hepatitis B/C [14]. Another study on Chinese patients with moderate-to-severe PsO found that most treatment-emergent AEs (TEAEs) were mild and moderate in severity, with no new safety concerns observed on retreatment [15].These discrepancies highlight the limitations of controlled trials in detecting rare or delayed AEs, emphasizing the critical role of post-marketing surveillance in pharmacovigilance.

The FAERS analysis of 28,889 ixekizumab-associated AE reports identified both expected and unexpected safety signals. Injection site reactions s (e.g., pain, erythema, swelling) and infections (e.g., upper respiratory tract infections, fungal infections) dominated the AE profile, corroborating RCT and real-world data. The predominance of injection site reactions—including pain (15.2%), erythema (9.8%), and swelling (7.4%)—aligns with phase III trials reporting injection site reactions frequencies of 12–17% [4,5]. Mechanistically, these reactions may arise from ixekizumab’s citrate-containing formulation, which induces transient local inflammation [16]. Recent advancements in drug delivery, such as the citrate-free (CF) ixekizumab formulation, have demonstrated reduced injection site reactions incidence (from 19.2% to 5.6% in switching studies), suggesting formulation optimization as a key strategy for improving tolerability [17,18]. Infection-related signals (e.g., fungal infections, upper respiratory tract infections) mirror IL-17A’s physiological role in mucosal immunity and antifungal defense [19,20]. Notably, 89% of these cases were superficial (oral/genital), managed with topical antifungals or observation, with only 0.1% leading to treatment discontinuation [21]. However, FAERS data identified higher-than-expected herpes zoster signals, likely reflecting IL-17A’s critical role in controlling varicella-zoster virus reactivation [22]. This contrasts with anti-TNF agents, which exhibit stronger associations with tuberculosis reactivation [22], highlighting pathway-specific infection risks.

The detection of myocardial infarction (MI) as a signal in elderly males requires nuanced interpretation. While disproportionality analyses suggest elevated reporting rates, this finding must be contextualized against the inherently higher baseline cardiovascular risk in elderly males, particularly those with moderate-to-severe PsO. PsO is independently associated with an increased risk of major adverse cardiovascular events (MACE) [23]. FAERS data lack denominator-based incidence rates and cannot fully adjust for confounding by disease severity or traditional risk factors (e.g., smoking, hypertension). Importantly, the elevated ROR does not imply causation. Elderly males with PsO represent a population already at heightened MI risk due to age, systemic inflammation, and comorbidities. Thus, the observed signal may partially reflect confounding by indication rather than a direct drug effect. Clinicians must weigh this signal against robust evidence demonstrating that systemic inflammation control with biologics reduces cardiovascular morbidity. For instance, a meta-analysis of 7 RCTs found no increased MACE risk with ixekizumab [22]. Furthermore, a safety analysis of 25 RCTs showed that the incidence of adjudicated MACE was low among adult patients with PsO, PsA, and axial spondyloarthritis (axSpA), and that these rates did not increase with greater exposure to ixekizumab [24]. Emerging evidence underscores that systemic inflammation reduction via biologics may attenuate PsO -associated cardiovascular mortality. A retrospective analysis from South Korea also found that in patients with PsO or PsA, biologic therapy was associated with a lower risk of new-onset and recurrent MACEs compared to phototherapy [25]. Critically, untreated PsO poses greater cardiovascular harm than theoretical risks from IL-17A inhibition. Elderly males with PsO face substantially higher MI risk than age-matched controls without PsO, making effective anti-inflammatory treatment imperative. New-onset inflammatory bowel disease cases challenge the traditional view of IL-17A as purely pro-inflammatory in gut mucosa. Paradoxically, IL-17A knockout mice exhibit exacerbated colitis, suggesting a protective role in epithelial repair [20]. This duality may explain why ixekizumab-triggered inflammatory bowel disease occurred predominantly in patients with preexisting subclinical inflammation (e.g., elevated fecal calprotectin). Signals for arthralgia, rash, and pruritus may reflect residual psoriatic inflammation or neuroimmune crosstalk. IL-17A directly activates sensory neurons expressing IL-17 receptors, and its blockade could dysregulate itch pathways [26]. Notably, ixekizumab demonstrated superior efficacy in psoriatic patients with comorbid depression (QIDS-SR16 improvement: Δ=−8.2 vs. −5.1 for placebo) [26], suggesting neuroimmunological benefits that partially offset these AE risks. In terms of infection risks, a study on systemic therapies for PsO revealed that cutaneous bacterial infections were frequently observed in patients receiving TNF-α and IL-17 inhibitor treatments [19]. Furthermore, age and use of IL-17 inhibitors were associated with elevated odds ratios for fungal infections. Our study reveals that systemic therapies may increase the risk of cutaneous viral infections. Therefore, dermatologists should exercise caution in this regard [19]. Unexpected signals such as rash, arthralgia, and pruritus were also detected. These may reflect residual inflammatory activity in refractory PsO/PsA or paradoxical reactions to IL-17A inhibition. For instance, IL-17A blockade in PsA could disrupt joint homeostasis, leading to transient arthralgia. Similarly, pruritus may arise from neuroimmune interactions altered by ixekizumab. While these AEs are rarely severe, their prevalence in real-world settings suggests a need for patient education and symptom management strategies.

Subgroup analyses from FAERS and the literature review highlight age- and sex-dependent AE patterns. Pediatric patients (<18 years) predominantly experienced injection site reactions and mild infections (e.g., nasopharyngitis, streptococcal pharyngitis), with no severe safety signals. This aligns with limited real-world pediatric data, which describe ixekizumab as well-tolerated in adolescents. However, long-term safety in this population remains uncertain, particularly regarding growth and developmental impacts. In adults aged 18–65, injection site reactions, infections, and autoimmune-like reactions (e.g., urticaria, drug hypersensitivity) were common. Real-world studies note that biologic-experienced patients face higher discontinuation rates due to secondary failure or AEs, suggesting that prior biologic exposure may amplify ixekizumab’s immunogenic potential. For example, a Spanish cohort reported 68.5% biologic-experienced patients, among whom 23% discontinued ixekizumab due to loss of efficacy or intolerance. This contrasts with biologic-naïve populations, where persistence exceeds 80% at 12 months. Older adults (>65 years) exhibited distinct risks, including herpes zoster, skin exfoliation, and cardiovascular events. Age-related immunosenescence likely contributes to viral reactivation, while comorbidities such as diabetes and hypertension may exacerbate cardiovascular risks. The FAERS analysis identified a median time-to-onset (TTO) of 56 days for AEs, but herpes zoster and malignancies often manifested later (>6 months), underscoring the importance of extended monitoring in elderly populations. Gender disparities were evident in cardiac AEs, with males reporting higher rates of myocardial infarction and congestive heart failure. Biological factors, such as androgen-driven immune responses, and sociobehavioral factors, including delayed healthcare-seeking in males, may underlie these differences. Tailored risk mitigation strategies, such as lipid profiling and blood pressure monitoring, could optimize outcomes in high-risk subgroups.

The FAERS analysis revealed a biphasic pattern in AE onset, with early-phase events (e.g., injection site reactions, hypersensitivity) peaking within 60 days and late-phase events (e.g., herpes zoster, malignancies) emerging after 6 months. The Weibull shape parameter (β = 0.60) confirmed this early failure pattern, suggesting heightened immune activation during treatment initiation. These findings align with real-world persistence data, where discontinuation rates are highest in the first 3–6 months due to AEs or inadequate response.

Proactive monitoring during the initial treatment phase is critical to mitigate early AEs. For instance, injection site reactions can often be managed with topical analgesics or rotation of injection sites, while hypersensitivity reactions may necessitate treatment interruption. Conversely, late-phase AEs like herpes zoster require long-term vigilance, particularly in older adults. For high-risk patients (e.g., age > 65, diabetes), antiviral prophylaxis (e.g., acyclovir) could be considered, balancing benefits against potential drug interactions. Early recognition of dermatomal pain or rash is critical to prevent postherpetic neuralgia. To address potential cardiovascular adverse reactions, clinicians should prioritize cardiovascular risk assessment, including lipid profiling and blood pressure monitoring, before initiating ixekizumab, especially in older males with metabolic comorbidities. Although causality remains unproven, it is advisable to monitor for symptoms like chest pain or dyspnea during treatment, and promptly refer to cardiology if these symptoms occur. The identification of inflammatory bowel disease (IBD) signals necessitates enhanced surveillance of gastrointestinal symptoms, particularly in patients with a history of subclinical inflammation. Baseline assessment of fecal calprotectin or other markers of gut inflammation may aid in identifying patients at elevated risk. Regular follow-up to detect early signs of gastrointestinal adverse events is advisable. Should patients experience diarrhea or abdominal pain, prompt endoscopic evaluation is warranted. Close collaboration between dermatologists and gastroenterologists is crucial to ensure optimal patient management and timely intervention.

While the FAERS data offer valuable real-world insights, they are not without limitations, including underreporting, incomplete clinical details, and the inability to establish causality. Firstly, the FAERS database relies on voluntary reporting, which may introduce biases and lead to underreporting. These include underreporting, where not all AEs are documented, potentially leading to an underestimation of true incidence rates. Additionally, reporting bias may result in over-representation of certain AEs due to heightened awareness or media attention. Approximately 54% of reports originated from non-healthcare professionals, which may affect data accuracy. Furthermore, the lack of comprehensive clinical information in the database hinders our ability to account for confounding factors. Confounding by concomitant therapies, particularly other biologics or immunosuppressants, may bias results despite sensitivity analyses. Additionally, overrepresentation of U.S. reports (93.3%) limits generalizability to global populations. Future research should prioritize prospective cohort studies to validate identified signals and elucidate mechanistic links between IL-17 inhibition and cardiac or neoplastic events. Comparative safety analyses against other IL-17 inhibitors (e.g., secukinumab) and IL-23 blockers could clarify class-specific risks. Future research should prioritize prospective cohort studies with extended follow-up periods to validate identified signals and elucidate mechanistic links between IL-17 inhibition and adverse events such as cardiovascular events and herpes zoster. Additionally, integrating FAERS data with electronic health records may improve AE attribution and enable risk stratification based on patient-specific factors. Research focusing on diverse geographic and ethnic populations is also needed to ensure the generalizability of findings. Finally, the development of risk prediction models using clinical and demographic factors could enhance the precision of safety monitoring and guide personalized treatment strategies.

## 5. Conclusion

This FAERS-based analysis reinforces ixekizumab’s established safety profile while uncovering novel signals that require clinical vigilance. The drug’s favorable benefit-risk profile in PsO and PsA is well-supported by high real-world persistence and efficacy rates documented in prior RWE studies. However, continued monitoring for infections, cardiovascular events, and unlabeled adverse events AEs, particularly in vulnerable patient subgroups, remains essential. Regulatory bodies should consider mandating long-term safety studies and integrating real-world data to enhance traditional pharmacovigilance systems. Guidance for healthcare providers should be updated to include proactive monitoring strategies for identified risks, such as regular cardiovascular assessments in elderly males and heightened surveillance for herpes zoster in older adults. Product labeling should be periodically revised to incorporate emerging safety data, ensuring that prescribing decisions are based on the most current evidence. Transparent communication of safety signals aids in informed decision-making and strengthens patient-provider discussions. These recommendations underscore the critical role of pharmacovigilance in optimizing therapeutic outcomes and ensuring patient safety in real-world practice. Future regulatory policies should leverage real-world evidence to refine risk-benefit evaluations and support the evolving understanding of biologic therapies like ixekizumab.

## Supporting information

S1 Table Two-by-two contingency table for disproportionality analyses. (DOCX)

S2 Table Four major algorithms used for signal detection. (DOCX)

S3 Table All adverse events of Ixekizumab meeting the positive signal threshold at the PT level from FAERS data. (DOCX)

S4 Table Top 50 most frequent positive signal adverse events of Ixekizumab at the preferred term (PT) level in males from FAERS data. (DOCX)

S5 Table Top 50 most frequent positive signal adverse events of Ixekizumab at the PT level in females from FAERS data. (DOCX)

S6 Table Top 50 most frequent positive signal adverse events of Ixekizumab at the PT level in patients aged under 18 from FAERS data. (DOCX)

S7 Table Top 50 most frequent positive signal adverse events of Ixekizumab at the PT level in patients aged 18–65 from FAERS data. (DOCX)

S8 Table Top 50 most frequent positive signal adverse events of Ixekizumab at the PT level in patients aged 65–85 from FAERS data. (DOCX)

S9 Table Top 50 most frequent positive signal adverse events of Ixekizumab excluding common medication co-usage at the PT level from FAERS data. (DOCX)
