# Supplementary material for: Real-world safety assessment of Ixekizumab based on the FDA Adverse Event Reporting System (FAERS)
Source: PLoS One. 2025 May 23;20(5):e0323973. doi: 10.1371/journal.pone.0323973 (PMC12101745; doi:10.1371/journal.pone.0323973)
Supplement: S1 Table — (DOCX) [file pone.0323973.s001.docx]

Supplementary Table 1:

Two-by-two contingency table for disproportionality analyses.

|  | Target AEs | Other AEs | Total |
| --- | --- | --- | --- |
| Ixekizumab | a | b | a+b |
| Other drugs | c | d | c+d |
| Total | a+c | b+d | a+b+c+d |

Abbreviation: AEs, adverse events; a, number of reports containing both the target drug and target adverse drug reaction; b, number of reports containing other adverse drug reaction of the target drug; c, number of reports containing the target adverse drug reaction of other drugs; d, number of reports containing other drugs and other adverse drug reactions.
