# Supplementary material for: Real-world safety assessment of Ixekizumab based on the FDA Adverse Event Reporting System (FAERS)
Source: PLoS One. 2025 May 23;20(5):e0323973. doi: 10.1371/journal.pone.0323973 (PMC12101745; doi:10.1371/journal.pone.0323973)
Supplement: S3 Table — (DOCX) [file pone.0323973.s003.docx]

Supplementary Table 3 :

All adverse events of Ixekizumab meeting the positive signal threshold at the PT level from FAERS data

| PT | Case numbers | ROR(95%CI) | PRR(χ^2^) | EBGM(EBGM05) | IC(IC025) |
| --- | --- | --- | --- | --- | --- |
| Injection site pain | 3,581 | 16.05 ( 15.51 - 16.61 ) | 15.06 ( 46163.17 ) | 14.75 ( 14.33 ) | 3.88 ( 3.83 ) |
| Psoriasis | 2,205 | 18.07 ( 17.31 - 18.87 ) | 17.38 ( 33236.44 ) | 16.96 ( 16.35 ) | 4.08 ( 4.02 ) |
| Drug ineffective | 2,054 | 1.6 ( 1.54 - 1.68 ) | 1.58 ( 449.53 ) | 1.58 ( 1.52 ) | 0.66 ( 0.6 ) |
| Injection site erythema | 2,014 | 26.19 ( 25.03 - 27.41 ) | 25.26 ( 45249.64 ) | 24.36 ( 23.45 ) | 4.61 ( 4.54 ) |
| Injection site swelling | 1,519 | 28.29 ( 26.86 - 29.8 ) | 27.53 ( 37306.84 ) | 26.46 ( 25.33 ) | 4.73 ( 4.65 ) |
| Injection site reaction | 1,459 | 30.4 ( 28.83 - 32.06 ) | 29.62 ( 38626.91 ) | 28.37 ( 27.14 ) | 4.83 ( 4.75 ) |
| Covid-19 | 775 | 3.25 ( 3.03 - 3.49 ) | 3.22 ( 1186.58 ) | 3.21 ( 3.03 ) | 1.68 ( 1.58 ) |
| Incorrect dose administered | 770 | 3.81 ( 3.54 - 4.09 ) | 3.77 ( 1561.42 ) | 3.75 ( 3.53 ) | 1.91 ( 1.8 ) |
| Product dose omission issue | 712 | 2.39 ( 2.22 - 2.57 ) | 2.37 ( 565.01 ) | 2.37 ( 2.22 ) | 1.24 ( 1.13 ) |
| Therapy interrupted | 711 | 9.91 ( 9.2 - 10.68 ) | 9.8 ( 5539.53 ) | 9.67 ( 9.08 ) | 3.27 ( 3.16 ) |
| Injection site pruritus | 695 | 14.49 ( 13.44 - 15.63 ) | 14.32 ( 8434.19 ) | 14.03 ( 13.17 ) | 3.81 ( 3.7 ) |
| Rash | 611 | 1.56 ( 1.44 - 1.69 ) | 1.56 ( 122.11 ) | 1.56 ( 1.45 ) | 0.64 ( 0.52 ) |
| Injection site urticaria | 606 | 32.49 ( 29.93 - 35.27 ) | 32.14 ( 17432.15 ) | 30.68 ( 28.64 ) | 4.94 ( 4.82 ) |
| Injection site mass | 549 | 15.18 ( 13.94 - 16.52 ) | 15.03 ( 7034.93 ) | 14.72 ( 13.71 ) | 3.88 ( 3.75 ) |
| Arthralgia | 525 | 1.39 ( 1.28 - 1.51 ) | 1.39 ( 56.71 ) | 1.39 ( 1.29 ) | 0.47 ( 0.34 ) |
| Pruritus | 523 | 1.59 ( 1.46 - 1.74 ) | 1.59 ( 113.97 ) | 1.59 ( 1.48 ) | 0.67 ( 0.54 ) |
| Injection site haemorrhage | 512 | 7.79 ( 7.13 - 8.5 ) | 7.72 ( 2964.55 ) | 7.64 ( 7.1 ) | 2.93 ( 2.81 ) |
| Nasopharyngitis | 475 | 2.81 ( 2.56 - 3.07 ) | 2.79 ( 545.06 ) | 2.78 ( 2.58 ) | 1.48 ( 1.34 ) |
| Injection site rash | 468 | 20.43 ( 18.63 - 22.41 ) | 20.27 ( 8317.37 ) | 19.69 ( 18.22 ) | 4.3 ( 4.16 ) |
| Inappropriate schedule of product administration | 467 | 2.28 ( 2.08 - 2.49 ) | 2.26 ( 329.84 ) | 2.26 ( 2.09 ) | 1.18 ( 1.04 ) |
| Injection site warmth | 451 | 38.5 ( 35 - 42.35 ) | 38.19 ( 15434 ) | 36.13 ( 33.36 ) | 5.18 ( 5.04 ) |
| Urticaria | 436 | 3.12 ( 2.84 - 3.43 ) | 3.1 ( 618.94 ) | 3.09 ( 2.86 ) | 1.63 ( 1.49 ) |
| Injection site bruising | 415 | 6.66 ( 6.05 - 7.34 ) | 6.62 ( 1962.04 ) | 6.56 ( 6.05 ) | 2.71 ( 2.57 ) |
| Sinusitis | 397 | 4.34 ( 3.93 - 4.79 ) | 4.31 ( 1005.2 ) | 4.29 ( 3.95 ) | 2.1 ( 1.96 ) |
| Infection | 382 | 2.88 ( 2.61 - 3.19 ) | 2.87 ( 465.06 ) | 2.86 ( 2.63 ) | 1.52 ( 1.37 ) |
| Therapy cessation | 378 | 6.19 ( 5.59 - 6.85 ) | 6.15 ( 1618.05 ) | 6.11 ( 5.61 ) | 2.61 ( 2.46 ) |
| Hypersensitivity | 338 | 1.99 ( 1.78 - 2.21 ) | 1.98 ( 164.06 ) | 1.98 ( 1.81 ) | 0.98 ( 0.83 ) |
| Psoriatic arthropathy | 328 | 8.14 ( 7.3 - 9.08 ) | 8.1 ( 2017.34 ) | 8.01 ( 7.31 ) | 3 ( 2.84 ) |
| Urinary tract infection | 325 | 2.11 ( 1.89 - 2.35 ) | 2.1 ( 187.59 ) | 2.1 ( 1.91 ) | 1.07 ( 0.91 ) |
| Illness | 311 | 2.91 ( 2.6 - 3.25 ) | 2.9 ( 385.31 ) | 2.89 ( 2.63 ) | 1.53 ( 1.37 ) |
| Influenza | 293 | 2.81 ( 2.51 - 3.16 ) | 2.81 ( 339.55 ) | 2.8 ( 2.54 ) | 1.48 ( 1.32 ) |
| Cellulitis | 261 | 6.08 ( 5.38 - 6.87 ) | 6.06 ( 1093.16 ) | 6.01 ( 5.43 ) | 2.59 ( 2.41 ) |
| Underdose | 241 | 3.16 ( 2.78 - 3.58 ) | 3.15 ( 352.03 ) | 3.14 ( 2.82 ) | 1.65 ( 1.46 ) |
| Ear infection | 213 | 8.66 ( 7.56 - 9.91 ) | 8.63 ( 1417.93 ) | 8.53 ( 7.61 ) | 3.09 ( 2.89 ) |
| Accidental underdose | 196 | 13.22 ( 11.48 - 15.23 ) | 13.18 ( 2162.69 ) | 12.94 ( 11.49 ) | 3.69 ( 3.49 ) |
| Upper respiratory tract infection | 185 | 4.51 ( 3.9 - 5.21 ) | 4.5 ( 500.14 ) | 4.47 ( 3.96 ) | 2.16 ( 1.95 ) |
| Oropharyngeal pain | 179 | 2.12 ( 1.83 - 2.45 ) | 2.11 ( 104.99 ) | 2.11 ( 1.87 ) | 1.08 ( 0.86 ) |
| Bronchitis | 175 | 2.76 ( 2.38 - 3.21 ) | 2.76 ( 195.63 ) | 2.75 ( 2.43 ) | 1.46 ( 1.24 ) |
| Fungal infection | 172 | 5.84 ( 5.02 - 6.78 ) | 5.82 ( 681.08 ) | 5.78 ( 5.1 ) | 2.53 ( 2.31 ) |
| Arthritis | 165 | 2.35 ( 2.02 - 2.74 ) | 2.35 ( 127.65 ) | 2.35 ( 2.06 ) | 1.23 ( 1 ) |
| Injection site induration | 158 | 17.52 ( 14.96 - 20.52 ) | 17.47 ( 2390.1 ) | 17.04 ( 14.93 ) | 4.09 ( 3.86 ) |
| Therapy non-responder | 150 | 2.97 ( 2.53 - 3.49 ) | 2.97 ( 194.7 ) | 2.96 ( 2.58 ) | 1.56 ( 1.33 ) |
| Herpes zoster | 135 | 2.53 ( 2.14 - 3 ) | 2.53 ( 124.68 ) | 2.53 ( 2.19 ) | 1.34 ( 1.09 ) |
| Oral candidiasis | 134 | 12.94 ( 10.9 - 15.35 ) | 12.91 ( 1443.68 ) | 12.68 ( 10.98 ) | 3.66 ( 3.41 ) |
| Candida infection | 125 | 7.24 ( 6.07 - 8.63 ) | 7.22 ( 663.12 ) | 7.16 ( 6.17 ) | 2.84 ( 2.58 ) |
| Swelling | 124 | 1.4 ( 1.17 - 1.67 ) | 1.4 ( 14.18 ) | 1.4 ( 1.21 ) | 0.48 ( 0.23 ) |
| Injection site discomfort | 120 | 13.06 ( 10.9 - 15.65 ) | 13.03 ( 1307.14 ) | 12.8 ( 11 ) | 3.68 ( 3.41 ) |
| Influenza like illness | 118 | 1.92 ( 1.6 - 2.3 ) | 1.92 ( 51.63 ) | 1.91 ( 1.65 ) | 0.94 ( 0.67 ) |
| Maternal exposure during pregnancy | 115 | 1.23 ( 1.02 - 1.47 ) | 1.22 ( 4.75 ) | 1.22 ( 1.05 ) | 0.29 ( 0.02 ) |
| Myocardial infarction | 113 | 1.26 ( 1.05 - 1.52 ) | 1.26 ( 6.22 ) | 1.26 ( 1.08 ) | 0.34 ( 0.07 ) |
| Pharyngitis streptococcal | 111 | 11.46 ( 9.49 - 13.82 ) | 11.43 ( 1038.89 ) | 11.25 ( 9.62 ) | 3.49 ( 3.22 ) |
| Extra dose administered | 102 | 2.72 ( 2.24 - 3.3 ) | 2.71 ( 110.07 ) | 2.71 ( 2.3 ) | 1.44 ( 1.15 ) |
| Stress | 101 | 1.59 ( 1.31 - 1.94 ) | 1.59 ( 22.24 ) | 1.59 ( 1.35 ) | 0.67 ( 0.38 ) |
| Colitis ulcerative | 100 | 2.34 ( 1.92 - 2.85 ) | 2.34 ( 76.15 ) | 2.33 ( 1.98 ) | 1.22 ( 0.93 ) |
| Inflammatory bowel disease | 98 | 20.41 ( 16.69 - 24.97 ) | 20.38 ( 1751.5 ) | 19.79 ( 16.73 ) | 4.31 ( 4.01 ) |
| Therapeutic product effect decreased | 97 | 1.72 ( 1.41 - 2.1 ) | 1.72 ( 29.28 ) | 1.72 ( 1.46 ) | 0.78 ( 0.49 ) |
| Colitis | 94 | 2.72 ( 2.22 - 3.33 ) | 2.72 ( 101.63 ) | 2.71 ( 2.29 ) | 1.44 ( 1.14 ) |
| Staphylococcal infection | 93 | 3.83 ( 3.12 - 4.69 ) | 3.82 ( 192.71 ) | 3.81 ( 3.21 ) | 1.93 ( 1.63 ) |
| Surgery | 92 | 1.83 ( 1.49 - 2.25 ) | 1.83 ( 34.55 ) | 1.83 ( 1.54 ) | 0.87 ( 0.57 ) |
| Nephrolithiasis | 91 | 2.24 ( 1.82 - 2.75 ) | 2.23 ( 61.9 ) | 2.23 ( 1.88 ) | 1.16 ( 0.86 ) |
| Intentional dose omission | 86 | 3.2 ( 2.59 - 3.95 ) | 3.19 ( 129.07 ) | 3.18 ( 2.67 ) | 1.67 ( 1.36 ) |
| Crohn's disease | 84 | 1.38 ( 1.11 - 1.71 ) | 1.38 ( 8.79 ) | 1.38 ( 1.15 ) | 0.46 ( 0.15 ) |
| Irritable bowel syndrome | 83 | 4.65 ( 3.74 - 5.77 ) | 4.64 ( 235.56 ) | 4.62 ( 3.85 ) | 2.21 ( 1.89 ) |
| Localised infection | 83 | 3.69 ( 2.97 - 4.58 ) | 3.69 ( 161.57 ) | 3.67 ( 3.06 ) | 1.88 ( 1.56 ) |
| Neoplasm malignant | 83 | 1.27 ( 1.03 - 1.58 ) | 1.27 ( 4.89 ) | 1.27 ( 1.06 ) | 0.35 ( 0.03 ) |
| Tooth infection | 79 | 6.62 ( 5.3 - 8.26 ) | 6.61 ( 372.37 ) | 6.55 ( 5.44 ) | 2.71 ( 2.39 ) |
| Eczema | 78 | 1.92 ( 1.53 - 2.39 ) | 1.91 ( 33.99 ) | 1.91 ( 1.59 ) | 0.93 ( 0.61 ) |
| Burning sensation | 76 | 1.32 ( 1.05 - 1.65 ) | 1.32 ( 5.74 ) | 1.31 ( 1.09 ) | 0.39 ( 0.07 ) |
| Blister | 75 | 1.53 ( 1.22 - 1.92 ) | 1.53 ( 13.62 ) | 1.53 ( 1.26 ) | 0.61 ( 0.28 ) |
| Injection site inflammation | 74 | 18.75 ( 14.88 - 23.63 ) | 18.73 ( 1207.44 ) | 18.24 ( 15.03 ) | 4.19 ( 3.85 ) |
| Injection site irritation | 71 | 12.23 ( 9.67 - 15.47 ) | 12.22 ( 717.79 ) | 12.01 ( 9.87 ) | 3.59 ( 3.24 ) |
| Viral infection | 70 | 2.49 ( 1.97 - 3.14 ) | 2.48 ( 61.88 ) | 2.48 ( 2.04 ) | 1.31 ( 0.97 ) |
| Skin disorder | 67 | 2.17 ( 1.71 - 2.76 ) | 2.17 ( 42.03 ) | 2.16 ( 1.77 ) | 1.11 ( 0.76 ) |
| Respiratory tract infection | 66 | 2.74 ( 2.15 - 3.49 ) | 2.74 ( 72.59 ) | 2.73 ( 2.23 ) | 1.45 ( 1.1 ) |
| Injury associated with device | 65 | 3.17 ( 2.48 - 4.05 ) | 3.17 ( 96.04 ) | 3.16 ( 2.58 ) | 1.66 ( 1.3 ) |
| Product dispensing error | 65 | 2.45 ( 1.92 - 3.13 ) | 2.45 ( 55.65 ) | 2.45 ( 1.99 ) | 1.29 ( 0.93 ) |
| Sars-cov-2 test positive | 65 | 3.44 ( 2.7 - 4.39 ) | 3.44 ( 111.75 ) | 3.42 ( 2.79 ) | 1.78 ( 1.42 ) |
| Inflammation | 64 | 1.34 ( 1.05 - 1.71 ) | 1.34 ( 5.39 ) | 1.34 ( 1.09 ) | 0.42 ( 0.06 ) |
| Diverticulitis | 64 | 2.54 ( 1.99 - 3.25 ) | 2.54 ( 59.41 ) | 2.53 ( 2.06 ) | 1.34 ( 0.98 ) |
| Cystitis | 62 | 2.23 ( 1.74 - 2.86 ) | 2.23 ( 41.78 ) | 2.22 ( 1.8 ) | 1.15 ( 0.79 ) |
| Immune system disorder | 60 | 4.84 ( 3.75 - 6.24 ) | 4.83 ( 181.03 ) | 4.8 ( 3.88 ) | 2.26 ( 1.89 ) |
| Injection site hypersensitivity | 59 | 22.43 ( 17.3 - 29.08 ) | 22.4 ( 1166.53 ) | 21.69 ( 17.46 ) | 4.44 ( 4.06 ) |
| Angioedema | 59 | 1.58 ( 1.22 - 2.04 ) | 1.58 ( 12.54 ) | 1.58 ( 1.27 ) | 0.66 ( 0.29 ) |
| Lymphadenopathy | 56 | 1.92 ( 1.48 - 2.49 ) | 1.92 ( 24.53 ) | 1.91 ( 1.54 ) | 0.94 ( 0.55 ) |
| Kidney infection | 53 | 2.87 ( 2.19 - 3.76 ) | 2.87 ( 64.13 ) | 2.86 ( 2.28 ) | 1.51 ( 1.12 ) |
| Conjunctivitis | 52 | 2.95 ( 2.25 - 3.88 ) | 2.95 ( 66.69 ) | 2.94 ( 2.34 ) | 1.56 ( 1.16 ) |
| Device defective | 52 | 3.94 ( 3 - 5.18 ) | 3.94 ( 113.3 ) | 3.92 ( 3.12 ) | 1.97 ( 1.57 ) |
| Tuberculosis | 52 | 4.54 ( 3.45 - 5.96 ) | 4.54 ( 142.36 ) | 4.51 ( 3.59 ) | 2.17 ( 1.78 ) |
| Injection site discolouration | 51 | 5.31 ( 4.03 - 6.99 ) | 5.3 ( 176.66 ) | 5.27 ( 4.18 ) | 2.4 ( 2 ) |
| Therapeutic response shortened | 51 | 1.54 ( 1.17 - 2.03 ) | 1.54 ( 9.6 ) | 1.54 ( 1.22 ) | 0.62 ( 0.22 ) |
| Accidental overdose | 50 | 1.71 ( 1.29 - 2.25 ) | 1.71 ( 14.6 ) | 1.7 ( 1.35 ) | 0.77 ( 0.36 ) |
| Skin infection | 48 | 4.58 ( 3.44 - 6.08 ) | 4.57 ( 133.09 ) | 4.55 ( 3.59 ) | 2.19 ( 1.77 ) |
| Ankylosing spondylitis | 48 | 3.56 ( 2.68 - 4.73 ) | 3.56 ( 87.81 ) | 3.54 ( 2.79 ) | 1.83 ( 1.41 ) |
| Gastroenteritis viral | 47 | 2.95 ( 2.22 - 3.93 ) | 2.95 ( 60.38 ) | 2.94 ( 2.31 ) | 1.56 ( 1.14 ) |
| Lip swelling | 46 | 1.69 ( 1.26 - 2.25 ) | 1.68 ( 12.78 ) | 1.68 ( 1.32 ) | 0.75 ( 0.33 ) |
| Malignant melanoma | 46 | 3.55 ( 2.66 - 4.74 ) | 3.55 ( 83.71 ) | 3.53 ( 2.77 ) | 1.82 ( 1.4 ) |
| Injection site cellulitis | 44 | 35.4 ( 26.13 - 47.95 ) | 35.37 ( 1394.11 ) | 33.61 ( 26.07 ) | 5.07 ( 4.63 ) |
| Bacterial infection | 43 | 2.72 ( 2.01 - 3.67 ) | 2.72 ( 46.42 ) | 2.71 ( 2.11 ) | 1.44 ( 1 ) |
| Injection site injury | 43 | 8.84 ( 6.54 - 11.95 ) | 8.84 ( 294.82 ) | 8.73 ( 6.79 ) | 3.13 ( 2.69 ) |
| Spinal operation | 43 | 3.22 ( 2.39 - 4.35 ) | 3.22 ( 65.47 ) | 3.21 ( 2.5 ) | 1.68 ( 1.25 ) |
| Eye infection | 42 | 3.92 ( 2.89 - 5.31 ) | 3.91 ( 90.61 ) | 3.9 ( 3.02 ) | 1.96 ( 1.52 ) |
| Knee arthroplasty | 41 | 2.18 ( 1.61 - 2.96 ) | 2.18 ( 26.12 ) | 2.18 ( 1.68 ) | 1.12 ( 0.68 ) |
| Mycobacterium tuberculosis complex test positive | 40 | 15.72 ( 11.49 - 21.51 ) | 15.71 ( 538.05 ) | 15.36 ( 11.82 ) | 3.94 ( 3.49 ) |
| Swollen tongue | 36 | 1.55 ( 1.12 - 2.15 ) | 1.55 ( 6.97 ) | 1.55 ( 1.18 ) | 0.63 ( 0.15 ) |
| Insurance issue | 36 | 2.5 ( 1.8 - 3.47 ) | 2.5 ( 32.25 ) | 2.49 ( 1.9 ) | 1.32 ( 0.84 ) |
| Pharyngitis | 35 | 3.16 ( 2.27 - 4.41 ) | 3.16 ( 51.55 ) | 3.15 ( 2.39 ) | 1.66 ( 1.17 ) |
| Pustular psoriasis | 33 | 13.31 ( 9.43 - 18.79 ) | 13.31 ( 368.08 ) | 13.06 ( 9.79 ) | 3.71 ( 3.21 ) |
| Mouth ulceration | 33 | 1.82 ( 1.29 - 2.56 ) | 1.82 ( 12.18 ) | 1.82 ( 1.37 ) | 0.86 ( 0.37 ) |
| Throat tightness | 33 | 1.54 ( 1.09 - 2.17 ) | 1.54 ( 6.22 ) | 1.54 ( 1.16 ) | 0.62 ( 0.13 ) |
| Streptococcal infection | 33 | 7.29 ( 5.17 - 10.28 ) | 7.29 ( 177.13 ) | 7.22 ( 5.42 ) | 2.85 ( 2.35 ) |
| Mass | 32 | 2.49 ( 1.76 - 3.53 ) | 2.49 ( 28.53 ) | 2.49 ( 1.86 ) | 1.32 ( 0.81 ) |
| Aphthous ulcer | 31 | 3.28 ( 2.3 - 4.67 ) | 3.28 ( 48.83 ) | 3.27 ( 2.43 ) | 1.71 ( 1.2 ) |
| Injection site vesicles | 31 | 6.24 ( 4.38 - 8.88 ) | 6.23 ( 134.95 ) | 6.18 ( 4.6 ) | 2.63 ( 2.12 ) |
| Abscess | 30 | 2.22 ( 1.55 - 3.17 ) | 2.22 ( 19.98 ) | 2.21 ( 1.64 ) | 1.15 ( 0.63 ) |
| Sinus disorder | 28 | 1.54 ( 1.07 - 2.24 ) | 1.54 ( 5.35 ) | 1.54 ( 1.13 ) | 0.63 ( 0.09 ) |
| Furuncle | 27 | 3.89 ( 2.66 - 5.68 ) | 3.89 ( 57.57 ) | 3.87 ( 2.82 ) | 1.95 ( 1.41 ) |
| Wrong schedule | 27 | 21.27 ( 14.5 - 31.21 ) | 21.26 ( 504.87 ) | 20.62 ( 14.96 ) | 4.37 ( 3.81 ) |
| Injection site oedema | 26 | 20 ( 13.54 - 29.55 ) | 19.99 ( 455.13 ) | 19.43 ( 14.01 ) | 4.28 ( 3.72 ) |
| Pulmonary congestion | 26 | 2.5 ( 1.7 - 3.68 ) | 2.5 ( 23.31 ) | 2.49 ( 1.81 ) | 1.32 ( 0.76 ) |
| Fear of injection | 25 | 3.48 ( 2.35 - 5.16 ) | 3.48 ( 44 ) | 3.47 ( 2.5 ) | 1.79 ( 1.23 ) |
| Osteomyelitis | 25 | 1.67 ( 1.13 - 2.47 ) | 1.67 ( 6.65 ) | 1.67 ( 1.2 ) | 0.74 ( 0.17 ) |
| Multiple allergies | 25 | 3.4 ( 2.29 - 5.03 ) | 3.4 ( 42.07 ) | 3.38 ( 2.44 ) | 1.76 ( 1.19 ) |
| Tooth abscess | 24 | 2.84 ( 1.9 - 4.24 ) | 2.84 ( 28.46 ) | 2.83 ( 2.02 ) | 1.5 ( 0.92 ) |
| Erysipelas | 24 | 5.23 ( 3.5 - 7.81 ) | 5.23 ( 81.36 ) | 5.19 ( 3.71 ) | 2.38 ( 1.8 ) |
| Skin plaque | 24 | 2.78 ( 1.86 - 4.16 ) | 2.78 ( 27.28 ) | 2.77 ( 1.98 ) | 1.47 ( 0.89 ) |
| Tinea infection | 24 | 26.34 ( 17.51 - 39.62 ) | 26.33 ( 562.18 ) | 25.35 ( 18.01 ) | 4.66 ( 4.08 ) |
| Folliculitis | 23 | 2.4 ( 1.59 - 3.61 ) | 2.4 ( 18.67 ) | 2.39 ( 1.7 ) | 1.26 ( 0.67 ) |
| Fungal skin infection | 23 | 6.04 ( 4 - 9.1 ) | 6.03 ( 95.73 ) | 5.99 ( 4.25 ) | 2.58 ( 1.99 ) |
| Cardiac operation | 23 | 3.22 ( 2.13 - 4.84 ) | 3.21 ( 34.92 ) | 3.2 ( 2.27 ) | 1.68 ( 1.09 ) |
| Pharyngeal swelling | 23 | 2.02 ( 1.34 - 3.04 ) | 2.02 ( 11.74 ) | 2.01 ( 1.43 ) | 1.01 ( 0.42 ) |
| Decreased immune responsiveness | 23 | 2.16 ( 1.44 - 3.26 ) | 2.16 ( 14.33 ) | 2.16 ( 1.53 ) | 1.11 ( 0.52 ) |
| Skin reaction | 22 | 1.76 ( 1.16 - 2.67 ) | 1.76 ( 7.2 ) | 1.76 ( 1.24 ) | 0.81 ( 0.21 ) |
| Lymphoma | 22 | 1.58 ( 1.04 - 2.41 ) | 1.58 ( 4.71 ) | 1.58 ( 1.11 ) | 0.66 ( 0.06 ) |
| Arthropod bite | 22 | 3.44 ( 2.26 - 5.23 ) | 3.44 ( 37.86 ) | 3.43 ( 2.41 ) | 1.78 ( 1.17 ) |
| Therapy change | 22 | 2.11 ( 1.39 - 3.21 ) | 2.11 ( 12.83 ) | 2.11 ( 1.49 ) | 1.08 ( 0.47 ) |
| Diarrhoea haemorrhagic | 22 | 2.68 ( 1.77 - 4.08 ) | 2.68 ( 23.14 ) | 2.68 ( 1.89 ) | 1.42 ( 0.82 ) |
| Injection related reaction | 21 | 11.97 ( 7.77 - 18.43 ) | 11.96 ( 207.17 ) | 11.76 ( 8.2 ) | 3.56 ( 2.94 ) |
| Cyst | 21 | 1.92 ( 1.25 - 2.95 ) | 1.92 ( 9.28 ) | 1.92 ( 1.34 ) | 0.94 ( 0.33 ) |
| Vasculitis | 21 | 2.26 ( 1.47 - 3.47 ) | 2.26 ( 14.73 ) | 2.26 ( 1.58 ) | 1.17 ( 0.56 ) |
| Scratch | 21 | 2.3 ( 1.5 - 3.53 ) | 2.3 ( 15.39 ) | 2.3 ( 1.6 ) | 1.2 ( 0.58 ) |
| Post procedural infection | 20 | 2.57 ( 1.65 - 3.98 ) | 2.57 ( 19.06 ) | 2.56 ( 1.77 ) | 1.36 ( 0.73 ) |
| Oral fungal infection | 20 | 9.2 ( 5.92 - 14.31 ) | 9.2 ( 144.16 ) | 9.09 ( 6.28 ) | 3.18 ( 2.55 ) |
| Oesophageal candidiasis | 20 | 6.27 ( 4.03 - 9.73 ) | 6.26 ( 87.65 ) | 6.21 ( 4.3 ) | 2.64 ( 2 ) |
| Scab | 19 | 1.8 ( 1.15 - 2.82 ) | 1.8 ( 6.74 ) | 1.8 ( 1.23 ) | 0.85 ( 0.2 ) |
| Laryngitis | 19 | 2.14 ( 1.36 - 3.35 ) | 2.14 ( 11.45 ) | 2.13 ( 1.46 ) | 1.09 ( 0.45 ) |
| Immunosuppression | 19 | 2.32 ( 1.48 - 3.64 ) | 2.32 ( 14.25 ) | 2.32 ( 1.59 ) | 1.21 ( 0.57 ) |
| Knee operation | 19 | 2.14 ( 1.36 - 3.35 ) | 2.14 ( 11.45 ) | 2.13 ( 1.46 ) | 1.09 ( 0.45 ) |
| Antibiotic therapy | 19 | 16.97 ( 10.76 - 26.77 ) | 16.97 ( 278.26 ) | 16.56 ( 11.31 ) | 4.05 ( 3.4 ) |
| Hordeolum | 18 | 4.38 ( 2.76 - 6.97 ) | 4.38 ( 46.69 ) | 4.36 ( 2.96 ) | 2.12 ( 1.46 ) |
| Injection site nodule | 17 | 2.21 ( 1.37 - 3.56 ) | 2.21 ( 11.21 ) | 2.2 ( 1.48 ) | 1.14 ( 0.46 ) |
| Tonsillitis | 17 | 3.46 ( 2.15 - 5.57 ) | 3.46 ( 29.55 ) | 3.45 ( 2.31 ) | 1.78 ( 1.1 ) |
| Product distribution issue | 17 | 2.23 ( 1.38 - 3.59 ) | 2.23 ( 11.46 ) | 2.22 ( 1.49 ) | 1.15 ( 0.47 ) |
| Hernia repair | 17 | 8.35 ( 5.18 - 13.48 ) | 8.35 ( 108.6 ) | 8.26 ( 5.53 ) | 3.05 ( 2.36 ) |
| Skin laceration | 17 | 1.62 ( 1 - 2.6 ) | 1.61 ( 3.97 ) | 1.61 ( 1.08 ) | 0.69 ( 0.01 ) |
| Serum sickness | 16 | 8.58 ( 5.24 - 14.05 ) | 8.58 ( 105.68 ) | 8.48 ( 5.61 ) | 3.08 ( 2.38 ) |
| Injection site scar | 16 | 6.42 ( 3.92 - 10.5 ) | 6.42 ( 72.43 ) | 6.36 ( 4.21 ) | 2.67 ( 1.97 ) |
| Vitiligo | 16 | 7.2 ( 4.4 - 11.79 ) | 7.2 ( 84.54 ) | 7.14 ( 4.72 ) | 2.83 ( 2.13 ) |
| Cholecystectomy | 16 | 2.24 ( 1.37 - 3.66 ) | 2.24 ( 10.92 ) | 2.23 ( 1.48 ) | 1.16 ( 0.46 ) |
| Oropharyngeal discomfort | 16 | 1.77 ( 1.08 - 2.89 ) | 1.77 ( 5.36 ) | 1.77 ( 1.17 ) | 0.82 ( 0.12 ) |
| Coronavirus infection | 16 | 1.75 ( 1.07 - 2.86 ) | 1.75 ( 5.13 ) | 1.75 ( 1.16 ) | 0.81 ( 0.11 ) |
| Tinea pedis | 15 | 12.34 ( 7.4 - 20.56 ) | 12.33 ( 153.3 ) | 12.12 ( 7.91 ) | 3.6 ( 2.87 ) |
| Pharyngeal oedema | 15 | 1.7 ( 1.03 - 2.83 ) | 1.7 ( 4.36 ) | 1.7 ( 1.11 ) | 0.77 ( 0.05 ) |
| Swelling of eyelid | 15 | 2.18 ( 1.31 - 3.62 ) | 2.18 ( 9.53 ) | 2.17 ( 1.42 ) | 1.12 ( 0.4 ) |
| Impetigo | 14 | 11.78 ( 6.94 - 19.98 ) | 11.78 ( 135.62 ) | 11.59 ( 7.44 ) | 3.53 ( 2.78 ) |
| Pyoderma gangrenosum | 14 | 4.18 ( 2.47 - 7.06 ) | 4.17 ( 33.59 ) | 4.15 ( 2.68 ) | 2.05 ( 1.31 ) |
| Skin mass | 14 | 2.13 ( 1.26 - 3.6 ) | 2.13 ( 8.37 ) | 2.13 ( 1.37 ) | 1.09 ( 0.34 ) |
| Colitis microscopic | 14 | 2.87 ( 1.7 - 4.86 ) | 2.87 ( 17.01 ) | 2.86 ( 1.85 ) | 1.52 ( 0.77 ) |
| Guillain-barre syndrome | 14 | 3.55 ( 2.1 - 6 ) | 3.55 ( 25.48 ) | 3.53 ( 2.28 ) | 1.82 ( 1.07 ) |
| Wrong dose | 14 | 4.59 ( 2.72 - 7.77 ) | 4.59 ( 39.07 ) | 4.57 ( 2.94 ) | 2.19 ( 1.44 ) |
| Injection site infection | 13 | 5.29 ( 3.07 - 9.13 ) | 5.29 ( 44.87 ) | 5.26 ( 3.33 ) | 2.39 ( 1.62 ) |
| Hand fracture | 13 | 2.12 ( 1.23 - 3.65 ) | 2.12 ( 7.63 ) | 2.11 ( 1.34 ) | 1.08 ( 0.31 ) |
| Injection site plaque | 13 | 62.87 ( 35.58 - 111.09 ) | 62.86 ( 721.87 ) | 57.43 ( 35.67 ) | 5.84 ( 5.04 ) |
| Enthesopathy | 13 | 6.53 ( 3.78 - 11.28 ) | 6.53 ( 60.25 ) | 6.47 ( 4.1 ) | 2.69 ( 1.92 ) |
| Gallbladder operation | 13 | 4.33 ( 2.51 - 7.46 ) | 4.33 ( 33.02 ) | 4.3 ( 2.73 ) | 2.11 ( 1.33 ) |
| Rhinitis | 13 | 1.82 ( 1.06 - 3.13 ) | 1.82 ( 4.77 ) | 1.82 ( 1.15 ) | 0.86 ( 0.09 ) |
| Lichen planus | 13 | 7.11 ( 4.12 - 12.28 ) | 7.11 ( 67.49 ) | 7.04 ( 4.46 ) | 2.82 ( 2.04 ) |
| Manufacturing product shipping issue | 12 | 17.65 ( 9.94 - 31.31 ) | 17.64 ( 183.44 ) | 17.2 ( 10.65 ) | 4.1 ( 3.29 ) |
| Intestinal haemorrhage | 12 | 2.57 ( 1.46 - 4.54 ) | 2.57 ( 11.5 ) | 2.57 ( 1.6 ) | 1.36 ( 0.56 ) |
| Shoulder operation | 12 | 3.16 ( 1.79 - 5.57 ) | 3.16 ( 17.63 ) | 3.15 ( 1.96 ) | 1.66 ( 0.85 ) |
| Stent placement | 12 | 2.08 ( 1.18 - 3.67 ) | 2.08 ( 6.72 ) | 2.08 ( 1.29 ) | 1.06 ( 0.25 ) |
| Vulvovaginal mycotic infection | 12 | 3.95 ( 2.24 - 6.98 ) | 3.95 ( 26.33 ) | 3.94 ( 2.45 ) | 1.98 ( 1.17 ) |
| Emergency care | 12 | 3.48 ( 1.97 - 6.14 ) | 3.48 ( 21.1 ) | 3.47 ( 2.16 ) | 1.79 ( 0.99 ) |
| Treatment delayed | 12 | 8.69 ( 4.91 - 15.35 ) | 8.68 ( 80.53 ) | 8.58 ( 5.33 ) | 3.1 ( 2.3 ) |
| Subcutaneous abscess | 11 | 2.64 ( 1.46 - 4.78 ) | 2.64 ( 11.17 ) | 2.63 ( 1.6 ) | 1.4 ( 0.56 ) |
| Rash pustular | 11 | 2.11 ( 1.17 - 3.82 ) | 2.11 ( 6.43 ) | 2.11 ( 1.29 ) | 1.08 ( 0.24 ) |
| Sarcoidosis | 11 | 2.76 ( 1.53 - 5 ) | 2.76 ( 12.33 ) | 2.76 ( 1.68 ) | 1.46 ( 0.63 ) |
| Gingivitis | 11 | 2.57 ( 1.42 - 4.65 ) | 2.57 ( 10.54 ) | 2.57 ( 1.56 ) | 1.36 ( 0.53 ) |
| Dermatitis exfoliative generalised | 11 | 2.75 ( 1.52 - 4.97 ) | 2.75 ( 12.21 ) | 2.74 ( 1.67 ) | 1.46 ( 0.62 ) |
| Oral disorder | 11 | 1.81 ( 1 - 3.26 ) | 1.81 ( 3.94 ) | 1.8 ( 1.1 ) | 0.85 ( 0.02 ) |
| Guttate psoriasis | 10 | 13.89 ( 7.42 - 25.99 ) | 13.89 ( 117.12 ) | 13.62 ( 8.06 ) | 3.77 ( 2.89 ) |
| Overweight | 10 | 1.84 ( 0.99 - 3.43 ) | 1.84 ( 3.85 ) | 1.84 ( 1.1 ) | 0.88 ( 0.01 ) |
| Dactylitis | 10 | 8.37 ( 4.49 - 15.62 ) | 8.37 ( 64.06 ) | 8.28 ( 4.91 ) | 3.05 ( 2.17 ) |
| Necrotising fasciitis | 10 | 3.37 ( 1.81 - 6.27 ) | 3.37 ( 16.55 ) | 3.35 ( 1.99 ) | 1.75 ( 0.87 ) |
| Herpes virus infection | 10 | 2.09 ( 1.13 - 3.89 ) | 2.09 ( 5.69 ) | 2.09 ( 1.24 ) | 1.06 ( 0.19 ) |
| Latent tuberculosis | 10 | 4.69 ( 2.52 - 8.74 ) | 4.69 ( 28.85 ) | 4.67 ( 2.77 ) | 2.22 ( 1.35 ) |
| Anal abscess | 10 | 1.88 ( 1.01 - 3.5 ) | 1.88 ( 4.12 ) | 1.88 ( 1.12 ) | 0.91 ( 0.04 ) |
| Injection site discharge | 10 | 2.93 ( 1.57 - 5.45 ) | 2.93 ( 12.64 ) | 2.92 ( 1.74 ) | 1.55 ( 0.67 ) |
| Tuberculin test positive | 10 | 5.34 ( 2.86 - 9.95 ) | 5.34 ( 34.96 ) | 5.3 ( 3.15 ) | 2.41 ( 1.53 ) |
| Neck surgery | 10 | 4.22 ( 2.27 - 7.86 ) | 4.22 ( 24.41 ) | 4.2 ( 2.5 ) | 2.07 ( 1.2 ) |
| Dyshidrotic eczema | 10 | 17.69 ( 9.44 - 33.16 ) | 17.69 ( 153.32 ) | 17.25 ( 10.2 ) | 4.11 ( 3.23 ) |
| Palmoplantar pustulosis | 10 | 15.73 ( 8.4 - 29.46 ) | 15.73 ( 134.69 ) | 15.38 ( 9.1 ) | 3.94 ( 3.06 ) |
| Staphylococcal skin infection | 9 | 9.37 ( 4.85 - 18.1 ) | 9.37 ( 66.34 ) | 9.25 ( 5.33 ) | 3.21 ( 2.29 ) |
| Cutaneous vasculitis | 9 | 2.83 ( 1.47 - 5.45 ) | 2.83 ( 10.63 ) | 2.82 ( 1.63 ) | 1.5 ( 0.58 ) |
| Erythrodermic psoriasis | 9 | 14.84 ( 7.66 - 28.73 ) | 14.84 ( 113.55 ) | 14.53 ( 8.36 ) | 3.86 ( 2.94 ) |
| Injection site paraesthesia | 9 | 6.79 ( 3.52 - 13.1 ) | 6.79 ( 44 ) | 6.73 ( 3.89 ) | 2.75 ( 1.83 ) |
| Injection site papule | 9 | 2.41 ( 1.25 - 4.63 ) | 2.4 ( 7.36 ) | 2.4 ( 1.39 ) | 1.26 ( 0.35 ) |
| Coronary arterial stent insertion | 9 | 3.95 ( 2.05 - 7.61 ) | 3.95 ( 19.72 ) | 3.93 ( 2.27 ) | 1.98 ( 1.06 ) |
| Appendicectomy | 9 | 3.34 ( 1.74 - 6.43 ) | 3.34 ( 14.7 ) | 3.33 ( 1.92 ) | 1.74 ( 0.82 ) |
| Tongue ulceration | 9 | 3.39 ( 1.76 - 6.52 ) | 3.39 ( 15.07 ) | 3.38 ( 1.95 ) | 1.76 ( 0.84 ) |
| Loss of therapeutic response | 9 | 3.26 ( 1.69 - 6.28 ) | 3.26 ( 14.03 ) | 3.25 ( 1.88 ) | 1.7 ( 0.78 ) |
| Alopecia areata | 8 | 4.24 ( 2.12 - 8.5 ) | 4.24 ( 19.7 ) | 4.22 ( 2.36 ) | 2.08 ( 1.11 ) |
| Ear swelling | 8 | 3.68 ( 1.83 - 7.36 ) | 3.68 ( 15.49 ) | 3.66 ( 2.05 ) | 1.87 ( 0.91 ) |
| Rebound psoriasis | 8 | 10.28 ( 5.11 - 20.67 ) | 10.28 ( 65.98 ) | 10.14 ( 5.65 ) | 3.34 ( 2.37 ) |
| Oral infection | 8 | 2.69 ( 1.34 - 5.38 ) | 2.69 ( 8.43 ) | 2.68 ( 1.5 ) | 1.42 ( 0.46 ) |
| Skin neoplasm excision | 8 | 10.93 ( 5.43 - 21.98 ) | 10.93 ( 70.95 ) | 10.76 ( 6 ) | 3.43 ( 2.46 ) |
| Seborrhoeic dermatitis | 8 | 5.82 ( 2.9 - 11.67 ) | 5.82 ( 31.62 ) | 5.77 ( 3.22 ) | 2.53 ( 1.56 ) |
| Miliaria | 8 | 4.64 ( 2.31 - 9.3 ) | 4.64 ( 22.67 ) | 4.61 ( 2.58 ) | 2.21 ( 1.24 ) |
| Cheilitis | 8 | 2.12 ( 1.06 - 4.24 ) | 2.12 ( 4.71 ) | 2.12 ( 1.18 ) | 1.08 ( 0.12 ) |
| Gastric infection | 8 | 2.11 ( 1.05 - 4.22 ) | 2.11 ( 4.65 ) | 2.11 ( 1.18 ) | 1.07 ( 0.11 ) |
| Intracranial aneurysm | 8 | 2.56 ( 1.28 - 5.13 ) | 2.56 ( 7.58 ) | 2.56 ( 1.43 ) | 1.35 ( 0.39 ) |
| Cutaneous t-cell lymphoma | 8 | 6.59 ( 3.29 - 13.23 ) | 6.59 ( 37.58 ) | 6.54 ( 3.65 ) | 2.71 ( 1.74 ) |
| Lyme disease | 8 | 2.68 ( 1.34 - 5.37 ) | 2.68 ( 8.41 ) | 2.68 ( 1.5 ) | 1.42 ( 0.46 ) |
| Pustule | 8 | 3.12 ( 1.56 - 6.25 ) | 3.12 ( 11.47 ) | 3.11 ( 1.74 ) | 1.64 ( 0.67 ) |
| Pruritus genital | 7 | 3.1 ( 1.48 - 6.52 ) | 3.1 ( 9.91 ) | 3.09 ( 1.66 ) | 1.63 ( 0.6 ) |
| Injection site hypoaesthesia | 7 | 4.8 ( 2.28 - 10.09 ) | 4.79 ( 20.87 ) | 4.77 ( 2.56 ) | 2.25 ( 1.23 ) |
| Tinea cruris | 7 | 16.62 ( 7.85 - 35.19 ) | 16.62 ( 100.18 ) | 16.23 ( 8.66 ) | 4.02 ( 2.99 ) |
| Otorrhoea | 7 | 4.55 ( 2.16 - 9.56 ) | 4.55 ( 19.23 ) | 4.52 ( 2.43 ) | 2.18 ( 1.15 ) |
| Lip pain | 7 | 2.96 ( 1.41 - 6.22 ) | 2.96 ( 9.03 ) | 2.95 ( 1.58 ) | 1.56 ( 0.54 ) |
| Renal cell carcinoma | 7 | 2.09 ( 0.99 - 4.38 ) | 2.09 ( 3.94 ) | 2.08 ( 1.12 ) | 1.06 ( 0.04 ) |
| Hypersensitivity vasculitis | 7 | 2.37 ( 1.13 - 4.98 ) | 2.37 ( 5.54 ) | 2.37 ( 1.27 ) | 1.24 ( 0.22 ) |
| Nail pitting | 7 | 17.31 ( 8.17 - 36.67 ) | 17.31 ( 104.79 ) | 16.89 ( 9.01 ) | 4.08 ( 3.04 ) |
| Infectious mononucleosis | 7 | 4.16 ( 1.98 - 8.74 ) | 4.16 ( 16.68 ) | 4.14 ( 2.22 ) | 2.05 ( 1.02 ) |
| Sialoadenitis | 6 | 4.99 ( 2.23 - 11.14 ) | 4.99 ( 18.99 ) | 4.96 ( 2.53 ) | 2.31 ( 1.21 ) |
| Ear infection fungal | 6 | 19.11 ( 8.48 - 43.03 ) | 19.11 ( 100.02 ) | 18.59 ( 9.43 ) | 4.22 ( 3.11 ) |
| Ingrowing nail | 6 | 2.87 ( 1.29 - 6.4 ) | 2.87 ( 7.28 ) | 2.86 ( 1.46 ) | 1.52 ( 0.42 ) |
| Lung adenocarcinoma | 6 | 2.18 ( 0.98 - 4.86 ) | 2.18 ( 3.82 ) | 2.18 ( 1.11 ) | 1.12 ( 0.03 ) |
| Tonsillar hypertrophy | 6 | 4.19 ( 1.88 - 9.35 ) | 4.19 ( 14.47 ) | 4.17 ( 2.13 ) | 2.06 ( 0.96 ) |
| Cirrhosis alcoholic | 6 | 17.03 ( 7.57 - 38.31 ) | 17.03 ( 88.22 ) | 16.62 ( 8.44 ) | 4.05 ( 2.95 ) |
| Acrochordon | 6 | 4.94 ( 2.21 - 11.03 ) | 4.94 ( 18.71 ) | 4.91 ( 2.51 ) | 2.3 ( 1.2 ) |
| Therapeutic response delayed | 6 | 2.97 ( 1.33 - 6.62 ) | 2.97 ( 7.8 ) | 2.96 ( 1.51 ) | 1.57 ( 0.47 ) |
| Lip blister | 6 | 2.6 ( 1.17 - 5.79 ) | 2.6 ( 5.88 ) | 2.59 ( 1.33 ) | 1.37 ( 0.28 ) |
| Accident at work | 6 | 3.17 ( 1.42 - 7.07 ) | 3.17 ( 8.86 ) | 3.16 ( 1.61 ) | 1.66 ( 0.57 ) |
| Exfoliative rash | 6 | 3.1 ( 1.39 - 6.92 ) | 3.1 ( 8.51 ) | 3.09 ( 1.58 ) | 1.63 ( 0.54 ) |
| Fungal oesophagitis | 6 | 10.36 ( 4.63 - 23.21 ) | 10.36 ( 49.96 ) | 10.22 ( 5.2 ) | 3.35 ( 2.25 ) |
| Otitis externa | 6 | 6.01 ( 2.69 - 13.42 ) | 6.01 ( 24.81 ) | 5.96 ( 3.04 ) | 2.58 ( 1.48 ) |
| Nail psoriasis | 6 | 8.94 ( 4 - 20.02 ) | 8.94 ( 41.75 ) | 8.83 ( 4.5 ) | 3.14 ( 2.04 ) |
| Duplicate therapy error | 6 | 15.61 ( 6.94 - 35.07 ) | 15.6 ( 80.1 ) | 15.26 ( 7.75 ) | 3.93 ( 2.83 ) |
| Endometrial cancer | 6 | 3.65 ( 1.64 - 8.15 ) | 3.65 ( 11.5 ) | 3.64 ( 1.86 ) | 1.86 ( 0.77 ) |
| Coronavirus test positive | 6 | 4.05 ( 1.82 - 9.05 ) | 4.05 ( 13.72 ) | 4.04 ( 2.06 ) | 2.01 ( 0.92 ) |
| Skin bacterial infection | 6 | 8.19 ( 3.66 - 18.33 ) | 8.19 ( 37.43 ) | 8.1 ( 4.13 ) | 3.02 ( 1.92 ) |
| Exposure to sars-cov-2 | 6 | 2.4 ( 1.08 - 5.35 ) | 2.4 ( 4.89 ) | 2.4 ( 1.23 ) | 1.26 ( 0.17 ) |
| Animal bite | 6 | 2.64 ( 1.19 - 5.9 ) | 2.64 ( 6.11 ) | 2.64 ( 1.35 ) | 1.4 ( 0.31 ) |
| Local reaction | 5 | 7.83 ( 3.24 - 18.91 ) | 7.83 ( 29.42 ) | 7.75 ( 3.7 ) | 2.95 ( 1.77 ) |
| Oesophageal infection | 5 | 12.46 ( 5.14 - 30.18 ) | 12.46 ( 51.7 ) | 12.24 ( 5.84 ) | 3.61 ( 2.42 ) |
| Groin abscess | 5 | 4.36 ( 1.81 - 10.52 ) | 4.36 ( 12.88 ) | 4.34 ( 2.08 ) | 2.12 ( 0.94 ) |
| Onycholysis | 5 | 3.36 ( 1.4 - 8.09 ) | 3.36 ( 8.25 ) | 3.35 ( 1.61 ) | 1.74 ( 0.56 ) |
| Skin candida | 5 | 11.7 ( 4.83 - 28.33 ) | 11.7 ( 48.06 ) | 11.51 ( 5.49 ) | 3.52 ( 2.33 ) |
| Peritonsillar abscess | 5 | 6.63 ( 2.75 - 16.01 ) | 6.63 ( 23.68 ) | 6.58 ( 3.15 ) | 2.72 ( 1.53 ) |
| Ophthalmic herpes zoster | 5 | 2.6 ( 1.08 - 6.27 ) | 2.6 ( 4.92 ) | 2.6 ( 1.25 ) | 1.38 ( 0.2 ) |
| Nail infection | 5 | 3.84 ( 1.59 - 9.24 ) | 3.84 ( 10.42 ) | 3.82 ( 1.83 ) | 1.93 ( 0.75 ) |
| Root canal infection | 5 | 8.35 ( 3.46 - 20.17 ) | 8.35 ( 31.93 ) | 8.25 ( 3.95 ) | 3.05 ( 1.86 ) |
| Induration | 5 | 3.8 ( 1.58 - 9.14 ) | 3.8 ( 10.23 ) | 3.78 ( 1.81 ) | 1.92 ( 0.74 ) |
| Colonoscopy | 5 | 2.51 ( 1.04 - 6.03 ) | 2.51 ( 4.51 ) | 2.5 ( 1.2 ) | 1.32 ( 0.14 ) |
| Metabolic surgery | 5 | 3.59 ( 1.49 - 8.65 ) | 3.59 ( 9.29 ) | 3.58 ( 1.71 ) | 1.84 ( 0.66 ) |
| Bronchial disorder | 5 | 3.54 ( 1.47 - 8.53 ) | 3.54 ( 9.06 ) | 3.53 ( 1.69 ) | 1.82 ( 0.64 ) |
| Myelitis | 5 | 3.58 ( 1.48 - 8.61 ) | 3.57 ( 9.22 ) | 3.56 ( 1.71 ) | 1.83 ( 0.65 ) |
| Invasive breast carcinoma | 5 | 10.3 ( 4.26 - 24.91 ) | 10.3 ( 41.32 ) | 10.15 ( 4.85 ) | 3.34 ( 2.15 ) |
| Malignant melanoma in situ | 5 | 3.35 ( 1.39 - 8.07 ) | 3.35 ( 8.21 ) | 3.34 ( 1.6 ) | 1.74 ( 0.56 ) |
| Enterocolitis haemorrhagic | 5 | 4.47 ( 1.86 - 10.78 ) | 4.47 ( 13.38 ) | 4.45 ( 2.13 ) | 2.15 ( 0.97 ) |
| Incorrect product formulation administered | 5 | 4.89 ( 2.03 - 11.8 ) | 4.89 ( 15.37 ) | 4.86 ( 2.33 ) | 2.28 ( 1.1 ) |
| Bell's palsy | 5 | 2.98 ( 1.24 - 7.18 ) | 2.98 ( 6.55 ) | 2.97 ( 1.42 ) | 1.57 ( 0.39 ) |
| Behcet's syndrome | 5 | 3.62 ( 1.5 - 8.73 ) | 3.62 ( 9.44 ) | 3.61 ( 1.73 ) | 1.85 ( 0.67 ) |
| Haematological infection | 5 | 2.59 ( 1.08 - 6.23 ) | 2.59 ( 4.85 ) | 2.58 ( 1.24 ) | 1.37 ( 0.19 ) |
| Injection site joint warmth | 4 | 435.22 ( 122.81 - 1542.32 ) | 435.18 ( 1039.65 ) | 261.51 ( 90.73 ) | 8.03 ( 6.47 ) |
| Genital candidiasis | 4 | 20.89 ( 7.72 - 56.54 ) | 20.89 ( 73.4 ) | 20.27 ( 8.81 ) | 4.34 ( 3.03 ) |
| Vulvovaginal candidiasis | 4 | 3.54 ( 1.33 - 9.47 ) | 3.54 ( 7.26 ) | 3.53 ( 1.55 ) | 1.82 ( 0.52 ) |
| Granuloma annulare | 4 | 6.73 ( 2.51 - 18.02 ) | 6.73 ( 19.31 ) | 6.67 ( 2.93 ) | 2.74 ( 1.44 ) |
| Cellulitis staphylococcal | 4 | 7.91 ( 2.95 - 21.21 ) | 7.91 ( 23.87 ) | 7.83 ( 3.43 ) | 2.97 ( 1.67 ) |
| Acarodermatitis | 4 | 3.67 ( 1.37 - 9.81 ) | 3.67 ( 7.74 ) | 3.66 ( 1.61 ) | 1.87 ( 0.58 ) |
| Eyelid rash | 4 | 4.02 ( 1.51 - 10.75 ) | 4.02 ( 9.03 ) | 4 ( 1.76 ) | 2 ( 0.71 ) |
| Lipids increased | 4 | 2.65 ( 0.99 - 7.08 ) | 2.65 ( 4.1 ) | 2.64 ( 1.16 ) | 1.4 ( 0.11 ) |
| Tonsillectomy | 4 | 3.41 ( 1.28 - 9.12 ) | 3.41 ( 6.79 ) | 3.4 ( 1.49 ) | 1.77 ( 0.47 ) |
| Breast abscess | 4 | 4.2 ( 1.57 - 11.24 ) | 4.2 ( 9.71 ) | 4.18 ( 1.84 ) | 2.06 ( 0.77 ) |
| Body mass index increased | 4 | 3.11 ( 1.17 - 8.31 ) | 3.11 ( 5.71 ) | 3.1 ( 1.36 ) | 1.63 ( 0.34 ) |
| Abscess oral | 4 | 3.54 ( 1.33 - 9.47 ) | 3.54 ( 7.26 ) | 3.53 ( 1.55 ) | 1.82 ( 0.52 ) |
| Ovarian operation | 4 | 49.27 ( 17.83 - 136.14 ) | 49.27 ( 175.87 ) | 45.88 ( 19.6 ) | 5.52 ( 4.17 ) |
| Angular cheilitis | 4 | 4.85 ( 1.81 - 12.98 ) | 4.85 ( 12.15 ) | 4.82 ( 2.12 ) | 2.27 ( 0.97 ) |
| Injection site hyperaesthesia | 4 | 16.96 ( 6.28 - 45.76 ) | 16.96 ( 58.54 ) | 16.55 ( 7.21 ) | 4.05 ( 2.74 ) |
| Injection site scab | 4 | 5.12 ( 1.91 - 13.7 ) | 5.12 ( 13.16 ) | 5.09 ( 2.23 ) | 2.35 ( 1.05 ) |
| Staphylococcal abscess | 4 | 9.97 ( 3.71 - 26.76 ) | 9.97 ( 31.78 ) | 9.83 ( 4.3 ) | 3.3 ( 1.99 ) |
| Cutaneous lymphoma | 4 | 17.41 ( 6.45 - 46.99 ) | 17.41 ( 60.25 ) | 16.98 ( 7.4 ) | 4.09 ( 2.77 ) |
| Injection site dryness | 4 | 4.57 ( 1.71 - 12.23 ) | 4.57 ( 11.09 ) | 4.55 ( 2 ) | 2.19 ( 0.89 ) |
| Transitional cell carcinoma | 4 | 3.67 ( 1.37 - 9.8 ) | 3.67 ( 7.72 ) | 3.65 ( 1.6 ) | 1.87 ( 0.57 ) |
| Paternal exposure during pregnancy | 4 | 3.93 ( 1.47 - 10.51 ) | 3.93 ( 8.69 ) | 3.91 ( 1.72 ) | 1.97 ( 0.67 ) |
| Hepatic infection | 4 | 3.11 ( 1.17 - 8.31 ) | 3.11 ( 5.71 ) | 3.1 ( 1.36 ) | 1.63 ( 0.34 ) |
| Gingival recession | 4 | 2.79 ( 1.04 - 7.45 ) | 2.79 ( 4.57 ) | 2.78 ( 1.22 ) | 1.48 ( 0.18 ) |
| Pharyngeal abscess | 4 | 7.82 ( 2.92 - 20.95 ) | 7.82 ( 23.5 ) | 7.74 ( 3.39 ) | 2.95 ( 1.65 ) |
| Body tinea | 4 | 9 ( 3.36 - 24.15 ) | 9 ( 28.07 ) | 8.89 ( 3.9 ) | 3.15 ( 1.85 ) |
| Skin striae | 4 | 3.3 ( 1.24 - 8.82 ) | 3.3 ( 6.38 ) | 3.29 ( 1.45 ) | 1.72 ( 0.42 ) |
| Hepatitis a | 4 | 5.89 ( 2.2 - 15.78 ) | 5.89 ( 16.11 ) | 5.85 ( 2.57 ) | 2.55 ( 1.25 ) |
| Paradoxical psoriasis | 4 | 10.45 ( 3.89 - 28.05 ) | 10.44 ( 33.62 ) | 10.3 ( 4.5 ) | 3.36 ( 2.06 ) |
| Blood pressure measurement | 4 | 5.52 ( 2.06 - 14.77 ) | 5.52 ( 14.68 ) | 5.48 ( 2.41 ) | 2.45 ( 1.16 ) |
| Dermo-hypodermitis | 4 | 6.64 ( 2.48 - 17.79 ) | 6.64 ( 18.99 ) | 6.59 ( 2.89 ) | 2.72 ( 1.42 ) |
| Injection site erosion | 3 | 9.94 ( 3.18 - 31.09 ) | 9.94 ( 23.76 ) | 9.81 ( 3.78 ) | 3.29 ( 1.84 ) |
| Serum sickness-like reaction | 3 | 4.96 ( 1.59 - 15.44 ) | 4.96 ( 9.41 ) | 4.93 ( 1.9 ) | 2.3 ( 0.85 ) |
| Oesophageal haemorrhage | 3 | 2.77 ( 0.89 - 8.6 ) | 2.77 ( 3.37 ) | 2.76 ( 1.07 ) | 1.46 ( 0.02 ) |
| Mechanical urticaria | 3 | 6.9 ( 2.21 - 21.51 ) | 6.9 ( 14.96 ) | 6.83 ( 2.64 ) | 2.77 ( 1.32 ) |
| Injection site pustule | 3 | 10.47 ( 3.35 - 32.77 ) | 10.47 ( 25.3 ) | 10.32 ( 3.97 ) | 3.37 ( 1.91 ) |
| Koebner phenomenon | 3 | 36.95 ( 11.55 - 118.25 ) | 36.95 ( 99.31 ) | 35.02 ( 13.23 ) | 5.13 ( 3.64 ) |
| Neutrophilic dermatosis | 3 | 7.45 ( 2.39 - 23.24 ) | 7.45 ( 16.55 ) | 7.37 ( 2.85 ) | 2.88 ( 1.43 ) |
| Leukoplakia oral | 3 | 7.59 ( 2.43 - 23.69 ) | 7.59 ( 16.97 ) | 7.51 ( 2.9 ) | 2.91 ( 1.46 ) |
| Lentigo maligna | 3 | 8.94 ( 2.86 - 27.94 ) | 8.94 ( 20.88 ) | 8.83 ( 3.41 ) | 3.14 ( 1.69 ) |
| Chalazion | 3 | 4.92 ( 1.58 - 15.32 ) | 4.92 ( 9.3 ) | 4.89 ( 1.89 ) | 2.29 ( 0.84 ) |
| Clostridial infection | 3 | 3.5 ( 1.13 - 10.9 ) | 3.5 ( 5.34 ) | 3.49 ( 1.35 ) | 1.8 ( 0.36 ) |
| Lacunar stroke | 3 | 7.56 ( 2.42 - 23.6 ) | 7.56 ( 16.88 ) | 7.49 ( 2.89 ) | 2.9 ( 1.45 ) |
| Intervertebral disc operation | 3 | 3.13 ( 1.01 - 9.74 ) | 3.13 ( 4.34 ) | 3.12 ( 1.21 ) | 1.64 ( 0.2 ) |
| Cyst removal | 3 | 7.31 ( 2.34 - 22.8 ) | 7.31 ( 16.15 ) | 7.24 ( 2.79 ) | 2.86 ( 1.4 ) |
| Manufacturing product storage issue | 3 | 26.47 ( 8.34 - 83.95 ) | 26.46 ( 70.64 ) | 25.47 ( 9.7 ) | 4.67 ( 3.19 ) |
| Angioplasty | 3 | 3.43 ( 1.1 - 10.67 ) | 3.43 ( 5.14 ) | 3.42 ( 1.32 ) | 1.77 ( 0.32 ) |
| Addison's disease | 3 | 2.81 ( 0.9 - 8.72 ) | 2.81 ( 3.47 ) | 2.8 ( 1.08 ) | 1.48 ( 0.04 ) |
| Keratoacanthoma | 3 | 5.53 ( 1.78 - 17.24 ) | 5.53 ( 11.04 ) | 5.49 ( 2.12 ) | 2.46 ( 1.01 ) |
| Cyst rupture | 3 | 5.1 ( 1.64 - 15.88 ) | 5.1 ( 9.81 ) | 5.07 ( 1.96 ) | 2.34 ( 0.89 ) |
| Infected bite | 3 | 2.91 ( 0.94 - 9.05 ) | 2.91 ( 3.74 ) | 2.9 ( 1.12 ) | 1.54 ( 0.09 ) |
| Excessive cerumen production | 3 | 4.01 ( 1.29 - 12.49 ) | 4.01 ( 6.75 ) | 3.99 ( 1.55 ) | 2 ( 0.55 ) |
| Gastric mucosal lesion | 3 | 5.35 ( 1.72 - 16.67 ) | 5.35 ( 10.53 ) | 5.32 ( 2.05 ) | 2.41 ( 0.96 ) |
| Smear cervix abnormal | 3 | 3.59 ( 1.16 - 11.18 ) | 3.59 ( 5.58 ) | 3.58 ( 1.38 ) | 1.84 ( 0.39 ) |
| Gastric operation | 3 | 3.74 ( 1.2 - 11.63 ) | 3.74 ( 5.98 ) | 3.72 ( 1.44 ) | 1.9 ( 0.45 ) |
| Anal cancer | 3 | 3.12 ( 1 - 9.71 ) | 3.12 ( 4.31 ) | 3.11 ( 1.21 ) | 1.64 ( 0.19 ) |
| Injection site ulcer | 3 | 4.53 ( 1.46 - 14.11 ) | 4.53 ( 8.2 ) | 4.51 ( 1.74 ) | 2.17 ( 0.72 ) |
| Eosinophilic colitis | 3 | 9.46 ( 3.03 - 29.58 ) | 9.46 ( 22.37 ) | 9.34 ( 3.6 ) | 3.22 ( 1.77 ) |
| Piloerection | 3 | 3.52 ( 1.13 - 10.94 ) | 3.52 ( 5.37 ) | 3.5 ( 1.36 ) | 1.81 ( 0.36 ) |
| T-cell lymphoma | 3 | 3.67 ( 1.18 - 11.41 ) | 3.67 ( 5.79 ) | 3.65 ( 1.41 ) | 1.87 ( 0.42 ) |
| False positive tuberculosis test | 3 | 27.58 ( 8.69 - 87.58 ) | 27.58 ( 73.74 ) | 26.5 ( 10.08 ) | 4.73 ( 3.25 ) |
| Prostate infection | 3 | 3.56 ( 1.14 - 11.07 ) | 3.56 ( 5.49 ) | 3.55 ( 1.37 ) | 1.83 ( 0.38 ) |
| Mastoiditis | 3 | 3.87 ( 1.24 - 12.04 ) | 3.87 ( 6.35 ) | 3.85 ( 1.49 ) | 1.95 ( 0.5 ) |
| Incorrect product dosage form administered | 3 | 6.82 ( 2.19 - 21.28 ) | 6.82 ( 14.76 ) | 6.76 ( 2.61 ) | 2.76 ( 1.31 ) |
| Pulmonary sarcoidosis | 3 | 3.41 ( 1.1 - 10.61 ) | 3.41 ( 5.09 ) | 3.4 ( 1.32 ) | 1.77 ( 0.32 ) |
| Needle track marks | 3 | 10.64 ( 3.4 - 33.31 ) | 10.64 ( 25.79 ) | 10.49 ( 4.04 ) | 3.39 ( 1.93 ) |
| Injection site coldness | 3 | 5.06 ( 1.62 - 15.76 ) | 5.06 ( 9.7 ) | 5.03 ( 1.94 ) | 2.33 ( 0.88 ) |
| Burns third degree | 3 | 3.7 ( 1.19 - 11.52 ) | 3.7 ( 5.88 ) | 3.69 ( 1.43 ) | 1.88 ( 0.43 ) |
| Chronic disease | 3 | 10.04 ( 3.21 - 31.41 ) | 10.04 ( 24.06 ) | 9.91 ( 3.82 ) | 3.31 ( 1.85 ) |
| Nail bed disorder | 3 | 6.97 ( 2.23 - 21.74 ) | 6.97 ( 15.18 ) | 6.91 ( 2.67 ) | 2.79 ( 1.34 ) |
| Ovarian cancer metastatic | 3 | 5.81 ( 1.86 - 18.11 ) | 5.81 ( 11.84 ) | 5.77 ( 2.23 ) | 2.53 ( 1.08 ) |
| Injection site macule | 3 | 7.74 ( 2.48 - 24.16 ) | 7.74 ( 17.4 ) | 7.66 ( 2.96 ) | 2.94 ( 1.48 ) |
| Axillary mass | 3 | 3.41 ( 1.1 - 10.59 ) | 3.41 ( 5.07 ) | 3.39 ( 1.31 ) | 1.76 ( 0.31 ) |
| Morning sickness | 3 | 4.6 ( 1.48 - 14.31 ) | 4.6 ( 8.38 ) | 4.57 ( 1.77 ) | 2.19 ( 0.74 ) |
| Abnormal cord insertion | 3 | 89.02 ( 26.64 - 297.43 ) | 89.01 ( 229.75 ) | 78.45 ( 28.59 ) | 6.29 ( 4.74 ) |
| Lymphoid tissue hyperplasia | 2 | 9.74 ( 2.41 - 39.36 ) | 9.74 ( 15.46 ) | 9.61 ( 2.99 ) | 3.27 ( 1.58 ) |
| Intertrigo | 2 | 4.14 ( 1.03 - 16.65 ) | 4.14 ( 4.74 ) | 4.12 ( 1.29 ) | 2.04 ( 0.37 ) |
| Injection | 2 | 21.4 ( 5.23 - 87.53 ) | 21.4 ( 37.66 ) | 20.75 ( 6.39 ) | 4.38 ( 2.67 ) |
| Cerumen impaction | 2 | 4.89 ( 1.22 - 19.65 ) | 4.89 ( 6.14 ) | 4.86 ( 1.52 ) | 2.28 ( 0.61 ) |
| Oropharyngeal spasm | 2 | 6.56 ( 1.63 - 26.42 ) | 6.56 ( 9.33 ) | 6.51 ( 2.03 ) | 2.7 ( 1.02 ) |
| Body mass index decreased | 2 | 4.91 ( 1.22 - 19.73 ) | 4.91 ( 6.18 ) | 4.88 ( 1.52 ) | 2.29 ( 0.61 ) |
| Tinea capitis | 2 | 20.72 ( 5.07 - 84.7 ) | 20.72 ( 36.39 ) | 20.12 ( 6.19 ) | 4.33 ( 2.63 ) |
| Epiglottitis | 2 | 4.68 ( 1.16 - 18.8 ) | 4.68 ( 5.75 ) | 4.65 ( 1.45 ) | 2.22 ( 0.54 ) |
| Superficial spreading melanoma stage unspecified | 2 | 9 ( 2.23 - 36.35 ) | 9 ( 14.04 ) | 8.89 ( 2.77 ) | 3.15 ( 1.47 ) |
| Lichen sclerosus | 2 | 3.25 ( 0.81 - 13.03 ) | 3.25 ( 3.1 ) | 3.24 ( 1.01 ) | 1.69 ( 0.02 ) |
| Hypobarism | 2 | 10.88 ( 2.69 - 44.01 ) | 10.88 ( 17.65 ) | 10.72 ( 3.33 ) | 3.42 ( 1.74 ) |
| Arterial stenosis | 2 | 3.33 ( 0.83 - 13.36 ) | 3.33 ( 3.24 ) | 3.32 ( 1.04 ) | 1.73 ( 0.06 ) |
| Cellulitis streptococcal | 2 | 15.54 ( 3.82 - 63.18 ) | 15.54 ( 26.58 ) | 15.2 ( 4.7 ) | 3.93 ( 2.23 ) |
| Breast cancer male | 2 | 3.94 ( 0.98 - 15.84 ) | 3.94 ( 4.37 ) | 3.93 ( 1.23 ) | 1.97 ( 0.3 ) |
| Gingival discolouration | 2 | 5.53 ( 1.38 - 22.25 ) | 5.53 ( 7.36 ) | 5.49 ( 1.71 ) | 2.46 ( 0.78 ) |
| Infective glossitis | 2 | 13.89 ( 3.42 - 56.36 ) | 13.89 ( 23.42 ) | 13.62 ( 4.22 ) | 3.77 ( 2.08 ) |
| Palatal disorder | 2 | 5.33 ( 1.33 - 21.43 ) | 5.33 ( 6.98 ) | 5.29 ( 1.65 ) | 2.4 ( 0.73 ) |
| Animal attack | 2 | 21.4 ( 5.23 - 87.53 ) | 21.4 ( 37.66 ) | 20.75 ( 6.39 ) | 4.38 ( 2.67 ) |
| Groin infection | 2 | 4.03 ( 1 - 16.18 ) | 4.03 ( 4.53 ) | 4.01 ( 1.25 ) | 2 ( 0.33 ) |
| Burning mouth syndrome | 2 | 3.76 ( 0.94 - 15.1 ) | 3.76 ( 4.03 ) | 3.75 ( 1.17 ) | 1.91 ( 0.23 ) |
| Cell marker increased | 2 | 5.33 ( 1.33 - 21.43 ) | 5.33 ( 6.98 ) | 5.29 ( 1.65 ) | 2.4 ( 0.73 ) |
| Dysplastic naevus | 2 | 5.68 ( 1.41 - 22.84 ) | 5.68 ( 7.64 ) | 5.64 ( 1.76 ) | 2.49 ( 0.82 ) |
| Hepatitis alcoholic | 2 | 9 ( 2.23 - 36.35 ) | 9 ( 14.04 ) | 8.89 ( 2.77 ) | 3.15 ( 1.47 ) |
| Respiratory tract inflammation | 2 | 4.61 ( 1.15 - 18.54 ) | 4.61 ( 5.62 ) | 4.59 ( 1.43 ) | 2.2 ( 0.52 ) |
| Hepatitis b core antibody positive | 2 | 5.02 ( 1.25 - 20.19 ) | 5.02 ( 6.39 ) | 4.99 ( 1.56 ) | 2.32 ( 0.64 ) |
| Tinea versicolour | 2 | 5.93 ( 1.47 - 23.88 ) | 5.93 ( 8.13 ) | 5.89 ( 1.84 ) | 2.56 ( 0.88 ) |
| Genital ulceration | 2 | 3.38 ( 0.84 - 13.57 ) | 3.38 ( 3.34 ) | 3.37 ( 1.05 ) | 1.75 ( 0.08 ) |
| Vaginal ulceration | 2 | 5.78 ( 1.44 - 23.24 ) | 5.78 ( 7.83 ) | 5.73 ( 1.79 ) | 2.52 ( 0.84 ) |
| Jaw operation | 2 | 3.5 ( 0.87 - 14.05 ) | 3.5 ( 3.55 ) | 3.49 ( 1.09 ) | 1.8 ( 0.13 ) |
| Endometrial ablation | 2 | 6.25 ( 1.55 - 25.14 ) | 6.25 ( 8.73 ) | 6.2 ( 1.93 ) | 2.63 ( 0.95 ) |
| Hysteroscopy | 2 | 37.3 ( 8.97 - 155.1 ) | 37.3 ( 66.84 ) | 35.34 ( 10.73 ) | 5.14 ( 3.41 ) |
| Hand dermatitis | 2 | 3.54 ( 0.88 - 14.2 ) | 3.54 ( 3.62 ) | 3.52 ( 1.1 ) | 1.82 ( 0.14 ) |
| Oral pustule | 2 | 10.12 ( 2.5 - 40.91 ) | 10.12 ( 16.19 ) | 9.98 ( 3.1 ) | 3.32 ( 1.63 ) |
| Endometriosis ablation | 2 | 76.8 ( 17.74 - 332.43 ) | 76.8 ( 133.87 ) | 68.82 ( 20.2 ) | 6.1 ( 4.32 ) |
| Steroid therapy | 2 | 10.2 ( 2.52 - 41.23 ) | 10.2 ( 16.34 ) | 10.06 ( 3.13 ) | 3.33 ( 1.65 ) |
| Skin graft | 2 | 4.28 ( 1.07 - 17.19 ) | 4.28 ( 5 ) | 4.26 ( 1.33 ) | 2.09 ( 0.42 ) |
| Langerhans' cell histiocytosis | 2 | 5.65 ( 1.41 - 22.74 ) | 5.65 ( 7.59 ) | 5.61 ( 1.75 ) | 2.49 ( 0.81 ) |
| Abdominoplasty | 2 | 8.37 ( 2.07 - 33.76 ) | 8.37 ( 12.81 ) | 8.28 ( 2.58 ) | 3.05 ( 1.37 ) |
| Skin operation | 2 | 6.59 ( 1.64 - 26.55 ) | 6.59 ( 9.4 ) | 6.54 ( 2.04 ) | 2.71 ( 1.03 ) |
| Lung carcinoma cell type unspecified recurrent | 2 | 3.45 ( 0.86 - 13.86 ) | 3.45 ( 3.47 ) | 3.44 ( 1.08 ) | 1.78 ( 0.11 ) |
| Sweat gland infection | 2 | 23.31 ( 5.69 - 95.54 ) | 23.31 ( 41.24 ) | 22.54 ( 6.93 ) | 4.49 ( 2.79 ) |
| Interferon gamma release assay positive | 2 | 9.82 ( 2.43 - 39.66 ) | 9.82 ( 15.6 ) | 9.69 ( 3.01 ) | 3.28 ( 1.59 ) |
| Hiv test positive | 2 | 4.4 ( 1.09 - 17.66 ) | 4.4 ( 5.21 ) | 4.37 ( 1.37 ) | 2.13 ( 0.45 ) |
| Hepatitis c virus test positive | 2 | 3.97 ( 0.99 - 15.93 ) | 3.97 ( 4.41 ) | 3.95 ( 1.23 ) | 1.98 ( 0.31 ) |
| Self-consciousness | 2 | 5.91 ( 1.47 - 23.77 ) | 5.91 ( 8.08 ) | 5.86 ( 1.83 ) | 2.55 ( 0.87 ) |
| Viral pharyngitis | 2 | 5.58 ( 1.39 - 22.44 ) | 5.58 ( 7.45 ) | 5.54 ( 1.73 ) | 2.47 ( 0.79 ) |
| Cutaneous sarcoidosis | 2 | 5.75 ( 1.43 - 23.14 ) | 5.75 ( 7.78 ) | 5.71 ( 1.78 ) | 2.51 ( 0.84 ) |
| Pneumococcal sepsis | 2 | 3.52 ( 0.88 - 14.12 ) | 3.52 ( 3.59 ) | 3.51 ( 1.1 ) | 1.81 ( 0.14 ) |
| Fallopian tube disorder | 2 | 10.12 ( 2.5 - 40.91 ) | 10.12 ( 16.19 ) | 9.98 ( 3.1 ) | 3.32 ( 1.63 ) |
| Triple negative breast cancer | 2 | 4.03 ( 1 - 16.18 ) | 4.03 ( 4.53 ) | 4.01 ( 1.25 ) | 2 ( 0.33 ) |
| Keratosis pilaris | 2 | 8.59 ( 2.13 - 34.66 ) | 8.59 ( 13.24 ) | 8.49 ( 2.64 ) | 3.09 ( 1.4 ) |
| Parotid gland enlargement | 2 | 4.53 ( 1.13 - 18.21 ) | 4.53 ( 5.47 ) | 4.51 ( 1.41 ) | 2.17 ( 0.5 ) |
| Upper respiratory fungal infection | 2 | 10.88 ( 2.69 - 44.01 ) | 10.88 ( 17.65 ) | 10.72 ( 3.33 ) | 3.42 ( 1.74 ) |
| Genital infection | 2 | 6.07 ( 1.51 - 24.44 ) | 6.07 ( 8.4 ) | 6.03 ( 1.88 ) | 2.59 ( 0.91 ) |
| Pleuropericarditis | 2 | 6.56 ( 1.63 - 26.42 ) | 6.56 ( 9.33 ) | 6.51 ( 2.03 ) | 2.7 ( 1.02 ) |
| Chronic tonsillitis | 2 | 16.53 ( 4.06 - 67.25 ) | 16.53 ( 28.45 ) | 16.14 ( 4.99 ) | 4.01 ( 2.32 ) |
| Infection parasitic | 2 | 3.22 ( 0.8 - 12.93 ) | 3.22 ( 3.05 ) | 3.21 ( 1 ) | 1.68 ( 0.01 ) |
| Helminthic infection | 2 | 8.01 ( 1.99 - 32.3 ) | 8.01 ( 12.12 ) | 7.92 ( 2.47 ) | 2.99 ( 1.31 ) |
| Sapho syndrome | 2 | 7.68 ( 1.91 - 30.96 ) | 7.68 ( 11.48 ) | 7.6 ( 2.37 ) | 2.93 ( 1.25 ) |
| Transient global amnesia | 2 | 3.47 ( 0.87 - 13.94 ) | 3.47 ( 3.5 ) | 3.46 ( 1.08 ) | 1.79 ( 0.12 ) |
| Biopsy lymph gland | 2 | 27.2 ( 6.61 - 111.92 ) | 27.2 ( 48.45 ) | 26.15 ( 8.01 ) | 4.71 ( 2.99 ) |
| Thymoma | 2 | 6.63 ( 1.65 - 26.69 ) | 6.63 ( 9.46 ) | 6.57 ( 2.05 ) | 2.72 ( 1.04 ) |
| Abscess neck | 2 | 3.32 ( 0.83 - 13.33 ) | 3.32 ( 3.23 ) | 3.31 ( 1.03 ) | 1.73 ( 0.05 ) |
| Morphoea | 2 | 4.96 ( 1.23 - 19.95 ) | 4.96 ( 6.28 ) | 4.93 ( 1.54 ) | 2.3 ( 0.63 ) |
| Wrong patient | 2 | 4.5 ( 1.12 - 18.09 ) | 4.5 ( 5.41 ) | 4.48 ( 1.4 ) | 2.16 ( 0.49 ) |
| Eczema nummular | 2 | 6.66 ( 1.65 - 26.82 ) | 6.66 ( 9.53 ) | 6.6 ( 2.06 ) | 2.72 ( 1.04 ) |
| Nail dystrophy | 2 | 3.71 ( 0.92 - 14.89 ) | 3.71 ( 3.93 ) | 3.69 ( 1.15 ) | 1.89 ( 0.21 ) |
| Cervicobrachial syndrome | 2 | 5.56 ( 1.38 - 22.35 ) | 5.56 ( 7.41 ) | 5.52 ( 1.72 ) | 2.46 ( 0.79 ) |
| Foetal growth abnormality | 2 | 9.82 ( 2.43 - 39.66 ) | 9.82 ( 15.6 ) | 9.69 ( 3.01 ) | 3.28 ( 1.59 ) |
| Reaction to colouring | 2 | 6.28 ( 1.56 - 25.27 ) | 6.28 ( 8.79 ) | 6.23 ( 1.94 ) | 2.64 ( 0.96 ) |
| Rectal adenocarcinoma | 2 | 3.41 ( 0.85 - 13.68 ) | 3.41 ( 3.39 ) | 3.4 ( 1.06 ) | 1.76 ( 0.09 ) |
| Elbow operation | 2 | 3.31 ( 0.83 - 13.3 ) | 3.31 ( 3.21 ) | 3.3 ( 1.03 ) | 1.72 ( 0.05 ) |
| Uterine cyst | 2 | 4.53 ( 1.13 - 18.21 ) | 4.53 ( 5.47 ) | 4.51 ( 1.41 ) | 2.17 ( 0.5 ) |
| Renal cancer metastatic | 2 | 3.76 ( 0.94 - 15.1 ) | 3.76 ( 4.03 ) | 3.75 ( 1.17 ) | 1.91 ( 0.23 ) |
| Ischaemic skin ulcer | 2 | 16.96 ( 4.17 - 69.02 ) | 16.96 ( 29.27 ) | 16.55 ( 5.11 ) | 4.05 ( 2.35 ) |
| Carbon dioxide abnormal | 2 | 6.91 ( 1.71 - 27.82 ) | 6.91 ( 10 ) | 6.85 ( 2.13 ) | 2.78 ( 1.1 ) |
| Malacoplakia | 2 | 6.04 ( 1.5 - 24.32 ) | 6.04 ( 8.34 ) | 6 ( 1.87 ) | 2.58 ( 0.91 ) |
| Peripheral artery bypass | 2 | 17.64 ( 4.33 - 71.87 ) | 17.64 ( 30.57 ) | 17.2 ( 5.31 ) | 4.1 ( 2.41 ) |
| Renal aplasia | 2 | 3.44 ( 0.86 - 13.79 ) | 3.44 ( 3.44 ) | 3.42 ( 1.07 ) | 1.78 ( 0.1 ) |
| Rectosigmoid cancer | 2 | 11.55 ( 2.85 - 46.77 ) | 11.55 ( 18.94 ) | 11.37 ( 3.53 ) | 3.51 ( 1.82 ) |
| Foetal exposure via father | 2 | 100.43 ( 22.66 - 445.06 ) | 100.43 ( 170.62 ) | 87.17 ( 25.08 ) | 6.45 ( 4.63 ) |
| Purpura non-thrombocytopenic | 1 | 16.74 ( 2.3 - 121.84 ) | 16.74 ( 14.43 ) | 16.34 ( 3.1 ) | 4.03 ( 1.94 ) |
| Exposure to contaminated device | 1 | 6.59 ( 0.92 - 47.28 ) | 6.59 ( 4.7 ) | 6.54 ( 1.26 ) | 2.71 ( 0.65 ) |
| Binge drinking | 1 | 11.06 ( 1.53 - 79.86 ) | 11.06 ( 9 ) | 10.9 ( 2.08 ) | 3.45 ( 1.37 ) |
| Pharyngeal neoplasm | 1 | 7.68 ( 1.07 - 55.15 ) | 7.68 ( 5.74 ) | 7.6 ( 1.46 ) | 2.93 ( 0.86 ) |
| Laryngeal neoplasm | 1 | 8.59 ( 1.19 - 61.77 ) | 8.59 ( 6.62 ) | 8.49 ( 1.63 ) | 3.09 ( 1.02 ) |
| Oesophageal oedema | 1 | 5.22 ( 0.73 - 37.37 ) | 5.22 ( 3.39 ) | 5.19 ( 1 ) | 2.38 ( 0.32 ) |
| Injection site pallor | 1 | 4.76 ( 0.67 - 34.07 ) | 4.76 ( 2.95 ) | 4.74 ( 0.91 ) | 2.24 ( 0.19 ) |
| Parapsoriasis | 1 | 4.84 ( 0.68 - 34.58 ) | 4.84 ( 3.02 ) | 4.81 ( 0.93 ) | 2.27 ( 0.21 ) |
| Albumin globulin ratio abnormal | 1 | 31.09 ( 4.18 - 231.1 ) | 31.08 ( 27.79 ) | 29.72 ( 5.55 ) | 4.89 ( 2.77 ) |
| Injection site dermatitis | 1 | 10.88 ( 1.51 - 78.51 ) | 10.88 ( 8.82 ) | 10.72 ( 2.05 ) | 3.42 ( 1.35 ) |
| Urogenital disorder | 1 | 6.1 ( 0.85 - 43.71 ) | 6.1 ( 4.23 ) | 6.05 ( 1.17 ) | 2.6 ( 0.54 ) |
| Blood ethanol increased | 1 | 10.88 ( 1.51 - 78.51 ) | 10.88 ( 8.82 ) | 10.72 ( 2.05 ) | 3.42 ( 1.35 ) |
| Injection site eczema | 1 | 5.83 ( 0.81 - 41.74 ) | 5.83 ( 3.96 ) | 5.79 ( 1.11 ) | 2.53 ( 0.47 ) |
| Breast inflammation | 1 | 4.21 ( 0.59 - 30.09 ) | 4.21 ( 2.43 ) | 4.19 ( 0.81 ) | 2.07 ( 0.01 ) |
| Histone antibody positive | 1 | 8.82 ( 1.23 - 63.46 ) | 8.82 ( 6.84 ) | 8.72 ( 1.67 ) | 3.12 ( 1.06 ) |
| Culture wound positive | 1 | 13.6 ( 1.88 - 98.53 ) | 13.6 ( 11.43 ) | 13.34 ( 2.54 ) | 3.74 ( 1.66 ) |
| Adenoviral conjunctivitis | 1 | 50.21 ( 6.57 - 383.87 ) | 50.21 ( 44.79 ) | 46.7 ( 8.51 ) | 5.55 ( 3.37 ) |
| Renal cell carcinoma stage i | 1 | 12.8 ( 1.77 - 92.63 ) | 12.8 ( 10.67 ) | 12.57 ( 2.4 ) | 3.65 ( 1.57 ) |
| Nodular melanoma | 1 | 5.53 ( 0.77 - 39.6 ) | 5.53 ( 3.68 ) | 5.49 ( 1.06 ) | 2.46 ( 0.4 ) |
| Eye infection staphylococcal | 1 | 4.6 ( 0.64 - 32.86 ) | 4.6 ( 2.79 ) | 4.57 ( 0.88 ) | 2.19 ( 0.14 ) |
| Hyperproteinaemia | 1 | 4.98 ( 0.7 - 35.64 ) | 4.98 ( 3.16 ) | 4.95 ( 0.95 ) | 2.31 ( 0.25 ) |
| Testicular germ cell cancer | 1 | 59.34 ( 7.66 - 459.68 ) | 59.34 ( 52.58 ) | 54.48 ( 9.82 ) | 5.77 ( 3.58 ) |
| Hepatitis b dna assay positive | 1 | 8.7 ( 1.21 - 62.6 ) | 8.7 ( 6.73 ) | 8.6 ( 1.65 ) | 3.1 ( 1.04 ) |
| Hair follicle tumour benign | 1 | 8.94 ( 1.24 - 64.34 ) | 8.94 ( 6.96 ) | 8.83 ( 1.69 ) | 3.14 ( 1.08 ) |
| Urine bilirubin increased | 1 | 22.51 ( 3.07 - 165.25 ) | 22.51 ( 19.87 ) | 21.79 ( 4.11 ) | 4.45 ( 2.34 ) |
| Influenza b virus test positive | 1 | 5.22 ( 0.73 - 37.37 ) | 5.22 ( 3.39 ) | 5.19 ( 1 ) | 2.38 ( 0.32 ) |
| Larynx irritation | 1 | 4.76 ( 0.67 - 34.07 ) | 4.76 ( 2.95 ) | 4.74 ( 0.91 ) | 2.24 ( 0.19 ) |
| Hypergeusia | 1 | 20.4 ( 2.79 - 149.29 ) | 20.4 ( 17.89 ) | 19.81 ( 3.75 ) | 4.31 ( 2.21 ) |
| Ocular rosacea | 1 | 5.44 ( 0.76 - 38.94 ) | 5.44 ( 3.59 ) | 5.4 ( 1.04 ) | 2.43 ( 0.38 ) |
| Large intestine operation | 1 | 6.59 ( 0.92 - 47.28 ) | 6.59 ( 4.7 ) | 6.54 ( 1.26 ) | 2.71 ( 0.65 ) |
| Administration site rash | 1 | 6.8 ( 0.95 - 48.77 ) | 6.8 ( 4.9 ) | 6.74 ( 1.3 ) | 2.75 ( 0.69 ) |
| Injection site joint pain | 1 | 10.88 ( 1.51 - 78.51 ) | 10.88 ( 8.82 ) | 10.72 ( 2.05 ) | 3.42 ( 1.35 ) |
| Vasectomy | 1 | 5.83 ( 0.81 - 41.74 ) | 5.83 ( 3.96 ) | 5.79 ( 1.11 ) | 2.53 ( 0.47 ) |
| Pulmonary function test | 1 | 16.74 ( 2.3 - 121.84 ) | 16.74 ( 14.43 ) | 16.34 ( 3.1 ) | 4.03 ( 1.94 ) |
| Death of relative | 1 | 4.63 ( 0.65 - 33.1 ) | 4.63 ( 2.83 ) | 4.6 ( 0.89 ) | 2.2 ( 0.15 ) |
| Tonsillitis streptococcal | 1 | 6.87 ( 0.96 - 49.29 ) | 6.87 ( 4.96 ) | 6.81 ( 1.31 ) | 2.77 ( 0.71 ) |
| Metamyelocyte count | 1 | 163.2 ( 18.24 - 1460.19 ) | 163.19 ( 128.96 ) | 130.75 ( 20.9 ) | 7.03 ( 4.67 ) |
| Gallbladder abscess | 1 | 15.54 ( 2.14 - 112.93 ) | 15.54 ( 13.29 ) | 15.2 ( 2.89 ) | 3.93 ( 1.84 ) |
| Injection site hypertrichosis | 1 | 652.79 ( 40.83 - 10437.1 ) | 652.77 ( 325.39 ) | 326.89 ( 32.15 ) | 8.35 ( 5.72 ) |
| Meniscus removal | 1 | 11.66 ( 1.61 - 84.21 ) | 11.66 ( 9.57 ) | 11.47 ( 2.19 ) | 3.52 ( 1.44 ) |
| Influenza immunisation | 1 | 10.2 ( 1.41 - 73.53 ) | 10.2 ( 8.17 ) | 10.06 ( 1.93 ) | 3.33 ( 1.26 ) |
| Salivary gland neoplasm | 1 | 4.32 ( 0.6 - 30.89 ) | 4.32 ( 2.54 ) | 4.3 ( 0.83 ) | 2.1 ( 0.05 ) |
| Antiinflammatory therapy | 1 | 326.39 ( 29.59 - 3599.74 ) | 326.39 ( 216.26 ) | 217.92 ( 29.24 ) | 7.77 ( 5.27 ) |
| Abnormal labour | 1 | 15.54 ( 2.14 - 112.93 ) | 15.54 ( 13.29 ) | 15.2 ( 2.89 ) | 3.93 ( 1.84 ) |
| Carcinoma in situ | 1 | 8.26 ( 1.15 - 59.39 ) | 8.26 ( 6.3 ) | 8.17 ( 1.57 ) | 3.03 ( 0.97 ) |
| Cold exposure injury | 1 | 130.56 ( 15.25 - 1117.56 ) | 130.55 ( 107.14 ) | 108.96 ( 18.07 ) | 6.77 ( 4.45 ) |
| External ear pain | 1 | 10.36 ( 1.44 - 74.71 ) | 10.36 ( 8.33 ) | 10.22 ( 1.96 ) | 3.35 ( 1.28 ) |
| Eosinophilic cellulitis | 1 | 5.18 ( 0.72 - 37.07 ) | 5.18 ( 3.35 ) | 5.15 ( 0.99 ) | 2.36 ( 0.31 ) |
| Biopsy cervix | 1 | 27.2 ( 3.68 - 201.07 ) | 27.2 ( 24.23 ) | 26.15 ( 4.9 ) | 4.71 ( 2.59 ) |
| Viral pericarditis | 1 | 7.96 ( 1.11 - 57.19 ) | 7.96 ( 6.01 ) | 7.88 ( 1.51 ) | 2.98 ( 0.91 ) |
| Retinal operation | 1 | 5.14 ( 0.72 - 36.77 ) | 5.14 ( 3.31 ) | 5.11 ( 0.98 ) | 2.35 ( 0.3 ) |
| Periorbital disorder | 1 | 6.16 ( 0.86 - 44.13 ) | 6.16 ( 4.28 ) | 6.11 ( 1.18 ) | 2.61 ( 0.55 ) |
| Community acquired infection | 1 | 26.11 ( 3.54 - 192.71 ) | 26.11 ( 23.22 ) | 25.15 ( 4.72 ) | 4.65 ( 2.54 ) |
| Planning to become pregnant | 1 | 46.63 ( 6.13 - 354.6 ) | 46.63 ( 41.67 ) | 43.58 ( 7.98 ) | 5.45 ( 3.28 ) |
| Mucosal infection | 1 | 4.84 ( 0.68 - 34.58 ) | 4.84 ( 3.02 ) | 4.81 ( 0.93 ) | 2.27 ( 0.21 ) |
| Pitted keratolysis | 1 | 163.2 ( 18.24 - 1460.19 ) | 163.19 ( 128.96 ) | 130.75 ( 20.9 ) | 7.03 ( 4.67 ) |
| Splinter | 1 | 5.44 ( 0.76 - 38.94 ) | 5.44 ( 3.59 ) | 5.4 ( 1.04 ) | 2.43 ( 0.38 ) |
| Fracture infection | 1 | 12.09 ( 1.67 - 87.39 ) | 12.09 ( 9.99 ) | 11.89 ( 2.27 ) | 3.57 ( 1.49 ) |
| Pharyngotonsillitis | 1 | 4.84 ( 0.68 - 34.58 ) | 4.84 ( 3.02 ) | 4.81 ( 0.93 ) | 2.27 ( 0.21 ) |
| Zinc deficiency | 1 | 5.26 ( 0.74 - 37.67 ) | 5.26 ( 3.43 ) | 5.23 ( 1.01 ) | 2.39 ( 0.33 ) |
| Infection prophylaxis | 1 | 36.27 ( 4.84 - 271.67 ) | 36.27 ( 32.49 ) | 34.41 ( 6.38 ) | 5.1 ( 2.96 ) |
| Vagal nerve stimulator implantation | 1 | 4.84 ( 0.68 - 34.58 ) | 4.84 ( 3.02 ) | 4.81 ( 0.93 ) | 2.27 ( 0.21 ) |
| Pyoderma streptococcal | 1 | 38.4 ( 5.11 - 288.55 ) | 38.4 ( 34.4 ) | 36.32 ( 6.72 ) | 5.18 ( 3.04 ) |
| Biliary cyst | 1 | 9.33 ( 1.3 - 67.14 ) | 9.33 ( 7.33 ) | 9.21 ( 1.77 ) | 3.2 ( 1.13 ) |
| X-ray limb | 1 | 326.39 ( 29.59 - 3599.74 ) | 326.39 ( 216.26 ) | 217.92 ( 29.24 ) | 7.77 ( 5.27 ) |
| Cholelithotomy | 1 | 5.73 ( 0.8 - 41 ) | 5.73 ( 3.87 ) | 5.68 ( 1.09 ) | 2.51 ( 0.45 ) |
| Brucellosis | 1 | 8.48 ( 1.18 - 60.96 ) | 8.48 ( 6.51 ) | 8.38 ( 1.61 ) | 3.07 ( 1 ) |
| Ear infection viral | 1 | 7.02 ( 0.98 - 50.36 ) | 7.02 ( 5.11 ) | 6.96 ( 1.34 ) | 2.8 ( 0.74 ) |
| Non-24-hour sleep-wake disorder | 1 | 5.35 ( 0.75 - 38.29 ) | 5.35 ( 3.51 ) | 5.32 ( 1.02 ) | 2.41 ( 0.35 ) |
| Injection site lymphadenopathy | 1 | 43.52 ( 5.75 - 329.47 ) | 43.52 ( 38.94 ) | 40.86 ( 7.51 ) | 5.35 ( 3.2 ) |
| Pancreatic enzyme abnormality | 1 | 10.53 ( 1.46 - 75.93 ) | 10.53 ( 8.49 ) | 10.38 ( 1.99 ) | 3.38 ( 1.3 ) |
| Pharyngeal cancer | 1 | 5.39 ( 0.75 - 38.61 ) | 5.39 ( 3.55 ) | 5.36 ( 1.03 ) | 2.42 ( 0.37 ) |
| Algophobia | 1 | 130.56 ( 15.25 - 1117.56 ) | 130.55 ( 107.14 ) | 108.96 ( 18.07 ) | 6.77 ( 4.45 ) |
| Laboratory test | 1 | 7.33 ( 1.02 - 52.65 ) | 7.33 ( 5.41 ) | 7.26 ( 1.4 ) | 2.86 ( 0.8 ) |
| Blood creatinine | 1 | 7.25 ( 1.01 - 52.06 ) | 7.25 ( 5.33 ) | 7.18 ( 1.38 ) | 2.84 ( 0.78 ) |
| Laser therapy | 1 | 6.16 ( 0.86 - 44.13 ) | 6.16 ( 4.28 ) | 6.11 ( 1.18 ) | 2.61 ( 0.55 ) |
| Cardiac pacemaker evaluation | 1 | 54.4 ( 7.07 - 418.38 ) | 54.4 ( 48.38 ) | 50.29 ( 9.12 ) | 5.65 ( 3.47 ) |
| Eyelash changes | 1 | 4.73 ( 0.66 - 33.82 ) | 4.73 ( 2.92 ) | 4.7 ( 0.91 ) | 2.23 ( 0.18 ) |
| Pharyngolaryngeal abscess | 1 | 59.34 ( 7.66 - 459.68 ) | 59.34 ( 52.58 ) | 54.48 ( 9.82 ) | 5.77 ( 3.58 ) |
| Liposuction | 1 | 8.7 ( 1.21 - 62.6 ) | 8.7 ( 6.73 ) | 8.6 ( 1.65 ) | 3.1 ( 1.04 ) |
| Limb girth increased | 1 | 93.26 ( 11.47 - 758 ) | 93.25 ( 79.86 ) | 81.72 ( 14.16 ) | 6.35 ( 4.1 ) |
| Facial operation | 1 | 7.96 ( 1.11 - 57.19 ) | 7.96 ( 6.01 ) | 7.88 ( 1.51 ) | 2.98 ( 0.91 ) |
| Conjunctival irritation | 1 | 9.89 ( 1.37 - 71.26 ) | 9.89 ( 7.87 ) | 9.76 ( 1.87 ) | 3.29 ( 1.22 ) |
| Breath sounds normal | 1 | 217.6 ( 22.63 - 2091.98 ) | 217.59 ( 161.7 ) | 163.44 ( 24.6 ) | 7.35 ( 4.94 ) |
| Hypertrophy of tongue papillae | 1 | 9.89 ( 1.37 - 71.26 ) | 9.89 ( 7.87 ) | 9.76 ( 1.87 ) | 3.29 ( 1.22 ) |
| Ear canal stenosis | 1 | 19.78 ( 2.71 - 144.64 ) | 19.78 ( 17.31 ) | 19.23 ( 3.64 ) | 4.27 ( 2.17 ) |
| Vulvar erosion | 1 | 8.26 ( 1.15 - 59.39 ) | 8.26 ( 6.3 ) | 8.17 ( 1.57 ) | 3.03 ( 0.97 ) |
| Injection site streaking | 1 | 6.8 ( 0.95 - 48.77 ) | 6.8 ( 4.9 ) | 6.74 ( 1.3 ) | 2.75 ( 0.69 ) |
| Wrong dosage formulation | 1 | 6.4 ( 0.89 - 45.87 ) | 6.4 ( 4.51 ) | 6.35 ( 1.22 ) | 2.67 ( 0.61 ) |
| Staphylococcal osteomyelitis | 1 | 7.5 ( 1.05 - 53.87 ) | 7.5 ( 5.57 ) | 7.43 ( 1.43 ) | 2.89 ( 0.83 ) |
| Fistula repair | 1 | 6.66 ( 0.93 - 47.76 ) | 6.66 ( 4.76 ) | 6.6 ( 1.27 ) | 2.72 ( 0.66 ) |
| Injection site vasculitis | 1 | 31.09 ( 4.18 - 231.1 ) | 31.08 ( 27.79 ) | 29.72 ( 5.55 ) | 4.89 ( 2.77 ) |
| Coxsackie myocarditis | 1 | 72.53 ( 9.19 - 572.53 ) | 72.53 ( 63.49 ) | 65.38 ( 11.61 ) | 6.03 ( 3.81 ) |
| Foreign travel | 1 | 108.8 ( 13.1 - 903.75 ) | 108.8 ( 91.55 ) | 93.4 ( 15.89 ) | 6.55 ( 4.26 ) |
| Dientamoeba infection | 1 | 59.34 ( 7.66 - 459.68 ) | 59.34 ( 52.58 ) | 54.48 ( 9.82 ) | 5.77 ( 3.58 ) |
| Malignant neoplasm of lacrimal gland | 1 | 217.6 ( 22.63 - 2091.98 ) | 217.59 ( 161.7 ) | 163.44 ( 24.6 ) | 7.35 ( 4.94 ) |
| Colonic haematoma | 1 | 21.06 ( 2.87 - 154.26 ) | 21.06 ( 18.51 ) | 20.43 ( 3.86 ) | 4.35 ( 2.25 ) |
| Squamous cell carcinoma antigen increased | 1 | 130.56 ( 15.25 - 1117.56 ) | 130.55 ( 107.14 ) | 108.96 ( 18.07 ) | 6.77 ( 4.45 ) |
| Removal of internal fixation | 1 | 18.65 ( 2.56 - 136.15 ) | 18.65 ( 16.24 ) | 18.16 ( 3.44 ) | 4.18 ( 2.09 ) |
| Urticaria papular | 1 | 6.53 ( 0.91 - 46.8 ) | 6.53 ( 4.63 ) | 6.47 ( 1.25 ) | 2.69 ( 0.63 ) |
| Tongue neoplasm benign | 1 | 36.27 ( 4.84 - 271.67 ) | 36.27 ( 32.49 ) | 34.41 ( 6.38 ) | 5.1 ( 2.96 ) |
| Angiocardiogram | 1 | 12.09 ( 1.67 - 87.39 ) | 12.09 ( 9.99 ) | 11.89 ( 2.27 ) | 3.57 ( 1.49 ) |
| Semen viscosity decreased | 1 | 10.7 ( 1.48 - 77.2 ) | 10.7 ( 8.65 ) | 10.54 ( 2.02 ) | 3.4 ( 1.33 ) |
| Xanthopsia | 1 | 4.38 ( 0.61 - 31.31 ) | 4.38 ( 2.59 ) | 4.36 ( 0.84 ) | 2.12 ( 0.07 ) |
| Breast disorder female | 1 | 5.14 ( 0.72 - 36.77 ) | 5.14 ( 3.31 ) | 5.11 ( 0.98 ) | 2.35 ( 0.3 ) |
| Bone fistula | 1 | 11.66 ( 1.61 - 84.21 ) | 11.66 ( 9.57 ) | 11.47 ( 2.19 ) | 3.52 ( 1.44 ) |
| Congenital heart valve disorder | 1 | 8.16 ( 1.14 - 58.64 ) | 8.16 ( 6.2 ) | 8.07 ( 1.55 ) | 3.01 ( 0.95 ) |
| Androgenetic alopecia | 1 | 4.29 ( 0.6 - 30.69 ) | 4.29 ( 2.51 ) | 4.27 ( 0.82 ) | 2.1 ( 0.04 ) |
| Viral test negative | 1 | 81.6 ( 10.21 - 652.44 ) | 81.6 ( 70.76 ) | 72.64 ( 12.76 ) | 6.18 ( 3.95 ) |
| Proctitis haemorrhagic | 1 | 14.84 ( 2.04 - 107.69 ) | 14.84 ( 12.62 ) | 14.53 ( 2.77 ) | 3.86 ( 1.78 ) |
| Refusal of treatment by relative | 1 | 9.19 ( 1.28 - 66.18 ) | 9.19 ( 7.2 ) | 9.08 ( 1.74 ) | 3.18 ( 1.11 ) |
| Colitis erosive | 1 | 8.94 ( 1.24 - 64.34 ) | 8.94 ( 6.96 ) | 8.83 ( 1.69 ) | 3.14 ( 1.08 ) |
| Herpes zoster meningoradiculitis | 1 | 93.26 ( 11.47 - 758 ) | 93.25 ( 79.86 ) | 81.72 ( 14.16 ) | 6.35 ( 4.1 ) |
| Her2 negative breast cancer | 1 | 21.76 ( 2.97 - 159.57 ) | 21.76 ( 19.17 ) | 21.09 ( 3.98 ) | 4.4 ( 2.3 ) |
| Malignant sweat gland neoplasm | 1 | 15.54 ( 2.14 - 112.93 ) | 15.54 ( 13.29 ) | 15.2 ( 2.89 ) | 3.93 ( 1.84 ) |
| Silicosis | 1 | 16.74 ( 2.3 - 121.84 ) | 16.74 ( 14.43 ) | 16.34 ( 3.1 ) | 4.03 ( 1.94 ) |
| Hookworm infection | 1 | 34.36 ( 4.6 - 256.66 ) | 34.36 ( 30.77 ) | 32.69 ( 6.08 ) | 5.03 ( 2.9 ) |
| Prostatic adenoma | 1 | 6.87 ( 0.96 - 49.29 ) | 6.87 ( 4.96 ) | 6.81 ( 1.31 ) | 2.77 ( 0.71 ) |
| Thoracic operation | 1 | 6.87 ( 0.96 - 49.29 ) | 6.87 ( 4.96 ) | 6.81 ( 1.31 ) | 2.77 ( 0.71 ) |
| Retroperitoneal abscess | 1 | 4.66 ( 0.65 - 33.34 ) | 4.66 ( 2.86 ) | 4.64 ( 0.89 ) | 2.21 ( 0.16 ) |
| Haemangioma of bone | 1 | 6.22 ( 0.87 - 44.55 ) | 6.22 ( 4.34 ) | 6.17 ( 1.19 ) | 2.62 ( 0.57 ) |
| Lice infestation | 1 | 6.66 ( 0.93 - 47.76 ) | 6.66 ( 4.76 ) | 6.6 ( 1.27 ) | 2.72 ( 0.66 ) |
| Ligamentitis | 1 | 13.32 ( 1.84 - 96.48 ) | 13.32 ( 11.17 ) | 13.08 ( 2.49 ) | 3.71 ( 1.63 ) |
| Laryngitis fungal | 1 | 7.33 ( 1.02 - 52.65 ) | 7.33 ( 5.41 ) | 7.26 ( 1.4 ) | 2.86 ( 0.8 ) |
| Peripheral nerve operation | 1 | 6.1 ( 0.85 - 43.71 ) | 6.1 ( 4.23 ) | 6.05 ( 1.17 ) | 2.6 ( 0.54 ) |
| Vessel perforation | 1 | 11.45 ( 1.59 - 82.71 ) | 11.45 ( 9.38 ) | 11.27 ( 2.16 ) | 3.49 ( 1.42 ) |
| Angiogram peripheral abnormal | 1 | 217.6 ( 22.63 - 2091.98 ) | 217.59 ( 161.7 ) | 163.44 ( 24.6 ) | 7.35 ( 4.94 ) |
| Cystic lung disease | 1 | 5.39 ( 0.75 - 38.61 ) | 5.39 ( 3.55 ) | 5.36 ( 1.03 ) | 2.42 ( 0.37 ) |
| Vaginal lesion | 1 | 4.44 ( 0.62 - 31.74 ) | 4.44 ( 2.65 ) | 4.42 ( 0.85 ) | 2.14 ( 0.09 ) |
| Pericardial drainage | 1 | 4.38 ( 0.61 - 31.31 ) | 4.38 ( 2.59 ) | 4.36 ( 0.84 ) | 2.12 ( 0.07 ) |
| T-lymphocyte count abnormal | 1 | 11.45 ( 1.59 - 82.71 ) | 11.45 ( 9.38 ) | 11.27 ( 2.16 ) | 3.49 ( 1.42 ) |
| Cutaneous t-cell lymphoma stage ii | 1 | 50.21 ( 6.57 - 383.87 ) | 50.21 ( 44.79 ) | 46.7 ( 8.51 ) | 5.55 ( 3.37 ) |
| Cardiac sarcoidosis | 1 | 4.8 ( 0.67 - 34.32 ) | 4.8 ( 2.99 ) | 4.77 ( 0.92 ) | 2.25 ( 0.2 ) |
| Cardiac valve sclerosis | 1 | 16.74 ( 2.3 - 121.84 ) | 16.74 ( 14.43 ) | 16.34 ( 3.1 ) | 4.03 ( 1.94 ) |
| Coxsackie virus test positive | 1 | 72.53 ( 9.19 - 572.53 ) | 72.53 ( 63.49 ) | 65.38 ( 11.61 ) | 6.03 ( 3.81 ) |
| Culture stool positive | 1 | 12.8 ( 1.77 - 92.63 ) | 12.8 ( 10.67 ) | 12.57 ( 2.4 ) | 3.65 ( 1.57 ) |
| Diabetic gastroparesis | 1 | 5.26 ( 0.74 - 37.67 ) | 5.26 ( 3.43 ) | 5.23 ( 1.01 ) | 2.39 ( 0.33 ) |
| Muscle tension dysphonia | 1 | 10.7 ( 1.48 - 77.2 ) | 10.7 ( 8.65 ) | 10.54 ( 2.02 ) | 3.4 ( 1.33 ) |
| Vocal cord thickening | 1 | 4.35 ( 0.61 - 31.1 ) | 4.35 ( 2.56 ) | 4.33 ( 0.84 ) | 2.11 ( 0.06 ) |
| Cold urticaria | 1 | 4.32 ( 0.6 - 30.89 ) | 4.32 ( 2.54 ) | 4.3 ( 0.83 ) | 2.1 ( 0.05 ) |
| Ovarian calcification | 1 | 108.8 ( 13.1 - 903.75 ) | 108.8 ( 91.55 ) | 93.4 ( 15.89 ) | 6.55 ( 4.26 ) |
| Gastrointestinal wall thinning | 1 | 17.18 ( 2.36 - 125.12 ) | 17.18 ( 14.85 ) | 16.76 ( 3.18 ) | 4.07 ( 1.98 ) |
| Perineal ulceration | 1 | 11.25 ( 1.56 - 81.26 ) | 11.25 ( 9.19 ) | 11.08 ( 2.12 ) | 3.47 ( 1.4 ) |
| Neurosyphilis | 1 | 6.53 ( 0.91 - 46.8 ) | 6.53 ( 4.63 ) | 6.47 ( 1.25 ) | 2.69 ( 0.63 ) |
| Focal peritonitis | 1 | 19.2 ( 2.63 - 140.26 ) | 19.2 ( 16.76 ) | 18.68 ( 3.54 ) | 4.22 ( 2.13 ) |
| Mucocutaneous candidiasis | 1 | 12.8 ( 1.77 - 92.63 ) | 12.8 ( 10.67 ) | 12.57 ( 2.4 ) | 3.65 ( 1.57 ) |
| Hepatitis c antibody positive | 1 | 6.59 ( 0.92 - 47.28 ) | 6.59 ( 4.7 ) | 6.54 ( 1.26 ) | 2.71 ( 0.65 ) |
| Acute flaccid myelitis | 1 | 72.53 ( 9.19 - 572.53 ) | 72.53 ( 63.49 ) | 65.38 ( 11.61 ) | 6.03 ( 3.81 ) |
| Pain threshold decreased | 1 | 8.16 ( 1.14 - 58.64 ) | 8.16 ( 6.2 ) | 8.07 ( 1.55 ) | 3.01 ( 0.95 ) |
| Leukoplakia | 1 | 7.42 ( 1.03 - 53.25 ) | 7.42 ( 5.49 ) | 7.35 ( 1.41 ) | 2.88 ( 0.81 ) |
| Parathyroid gland operation | 1 | 8.7 ( 1.21 - 62.6 ) | 8.7 ( 6.73 ) | 8.6 ( 1.65 ) | 3.1 ( 1.04 ) |
| Vascular access site bruising | 1 | 16.32 ( 2.24 - 118.72 ) | 16.32 ( 14.03 ) | 15.95 ( 3.03 ) | 4 ( 1.91 ) |
| Red blood cells urine | 1 | 4.44 ( 0.62 - 31.74 ) | 4.44 ( 2.65 ) | 4.42 ( 0.85 ) | 2.14 ( 0.09 ) |
| Vasoactive intestinal polypeptide increased | 1 | 163.2 ( 18.24 - 1460.19 ) | 163.19 ( 128.96 ) | 130.75 ( 20.9 ) | 7.03 ( 4.67 ) |
| Mastoid effusion | 1 | 25.11 ( 3.41 - 185.03 ) | 25.11 ( 22.29 ) | 24.21 ( 4.55 ) | 4.6 ( 2.49 ) |
| Tongue thrust | 1 | 9.89 ( 1.37 - 71.26 ) | 9.89 ( 7.87 ) | 9.76 ( 1.87 ) | 3.29 ( 1.22 ) |
| Gallbladder adenocarcinoma | 1 | 15.18 ( 2.09 - 110.25 ) | 15.18 ( 12.95 ) | 14.86 ( 2.83 ) | 3.89 ( 1.81 ) |
| Administration site induration | 1 | 9.19 ( 1.28 - 66.18 ) | 9.19 ( 7.2 ) | 9.08 ( 1.74 ) | 3.18 ( 1.11 ) |
| Hepatic vascular thrombosis | 1 | 4.76 ( 0.67 - 34.07 ) | 4.76 ( 2.95 ) | 4.74 ( 0.91 ) | 2.24 ( 0.19 ) |
| Nasal vestibulitis | 1 | 16.74 ( 2.3 - 121.84 ) | 16.74 ( 14.43 ) | 16.34 ( 3.1 ) | 4.03 ( 1.94 ) |
| Skull fractured base | 1 | 4.84 ( 0.68 - 34.58 ) | 4.84 ( 3.02 ) | 4.81 ( 0.93 ) | 2.27 ( 0.21 ) |
| Non-hodgkin's lymphoma stage iv | 1 | 5.18 ( 0.72 - 37.07 ) | 5.18 ( 3.35 ) | 5.15 ( 0.99 ) | 2.36 ( 0.31 ) |
| Pigmentation lip | 1 | 12.8 ( 1.77 - 92.63 ) | 12.8 ( 10.67 ) | 12.57 ( 2.4 ) | 3.65 ( 1.57 ) |
| Vaccination site induration | 1 | 36.27 ( 4.84 - 271.67 ) | 36.27 ( 32.49 ) | 34.41 ( 6.38 ) | 5.1 ( 2.96 ) |
| Increased insulin requirement | 1 | 4.98 ( 0.7 - 35.64 ) | 4.98 ( 3.16 ) | 4.95 ( 0.95 ) | 2.31 ( 0.25 ) |
| Limb traumatic amputation | 1 | 4.6 ( 0.64 - 32.86 ) | 4.6 ( 2.79 ) | 4.57 ( 0.88 ) | 2.19 ( 0.14 ) |
| Umbilical cord short | 1 | 65.28 ( 8.36 - 509.97 ) | 65.28 ( 57.54 ) | 59.43 ( 10.64 ) | 5.89 ( 3.69 ) |
| Muscle contusion | 1 | 9.6 ( 1.33 - 69.14 ) | 9.6 ( 7.59 ) | 9.47 ( 1.82 ) | 3.24 ( 1.17 ) |
| Periorbital abscess | 1 | 21.06 ( 2.87 - 154.26 ) | 21.06 ( 18.51 ) | 20.43 ( 3.86 ) | 4.35 ( 2.25 ) |
| Branchial cyst | 1 | 36.27 ( 4.84 - 271.67 ) | 36.27 ( 32.49 ) | 34.41 ( 6.38 ) | 5.1 ( 2.96 ) |
| Double stranded dna antibody | 1 | 21.06 ( 2.87 - 154.26 ) | 21.06 ( 18.51 ) | 20.43 ( 3.86 ) | 4.35 ( 2.25 ) |
| Penile ulceration | 1 | 4.21 ( 0.59 - 30.09 ) | 4.21 ( 2.43 ) | 4.19 ( 0.81 ) | 2.07 ( 0.01 ) |
| Autoimmune uveitis | 1 | 11.87 ( 1.64 - 85.77 ) | 11.87 ( 9.78 ) | 11.67 ( 2.23 ) | 3.55 ( 1.47 ) |
| Unwanted pregnancy | 1 | 10.36 ( 1.44 - 74.71 ) | 10.36 ( 8.33 ) | 10.22 ( 1.96 ) | 3.35 ( 1.28 ) |
| Gamma-glutamyltransferase | 1 | 15.54 ( 2.14 - 112.93 ) | 15.54 ( 13.29 ) | 15.2 ( 2.89 ) | 3.93 ( 1.84 ) |
| Blood triglycerides | 1 | 43.52 ( 5.75 - 329.47 ) | 43.52 ( 38.94 ) | 40.86 ( 7.51 ) | 5.35 ( 3.2 ) |
| Genital disorder | 1 | 7.42 ( 1.03 - 53.25 ) | 7.42 ( 5.49 ) | 7.35 ( 1.41 ) | 2.88 ( 0.81 ) |
| Vulval ulceration | 1 | 4.27 ( 0.6 - 30.48 ) | 4.27 ( 2.48 ) | 4.25 ( 0.82 ) | 2.09 ( 0.03 ) |
| Oral bacterial infection | 1 | 6.28 ( 0.88 - 44.98 ) | 6.28 ( 4.39 ) | 6.23 ( 1.2 ) | 2.64 ( 0.58 ) |
| Sinusitis aspergillus | 1 | 7.5 ( 1.05 - 53.87 ) | 7.5 ( 5.57 ) | 7.43 ( 1.43 ) | 2.89 ( 0.83 ) |
| Urticaria cholinergic | 1 | 11.66 ( 1.61 - 84.21 ) | 11.66 ( 9.57 ) | 11.47 ( 2.19 ) | 3.52 ( 1.44 ) |
| Spinal meningeal cyst | 1 | 16.74 ( 2.3 - 121.84 ) | 16.74 ( 14.43 ) | 16.34 ( 3.1 ) | 4.03 ( 1.94 ) |
| Papillary renal cell carcinoma | 1 | 6.1 ( 0.85 - 43.71 ) | 6.1 ( 4.23 ) | 6.05 ( 1.17 ) | 2.6 ( 0.54 ) |
| Lobular breast carcinoma in situ | 1 | 10.2 ( 1.41 - 73.53 ) | 10.2 ( 8.17 ) | 10.06 ( 1.93 ) | 3.33 ( 1.26 ) |
| Oral viral infection | 1 | 8.82 ( 1.23 - 63.46 ) | 8.82 ( 6.84 ) | 8.72 ( 1.67 ) | 3.12 ( 1.06 ) |
| Bronchopulmonary disease | 1 | 15.18 ( 2.09 - 110.25 ) | 15.18 ( 12.95 ) | 14.86 ( 2.83 ) | 3.89 ( 1.81 ) |
| Immunology test | 1 | 93.26 ( 11.47 - 758 ) | 93.25 ( 79.86 ) | 81.72 ( 14.16 ) | 6.35 ( 4.1 ) |
| Tongue fungal infection | 1 | 4.44 ( 0.62 - 31.74 ) | 4.44 ( 2.65 ) | 4.42 ( 0.85 ) | 2.14 ( 0.09 ) |
| Follicular mucinosis | 1 | 65.28 ( 8.36 - 509.97 ) | 65.28 ( 57.54 ) | 59.43 ( 10.64 ) | 5.89 ( 3.69 ) |
| Eye infarction | 1 | 10.36 ( 1.44 - 74.71 ) | 10.36 ( 8.33 ) | 10.22 ( 1.96 ) | 3.35 ( 1.28 ) |
| Ectrodactyly | 1 | 13.32 ( 1.84 - 96.48 ) | 13.32 ( 11.17 ) | 13.08 ( 2.49 ) | 3.71 ( 1.63 ) |
| Pinguecula | 1 | 13.06 ( 1.8 - 94.51 ) | 13.06 ( 10.91 ) | 12.82 ( 2.45 ) | 3.68 ( 1.6 ) |
| Viiith nerve injury | 1 | 13.89 ( 1.92 - 100.67 ) | 13.89 ( 11.71 ) | 13.62 ( 2.6 ) | 3.77 ( 1.69 ) |
| Taeniasis | 1 | 27.2 ( 3.68 - 201.07 ) | 27.2 ( 24.23 ) | 26.15 ( 4.9 ) | 4.71 ( 2.59 ) |
| Oral mucosal roughening | 1 | 10.88 ( 1.51 - 78.51 ) | 10.88 ( 8.82 ) | 10.72 ( 2.05 ) | 3.42 ( 1.35 ) |
| Seminoma | 1 | 4.35 ( 0.61 - 31.1 ) | 4.35 ( 2.56 ) | 4.33 ( 0.84 ) | 2.11 ( 0.06 ) |
| Pityriasis rubra pilaris | 1 | 4.53 ( 0.63 - 32.4 ) | 4.53 ( 2.73 ) | 4.51 ( 0.87 ) | 2.17 ( 0.12 ) |
| Abdominal fat apron | 1 | 7.86 ( 1.09 - 56.5 ) | 7.86 ( 5.92 ) | 7.78 ( 1.49 ) | 2.96 ( 0.9 ) |
| Neonatal pneumothorax | 1 | 14.84 ( 2.04 - 107.69 ) | 14.84 ( 12.62 ) | 14.53 ( 2.77 ) | 3.86 ( 1.78 ) |
| Ovarian rupture | 1 | 10.04 ( 1.39 - 72.38 ) | 10.04 ( 8.02 ) | 9.91 ( 1.9 ) | 3.31 ( 1.24 ) |
| Genital injury | 1 | 7.5 ( 1.05 - 53.87 ) | 7.5 ( 5.57 ) | 7.43 ( 1.43 ) | 2.89 ( 0.83 ) |
| Cervix neoplasm | 1 | 8.26 ( 1.15 - 59.39 ) | 8.26 ( 6.3 ) | 8.17 ( 1.57 ) | 3.03 ( 0.97 ) |
| Benign familial pemphigus | 1 | 20.4 ( 2.79 - 149.29 ) | 20.4 ( 17.89 ) | 19.81 ( 3.75 ) | 4.31 ( 2.21 ) |
| Adnexa uteri cyst | 1 | 10.04 ( 1.39 - 72.38 ) | 10.04 ( 8.02 ) | 9.91 ( 1.9 ) | 3.31 ( 1.24 ) |
| Lateral medullary syndrome | 1 | 10.36 ( 1.44 - 74.71 ) | 10.36 ( 8.33 ) | 10.22 ( 1.96 ) | 3.35 ( 1.28 ) |
| Renal artery thrombosis | 1 | 4.32 ( 0.6 - 30.89 ) | 4.32 ( 2.54 ) | 4.3 ( 0.83 ) | 2.1 ( 0.05 ) |
| Tick-borne fever | 1 | 15.92 ( 2.19 - 115.75 ) | 15.92 ( 13.65 ) | 15.57 ( 2.96 ) | 3.96 ( 1.87 ) |
| Follicular thyroid cancer | 1 | 7.33 ( 1.02 - 52.65 ) | 7.33 ( 5.41 ) | 7.26 ( 1.4 ) | 2.86 ( 0.8 ) |
| Peripheral arterial reocclusion | 1 | 108.8 ( 13.1 - 903.75 ) | 108.8 ( 91.55 ) | 93.4 ( 15.89 ) | 6.55 ( 4.26 ) |
| Paradoxical psoriatic arthritis | 1 | 59.34 ( 7.66 - 459.68 ) | 59.34 ( 52.58 ) | 54.48 ( 9.82 ) | 5.77 ( 3.58 ) |
| Ear, nose and throat disorder | 1 | 5.26 ( 0.74 - 37.67 ) | 5.26 ( 3.43 ) | 5.23 ( 1.01 ) | 2.39 ( 0.33 ) |
| Cd4 lymphocytes abnormal | 1 | 5.06 ( 0.71 - 36.2 ) | 5.06 ( 3.23 ) | 5.03 ( 0.97 ) | 2.33 ( 0.27 ) |
| Antifungal treatment | 1 | 163.2 ( 18.24 - 1460.19 ) | 163.19 ( 128.96 ) | 130.75 ( 20.9 ) | 7.03 ( 4.67 ) |
| Stiff tongue | 1 | 11.25 ( 1.56 - 81.26 ) | 11.25 ( 9.19 ) | 11.08 ( 2.12 ) | 3.47 ( 1.4 ) |
| Oligoasthenozoospermia | 1 | 40.8 ( 5.41 - 307.66 ) | 40.8 ( 36.54 ) | 38.46 ( 7.09 ) | 5.27 ( 3.11 ) |
| Gingival injury | 1 | 5.35 ( 0.75 - 38.29 ) | 5.35 ( 3.51 ) | 5.32 ( 1.02 ) | 2.41 ( 0.35 ) |
| Carcinoid tumour pulmonary | 1 | 4.38 ( 0.61 - 31.31 ) | 4.38 ( 2.59 ) | 4.36 ( 0.84 ) | 2.12 ( 0.07 ) |
| Tongue abscess | 1 | 17.64 ( 2.42 - 128.59 ) | 17.64 ( 15.29 ) | 17.2 ( 3.26 ) | 4.1 ( 2.01 ) |
| Interleukin-2 receptor increased | 1 | 6.1 ( 0.85 - 43.71 ) | 6.1 ( 4.23 ) | 6.05 ( 1.17 ) | 2.6 ( 0.54 ) |
| Urea cycle disorder | 1 | 32.64 ( 4.38 - 243.21 ) | 32.64 ( 29.21 ) | 31.13 ( 5.8 ) | 4.96 ( 2.83 ) |
| Biopsy site unspecified abnormal | 1 | 14.84 ( 2.04 - 107.69 ) | 14.84 ( 12.62 ) | 14.53 ( 2.77 ) | 3.86 ( 1.78 ) |
| Endoscopy abnormal | 1 | 10.53 ( 1.46 - 75.93 ) | 10.53 ( 8.49 ) | 10.38 ( 1.99 ) | 3.38 ( 1.3 ) |
| Bladder catheter removal | 1 | 36.27 ( 4.84 - 271.67 ) | 36.27 ( 32.49 ) | 34.41 ( 6.38 ) | 5.1 ( 2.96 ) |
| Hairy cell leukaemia | 1 | 5.73 ( 0.8 - 41 ) | 5.73 ( 3.87 ) | 5.68 ( 1.09 ) | 2.51 ( 0.45 ) |
| Cervix cancer metastatic | 1 | 10.2 ( 1.41 - 73.53 ) | 10.2 ( 8.17 ) | 10.06 ( 1.93 ) | 3.33 ( 1.26 ) |
| Bladder ulcer | 1 | 23.31 ( 3.17 - 171.36 ) | 23.31 ( 20.62 ) | 22.54 ( 4.25 ) | 4.49 ( 2.39 ) |
| Fascial rupture | 1 | 21.06 ( 2.87 - 154.26 ) | 21.06 ( 18.51 ) | 20.43 ( 3.86 ) | 4.35 ( 2.25 ) |
| Follicle centre lymphoma, follicular grade i, ii, iii stage iv | 1 | 163.2 ( 18.24 - 1460.19 ) | 163.19 ( 128.96 ) | 130.75 ( 20.9 ) | 7.03 ( 4.67 ) |
| Paradoxical skin reaction | 1 | 46.63 ( 6.13 - 354.6 ) | 46.63 ( 41.67 ) | 43.58 ( 7.98 ) | 5.45 ( 3.28 ) |
| Gastritis viral | 1 | 9.46 ( 1.31 - 68.12 ) | 9.46 ( 7.46 ) | 9.34 ( 1.79 ) | 3.22 ( 1.15 ) |
| Chest wall abscess | 1 | 5.58 ( 0.78 - 39.94 ) | 5.58 ( 3.73 ) | 5.54 ( 1.07 ) | 2.47 ( 0.41 ) |
| Genital infection female | 1 | 7.02 ( 0.98 - 50.36 ) | 7.02 ( 5.11 ) | 6.96 ( 1.34 ) | 2.8 ( 0.74 ) |
| Lens disorder | 1 | 4.87 ( 0.68 - 34.84 ) | 4.87 ( 3.05 ) | 4.84 ( 0.93 ) | 2.28 ( 0.22 ) |
| Raoultella test positive | 1 | 72.53 ( 9.19 - 572.53 ) | 72.53 ( 63.49 ) | 65.38 ( 11.61 ) | 6.03 ( 3.81 ) |
| Splenic artery aneurysm | 1 | 5.93 ( 0.83 - 42.51 ) | 5.93 ( 4.07 ) | 5.89 ( 1.13 ) | 2.56 ( 0.5 ) |
| Uterine leiomyoma rupture | 1 | Inf ( NaN - Inf ) | Inf ( 652.77 ) | 653.77 ( 0 ) | 9.35 ( 6.47 ) |
| Rectal neoplasm | 1 | 4.47 ( 0.63 - 31.96 ) | 4.47 ( 2.68 ) | 4.45 ( 0.86 ) | 2.15 ( 0.1 ) |
| Hla-b*27 positive | 1 | 6.1 ( 0.85 - 43.71 ) | 6.1 ( 4.23 ) | 6.05 ( 1.17 ) | 2.6 ( 0.54 ) |
| Autoimmune blistering disease | 1 | 26.11 ( 3.54 - 192.71 ) | 26.11 ( 23.22 ) | 25.15 ( 4.72 ) | 4.65 ( 2.54 ) |
| Breast injury | 1 | 6.1 ( 0.85 - 43.71 ) | 6.1 ( 4.23 ) | 6.05 ( 1.17 ) | 2.6 ( 0.54 ) |
| Nasal sinus cancer | 1 | 5.1 ( 0.71 - 36.48 ) | 5.1 ( 3.27 ) | 5.07 ( 0.98 ) | 2.34 ( 0.29 ) |
| Parapharyngeal space infection | 1 | 36.27 ( 4.84 - 271.67 ) | 36.27 ( 32.49 ) | 34.41 ( 6.38 ) | 5.1 ( 2.96 ) |
| Alcoholic liver disease | 1 | 4.44 ( 0.62 - 31.74 ) | 4.44 ( 2.65 ) | 4.42 ( 0.85 ) | 2.14 ( 0.09 ) |
| Prostatic dysplasia | 1 | 20.4 ( 2.79 - 149.29 ) | 20.4 ( 17.89 ) | 19.81 ( 3.75 ) | 4.31 ( 2.21 ) |
| Respiratory syncytial virus bronchitis | 1 | 9.74 ( 1.35 - 70.19 ) | 9.74 ( 7.73 ) | 9.61 ( 1.84 ) | 3.27 ( 1.2 ) |
| Eczema infected | 1 | 5.06 ( 0.71 - 36.2 ) | 5.06 ( 3.23 ) | 5.03 ( 0.97 ) | 2.33 ( 0.27 ) |
| Myelodysplastic syndrome with multilineage dysplasia | 1 | 36.27 ( 4.84 - 271.67 ) | 36.27 ( 32.49 ) | 34.41 ( 6.38 ) | 5.1 ( 2.96 ) |
| Splenic injury | 1 | 4.7 ( 0.66 - 33.58 ) | 4.7 ( 2.89 ) | 4.67 ( 0.9 ) | 2.22 ( 0.17 ) |
| Bone formation decreased | 1 | 8.82 ( 1.23 - 63.46 ) | 8.82 ( 6.84 ) | 8.72 ( 1.67 ) | 3.12 ( 1.06 ) |
| Oesophageal squamous cell carcinoma metastatic | 1 | 72.53 ( 9.19 - 572.53 ) | 72.53 ( 63.49 ) | 65.38 ( 11.61 ) | 6.03 ( 3.81 ) |
| External ear cellulitis | 1 | 8.82 ( 1.23 - 63.46 ) | 8.82 ( 6.84 ) | 8.72 ( 1.67 ) | 3.12 ( 1.06 ) |
| Alcoholic pancreatitis | 1 | 7.17 ( 1 - 51.48 ) | 7.17 ( 5.26 ) | 7.11 ( 1.37 ) | 2.83 ( 0.77 ) |
| Intestinal tuberculosis | 1 | 4.91 ( 0.69 - 35.1 ) | 4.91 ( 3.09 ) | 4.88 ( 0.94 ) | 2.29 ( 0.23 ) |
| Pulmonary venous thrombosis | 1 | 7.42 ( 1.03 - 53.25 ) | 7.42 ( 5.49 ) | 7.35 ( 1.41 ) | 2.88 ( 0.81 ) |
| Tattoo associated skin reaction | 1 | 81.6 ( 10.21 - 652.44 ) | 81.6 ( 70.76 ) | 72.64 ( 12.76 ) | 6.18 ( 3.95 ) |
| Prostatic abscess | 1 | 4.73 ( 0.66 - 33.82 ) | 4.73 ( 2.92 ) | 4.7 ( 0.91 ) | 2.23 ( 0.18 ) |
| Myelodysplastic syndrome with excess blasts | 1 | 11.25 ( 1.56 - 81.26 ) | 11.25 ( 9.19 ) | 11.08 ( 2.12 ) | 3.47 ( 1.4 ) |
| Suspected drug-induced liver injury | 1 | 7.5 ( 1.05 - 53.87 ) | 7.5 ( 5.57 ) | 7.43 ( 1.43 ) | 2.89 ( 0.83 ) |
| Vocal cordectomy | 1 | 163.2 ( 18.24 - 1460.19 ) | 163.19 ( 128.96 ) | 130.75 ( 20.9 ) | 7.03 ( 4.67 ) |
| Arterial therapeutic procedure | 1 | 5.99 ( 0.84 - 42.9 ) | 5.99 ( 4.12 ) | 5.94 ( 1.14 ) | 2.57 ( 0.51 ) |
| Cortisol abnormal | 1 | 5.06 ( 0.71 - 36.2 ) | 5.06 ( 3.23 ) | 5.03 ( 0.97 ) | 2.33 ( 0.27 ) |
| Body mass index abnormal | 1 | 4.91 ( 0.69 - 35.1 ) | 4.91 ( 3.09 ) | 4.88 ( 0.94 ) | 2.29 ( 0.23 ) |
| Conjunctival operation | 1 | 326.39 ( 29.59 - 3599.74 ) | 326.39 ( 216.26 ) | 217.92 ( 29.24 ) | 7.77 ( 5.27 ) |
| Haematological neoplasm | 1 | 21.76 ( 2.97 - 159.57 ) | 21.76 ( 19.17 ) | 21.09 ( 3.98 ) | 4.4 ( 2.3 ) |
| Oesophageal mass | 1 | 6.28 ( 0.88 - 44.98 ) | 6.28 ( 4.39 ) | 6.23 ( 1.2 ) | 2.64 ( 0.58 ) |
| Ear infection staphylococcal | 1 | 8.59 ( 1.19 - 61.77 ) | 8.59 ( 6.62 ) | 8.49 ( 1.63 ) | 3.09 ( 1.02 ) |
| Herpangina | 1 | 15.18 ( 2.09 - 110.25 ) | 15.18 ( 12.95 ) | 14.86 ( 2.83 ) | 3.89 ( 1.81 ) |
| Klebsiella urinary tract infection | 1 | 6.16 ( 0.86 - 44.13 ) | 6.16 ( 4.28 ) | 6.11 ( 1.18 ) | 2.61 ( 0.55 ) |
| Nail deformation | 1 | 26.11 ( 3.54 - 192.71 ) | 26.11 ( 23.22 ) | 25.15 ( 4.72 ) | 4.65 ( 2.54 ) |
| Peripheral spondyloarthritis | 1 | 14.19 ( 1.96 - 102.91 ) | 14.19 ( 12 ) | 13.91 ( 2.65 ) | 3.8 ( 1.72 ) |
| Anti-thyroid antibody increased | 1 | 10.2 ( 1.41 - 73.53 ) | 10.2 ( 8.17 ) | 10.06 ( 1.93 ) | 3.33 ( 1.26 ) |
| Endocrine test abnormal | 1 | 54.4 ( 7.07 - 418.38 ) | 54.4 ( 48.38 ) | 50.29 ( 9.12 ) | 5.65 ( 3.47 ) |
| Sleeve gastrectomy | 1 | 5.49 ( 0.77 - 39.27 ) | 5.49 ( 3.64 ) | 5.45 ( 1.05 ) | 2.45 ( 0.39 ) |
| Tongue neoplasm | 1 | 4.29 ( 0.6 - 30.69 ) | 4.29 ( 2.51 ) | 4.27 ( 0.82 ) | 2.1 ( 0.04 ) |
| Fascial infection | 1 | 21.76 ( 2.97 - 159.57 ) | 21.76 ( 19.17 ) | 21.09 ( 3.98 ) | 4.4 ( 2.3 ) |
| Sars-cov-2 test | 1 | 7.02 ( 0.98 - 50.36 ) | 7.02 ( 5.11 ) | 6.96 ( 1.34 ) | 2.8 ( 0.74 ) |
| Jaw cyst | 1 | 6.66 ( 0.93 - 47.76 ) | 6.66 ( 4.76 ) | 6.6 ( 1.27 ) | 2.72 ( 0.66 ) |
| Overgrowth fungal | 1 | 8.82 ( 1.23 - 63.46 ) | 8.82 ( 6.84 ) | 8.72 ( 1.67 ) | 3.12 ( 1.06 ) |
| Liver operation | 1 | 4.29 ( 0.6 - 30.69 ) | 4.29 ( 2.51 ) | 4.27 ( 0.82 ) | 2.1 ( 0.04 ) |
| Fallopian tube cyst | 1 | 10.7 ( 1.48 - 77.2 ) | 10.7 ( 8.65 ) | 10.54 ( 2.02 ) | 3.4 ( 1.33 ) |

Abbreviation: ROR, reporting odds ratio; PRR, proportional reporting ratio; EBGM, empirical Bayesian geometric mean; EBGM05, the lower limit of the 95% CI of EBGM; IC, information component; IC025, the lower limit of the 95% CI of the IC; CI, confidence interval; PT,preferred term.
