# Supplementary material for: Real-world safety assessment of Ixekizumab based on the FDA Adverse Event Reporting System (FAERS)
Source: PLoS One. 2025 May 23;20(5):e0323973. doi: 10.1371/journal.pone.0323973 (PMC12101745; doi:10.1371/journal.pone.0323973)
Supplement: S4 Table — (DOCX) [file pone.0323973.s004.docx]

Supplementary Table 4:

Top 50 most frequent positive signal adverse events of Ixekizumab at the preferred term (PT) level in males from FAERS data

| PT | Case numbers | ROR(95%CI) | PRR(χ^2^) | EBGM(EBGM05) | IC(IC025) |
| --- | --- | --- | --- | --- | --- |
| Injection site pain | 1,239 | 16.83 ( 15.88 - 17.84 ) | 15.92 ( 16896.24 ) | 15.5 ( 14.76 ) | 3.95 ( 3.87 ) |
| Psoriasis | 970 | 18.31 ( 17.15 - 19.55 ) | 17.53 ( 14694.37 ) | 17.02 ( 16.12 ) | 4.09 ( 3.99 ) |
| Drug ineffective | 841 | 1.86 ( 1.74 - 1.99 ) | 1.83 ( 321.15 ) | 1.83 ( 1.72 ) | 0.87 ( 0.77 ) |
| Injection site erythema | 502 | 29.12 ( 26.59 - 31.88 ) | 28.46 ( 12659.29 ) | 27.11 ( 25.13 ) | 4.76 ( 4.63 ) |
| Injection site swelling | 434 | 28.54 ( 25.89 - 31.46 ) | 27.99 ( 10756.22 ) | 26.68 ( 24.59 ) | 4.74 ( 4.59 ) |
| Injection site reaction | 379 | 29.91 ( 26.95 - 33.2 ) | 29.4 ( 9878.77 ) | 27.97 ( 25.63 ) | 4.81 ( 4.65 ) |
| Incorrect dose administered | 331 | 3.93 ( 3.52 - 4.38 ) | 3.88 ( 705.51 ) | 3.86 ( 3.52 ) | 1.95 ( 1.79 ) |
| Product dose omission issue | 295 | 2.57 ( 2.29 - 2.88 ) | 2.55 ( 277.47 ) | 2.54 ( 2.31 ) | 1.34 ( 1.18 ) |
| Injection site haemorrhage | 253 | 9.56 ( 8.44 - 10.84 ) | 9.46 ( 1884.74 ) | 9.32 ( 8.39 ) | 3.22 ( 3.04 ) |
| Covid-19 | 250 | 2.66 ( 2.35 - 3.02 ) | 2.64 ( 255.27 ) | 2.64 ( 2.37 ) | 1.4 ( 1.21 ) |
| Injection site mass | 230 | 16.38 ( 14.36 - 18.69 ) | 16.22 ( 3192.08 ) | 15.78 ( 14.13 ) | 3.98 ( 3.79 ) |
| Arthralgia | 221 | 1.85 ( 1.62 - 2.11 ) | 1.84 ( 84.59 ) | 1.83 ( 1.64 ) | 0.88 ( 0.68 ) |
| Therapy interrupted | 215 | 7.62 ( 6.66 - 8.73 ) | 7.56 ( 1208.52 ) | 7.47 ( 6.67 ) | 2.9 ( 2.7 ) |
| Rash | 202 | 1.42 ( 1.24 - 1.64 ) | 1.42 ( 25.26 ) | 1.42 ( 1.26 ) | 0.51 ( 0.3 ) |
| Inappropriate schedule of product administration | 200 | 2.57 ( 2.23 - 2.95 ) | 2.55 ( 188.37 ) | 2.54 ( 2.26 ) | 1.35 ( 1.14 ) |
| Pruritus | 196 | 1.77 ( 1.54 - 2.04 ) | 1.76 ( 64.57 ) | 1.76 ( 1.56 ) | 0.81 ( 0.61 ) |
| Nasopharyngitis | 171 | 3.14 ( 2.7 - 3.65 ) | 3.12 ( 246.09 ) | 3.11 ( 2.74 ) | 1.64 ( 1.42 ) |
| Injection site pruritus | 161 | 17.78 ( 15.19 - 20.81 ) | 17.65 ( 2451.94 ) | 17.14 ( 15.02 ) | 4.1 ( 3.87 ) |
| Psoriatic arthropathy | 155 | 11.31 ( 9.64 - 13.26 ) | 11.23 ( 1417.08 ) | 11.03 ( 9.65 ) | 3.46 ( 3.23 ) |
| Infection | 155 | 3.22 ( 2.75 - 3.77 ) | 3.2 ( 234.01 ) | 3.19 ( 2.79 ) | 1.67 ( 1.44 ) |
| Therapy cessation | 148 | 5.84 ( 4.96 - 6.87 ) | 5.81 ( 583.52 ) | 5.76 ( 5.03 ) | 2.53 ( 2.29 ) |
| Cellulitis | 132 | 6.97 ( 5.87 - 8.29 ) | 6.94 ( 663.12 ) | 6.86 ( 5.94 ) | 2.78 ( 2.53 ) |
| Injection site urticaria | 129 | 34.67 ( 29.01 - 41.44 ) | 34.47 ( 3946.58 ) | 32.5 ( 28 ) | 5.02 ( 4.76 ) |
| Injection site rash | 128 | 24.85 ( 20.81 - 29.68 ) | 24.71 ( 2787.87 ) | 23.69 ( 20.42 ) | 4.57 ( 4.31 ) |
| Urticaria | 119 | 2.93 ( 2.45 - 3.51 ) | 2.92 ( 149.66 ) | 2.91 ( 2.5 ) | 1.54 ( 1.28 ) |
| Influenza | 114 | 3.16 ( 2.63 - 3.8 ) | 3.15 ( 166.64 ) | 3.14 ( 2.69 ) | 1.65 ( 1.38 ) |
| Hypersensitivity | 107 | 2.41 ( 1.99 - 2.91 ) | 2.4 ( 87.02 ) | 2.39 ( 2.04 ) | 1.26 ( 0.98 ) |
| Sinusitis | 105 | 4.73 ( 3.9 - 5.74 ) | 4.72 ( 305.14 ) | 4.68 ( 3.99 ) | 2.23 ( 1.95 ) |
| Injection site bruising | 98 | 6.42 ( 5.26 - 7.84 ) | 6.4 ( 441.38 ) | 6.33 ( 5.36 ) | 2.66 ( 2.37 ) |
| Underdose | 95 | 3.3 ( 2.7 - 4.04 ) | 3.29 ( 150.76 ) | 3.28 ( 2.77 ) | 1.71 ( 1.42 ) |
| Injection site warmth | 94 | 44.73 ( 36.24 - 55.22 ) | 44.54 ( 3702.27 ) | 41.29 ( 34.62 ) | 5.37 ( 5.06 ) |
| Arthritis | 91 | 4.26 ( 3.47 - 5.24 ) | 4.25 ( 224.6 ) | 4.22 ( 3.55 ) | 2.08 ( 1.78 ) |
| Cardiac failure congestive | 86 | 4.57 ( 3.69 - 5.65 ) | 4.55 ( 236.62 ) | 4.52 ( 3.79 ) | 2.18 ( 1.87 ) |
| Erythema | 85 | 1.34 ( 1.08 - 1.65 ) | 1.34 ( 7.16 ) | 1.33 ( 1.12 ) | 0.42 ( 0.1 ) |
| Illness | 84 | 2.76 ( 2.22 - 3.42 ) | 2.75 ( 93.16 ) | 2.74 ( 2.29 ) | 1.45 ( 1.14 ) |
| Accidental underdose | 83 | 14.58 ( 11.72 - 18.14 ) | 14.53 ( 1019.33 ) | 14.19 ( 11.82 ) | 3.83 ( 3.51 ) |
| Myocardial infarction | 82 | 1.49 ( 1.2 - 1.85 ) | 1.49 ( 13.16 ) | 1.49 ( 1.24 ) | 0.57 ( 0.26 ) |
| Therapeutic product effect incomplete | 75 | 2.28 ( 1.82 - 2.86 ) | 2.27 ( 53.37 ) | 2.27 ( 1.88 ) | 1.18 ( 0.85 ) |
| Joint swelling | 68 | 2.15 ( 1.69 - 2.73 ) | 2.15 ( 41.58 ) | 2.14 ( 1.76 ) | 1.1 ( 0.75 ) |
| Ear infection | 68 | 9.36 ( 7.36 - 11.9 ) | 9.33 ( 497.6 ) | 9.19 ( 7.52 ) | 3.2 ( 2.85 ) |
| Therapeutic product effect decreased | 67 | 3.94 ( 3.1 - 5.02 ) | 3.93 ( 145.74 ) | 3.91 ( 3.2 ) | 1.97 ( 1.62 ) |
| Therapy non-responder | 65 | 3.21 ( 2.52 - 4.1 ) | 3.21 ( 98.2 ) | 3.19 ( 2.6 ) | 1.68 ( 1.32 ) |
| Oral candidiasis | 61 | 17 ( 13.17 - 21.94 ) | 16.96 ( 888.83 ) | 16.48 ( 13.31 ) | 4.04 ( 3.67 ) |
| Skin exfoliation | 61 | 2.11 ( 1.64 - 2.71 ) | 2.11 ( 35.41 ) | 2.1 ( 1.7 ) | 1.07 ( 0.7 ) |
| Ejection fraction decreased | 56 | 7.76 ( 5.96 - 10.11 ) | 7.75 ( 324.59 ) | 7.65 ( 6.14 ) | 2.94 ( 2.55 ) |
| Herpes zoster | 55 | 3.42 ( 2.62 - 4.46 ) | 3.41 ( 93.3 ) | 3.4 ( 2.72 ) | 1.76 ( 1.38 ) |
| Upper respiratory tract infection | 54 | 4.25 ( 3.25 - 5.55 ) | 4.24 ( 132.67 ) | 4.21 ( 3.37 ) | 2.08 ( 1.68 ) |
| Swelling | 54 | 2.16 ( 1.65 - 2.83 ) | 2.16 ( 33.54 ) | 2.16 ( 1.72 ) | 1.11 ( 0.72 ) |
| Injection site discomfort | 54 | 15.25 ( 11.63 - 19.99 ) | 15.21 ( 697.81 ) | 14.83 ( 11.82 ) | 3.89 ( 3.5 ) |
| Inflammatory bowel disease | 51 | 26.13 ( 19.72 - 34.61 ) | 26.07 ( 1174.08 ) | 24.94 ( 19.71 ) | 4.64 ( 4.23 ) |

Abbreviation: ROR, reporting odds ratio; PRR, proportional reporting ratio; EBGM, empirical Bayesian geometric mean; EBGM05, the lower limit of the 95% CI of EBGM; IC, information component; IC025, the lower limit of the 95% CI of the IC; CI, confidence interval; PT,preferred term; AEs, adverse events.
