# Supplementary material for: Real-world safety assessment of Ixekizumab based on the FDA Adverse Event Reporting System (FAERS)
Source: PLoS One. 2025 May 23;20(5):e0323973. doi: 10.1371/journal.pone.0323973 (PMC12101745; doi:10.1371/journal.pone.0323973)
Supplement: S5 Table — (DOCX) [file pone.0323973.s005.docx]

Supplementary Table 5:

Top 50 most frequent positive signal adverse events of Ixekizumab at the PT level in females from FAERS data

| PT | Case numbers | ROR(95%CI) | PRR(χ^2^) | EBGM(EBGM05) | IC(IC025) |
| --- | --- | --- | --- | --- | --- |
| Injection site pain | 2,109 | 11.86 ( 11.35 - 12.4 ) | 11.23 ( 19353.44 ) | 11.02 ( 10.62 ) | 3.46 ( 3.4 ) |
| Injection site erythema | 1,369 | 19.63 ( 18.58 - 20.74 ) | 18.93 ( 22508.65 ) | 18.32 ( 17.5 ) | 4.2 ( 4.11 ) |
| Drug ineffective | 1,249 | 1.5 ( 1.42 - 1.59 ) | 1.48 ( 201.14 ) | 1.48 ( 1.41 ) | 0.57 ( 0.48 ) |
| Psoriasis | 1,230 | 14.32 ( 13.52 - 15.17 ) | 13.87 ( 14353.97 ) | 13.55 ( 12.91 ) | 3.76 ( 3.68 ) |
| Injection site swelling | 999 | 22.17 ( 20.79 - 23.64 ) | 21.59 ( 18886.54 ) | 20.8 ( 19.71 ) | 4.38 ( 4.28 ) |
| Injection site reaction | 863 | 22.53 ( 21.04 - 24.14 ) | 22.02 ( 16659.5 ) | 21.2 ( 20.01 ) | 4.41 ( 4.3 ) |
| Covid-19 | 527 | 3.23 ( 2.96 - 3.52 ) | 3.19 ( 793.01 ) | 3.18 ( 2.96 ) | 1.67 ( 1.54 ) |
| Therapy interrupted | 495 | 9.31 ( 8.51 - 10.18 ) | 9.19 ( 3558.84 ) | 9.06 ( 8.4 ) | 3.18 ( 3.05 ) |
| Injection site pruritus | 494 | 11.17 ( 10.21 - 12.21 ) | 11.03 ( 4419.92 ) | 10.83 ( 10.04 ) | 3.44 ( 3.3 ) |
| Injection site urticaria | 424 | 24.51 ( 22.22 - 27.03 ) | 24.23 ( 9044.13 ) | 23.24 ( 21.41 ) | 4.54 ( 4.39 ) |
| Product dose omission issue | 413 | 2.04 ( 1.85 - 2.24 ) | 2.02 ( 214.3 ) | 2.02 ( 1.86 ) | 1.01 ( 0.87 ) |
| Incorrect dose administered | 411 | 3.05 ( 2.77 - 3.36 ) | 3.03 ( 557.01 ) | 3.02 ( 2.78 ) | 1.59 ( 1.45 ) |
| Arthralgia | 394 | 1.36 ( 1.23 - 1.5 ) | 1.35 ( 36.22 ) | 1.35 ( 1.24 ) | 0.43 ( 0.29 ) |
| Rash | 392 | 1.41 ( 1.28 - 1.56 ) | 1.41 ( 46.94 ) | 1.41 ( 1.3 ) | 0.49 ( 0.35 ) |
| Pruritus | 353 | 1.44 ( 1.3 - 1.6 ) | 1.44 ( 47.09 ) | 1.44 ( 1.32 ) | 0.52 ( 0.37 ) |
| Injection site warmth | 342 | 30.47 ( 27.31 - 33.99 ) | 30.19 ( 9145 ) | 28.65 ( 26.14 ) | 4.84 ( 4.68 ) |
| Nasopharyngitis | 306 | 2.29 ( 2.05 - 2.57 ) | 2.28 ( 220.24 ) | 2.28 ( 2.07 ) | 1.19 ( 1.02 ) |
| Injection site rash | 304 | 15.01 ( 13.39 - 16.83 ) | 14.89 ( 3835.8 ) | 14.52 ( 13.19 ) | 3.86 ( 3.69 ) |
| Injection site bruising | 302 | 5.39 ( 4.81 - 6.04 ) | 5.35 ( 1060.44 ) | 5.31 ( 4.83 ) | 2.41 ( 2.24 ) |
| Urticaria | 299 | 2.74 ( 2.44 - 3.07 ) | 2.72 ( 325.01 ) | 2.71 ( 2.47 ) | 1.44 ( 1.27 ) |
| Sinusitis | 299 | 3.72 ( 3.32 - 4.17 ) | 3.7 ( 585.41 ) | 3.68 ( 3.34 ) | 1.88 ( 1.71 ) |
| Injection site mass | 293 | 11.08 ( 9.87 - 12.45 ) | 11 ( 2613.03 ) | 10.8 ( 9.8 ) | 3.43 ( 3.26 ) |
| Urinary tract infection | 283 | 2.17 ( 1.93 - 2.43 ) | 2.16 ( 175.39 ) | 2.15 ( 1.95 ) | 1.11 ( 0.93 ) |
| Inappropriate schedule of product administration | 265 | 2 ( 1.77 - 2.25 ) | 1.99 ( 130.1 ) | 1.98 ( 1.79 ) | 0.99 ( 0.81 ) |
| Injection site haemorrhage | 259 | 5.25 ( 4.64 - 5.94 ) | 5.22 ( 876.63 ) | 5.18 ( 4.67 ) | 2.37 ( 2.19 ) |
| Psoriatic arthropathy | 246 | 7.85 ( 6.92 - 8.91 ) | 7.8 ( 1439.72 ) | 7.71 ( 6.93 ) | 2.95 ( 2.76 ) |
| Infection | 243 | 2.78 ( 2.45 - 3.15 ) | 2.77 ( 273.64 ) | 2.76 ( 2.48 ) | 1.46 ( 1.28 ) |
| Therapy cessation | 233 | 5.45 ( 4.79 - 6.2 ) | 5.42 ( 832.92 ) | 5.38 ( 4.83 ) | 2.43 ( 2.24 ) |
| Hypersensitivity | 225 | 1.69 ( 1.48 - 1.92 ) | 1.68 ( 62.25 ) | 1.68 ( 1.51 ) | 0.75 ( 0.56 ) |
| Illness | 219 | 2.62 ( 2.29 - 2.99 ) | 2.61 ( 217.27 ) | 2.6 ( 2.33 ) | 1.38 ( 1.19 ) |
| Pneumonia | 217 | 1.21 ( 1.06 - 1.38 ) | 1.21 ( 7.94 ) | 1.21 ( 1.08 ) | 0.27 ( 0.08 ) |
| Influenza | 180 | 2.26 ( 1.95 - 2.62 ) | 2.25 ( 125.42 ) | 2.25 ( 1.99 ) | 1.17 ( 0.95 ) |
| Ear infection | 147 | 7.38 ( 6.27 - 8.69 ) | 7.36 ( 797.34 ) | 7.27 ( 6.35 ) | 2.86 ( 2.62 ) |
| Cellulitis | 136 | 4.84 ( 4.09 - 5.73 ) | 4.82 ( 408.98 ) | 4.79 ( 4.16 ) | 2.26 ( 2.01 ) |
| Oropharyngeal pain | 133 | 1.99 ( 1.68 - 2.36 ) | 1.98 ( 64.79 ) | 1.98 ( 1.72 ) | 0.99 ( 0.74 ) |
| Fungal infection | 132 | 5.61 ( 4.72 - 6.66 ) | 5.59 ( 492.54 ) | 5.54 ( 4.8 ) | 2.47 ( 2.22 ) |
| Underdose | 131 | 2.46 ( 2.07 - 2.92 ) | 2.45 ( 112.55 ) | 2.45 ( 2.12 ) | 1.29 ( 1.04 ) |
| Bronchitis | 131 | 2.42 ( 2.04 - 2.87 ) | 2.41 ( 107.94 ) | 2.41 ( 2.08 ) | 1.27 ( 1.01 ) |
| Arthritis | 129 | 2.29 ( 1.93 - 2.73 ) | 2.29 ( 93.28 ) | 2.28 ( 1.97 ) | 1.19 ( 0.94 ) |
| Upper respiratory tract infection | 128 | 3.97 ( 3.34 - 4.73 ) | 3.96 ( 281.62 ) | 3.94 ( 3.41 ) | 1.98 ( 1.72 ) |
| Therapy non-responder | 111 | 3.54 ( 2.94 - 4.27 ) | 3.54 ( 200.86 ) | 3.52 ( 3.01 ) | 1.82 ( 1.54 ) |
| Injection site induration | 110 | 15.52 ( 12.83 - 18.76 ) | 15.47 ( 1448.03 ) | 15.07 ( 12.86 ) | 3.91 ( 3.64 ) |
| Accidental underdose | 107 | 9.4 ( 7.76 - 11.39 ) | 9.38 ( 787.46 ) | 9.24 ( 7.87 ) | 3.21 ( 2.93 ) |
| Therapeutic product effect decreased | 102 | 2.64 ( 2.17 - 3.21 ) | 2.64 ( 103.14 ) | 2.63 ( 2.23 ) | 1.39 ( 1.11 ) |
| Inflammation | 96 | 2.62 ( 2.14 - 3.2 ) | 2.61 ( 95.14 ) | 2.6 ( 2.2 ) | 1.38 ( 1.09 ) |
| Candida infection | 84 | 6.99 ( 5.64 - 8.67 ) | 6.98 ( 424.92 ) | 6.9 ( 5.76 ) | 2.79 ( 2.47 ) |
| Herpes zoster | 82 | 2.01 ( 1.62 - 2.5 ) | 2.01 ( 41.39 ) | 2 ( 1.67 ) | 1 ( 0.69 ) |
| Therapeutic product effect incomplete | 74 | 1.29 ( 1.02 - 1.62 ) | 1.29 ( 4.69 ) | 1.28 ( 1.06 ) | 0.36 ( 0.03 ) |
| Oral candidiasis | 72 | 9.49 ( 7.52 - 11.99 ) | 9.48 ( 536.67 ) | 9.33 ( 7.68 ) | 3.22 ( 2.88 ) |
| Stress | 72 | 1.35 ( 1.07 - 1.7 ) | 1.35 ( 6.47 ) | 1.35 ( 1.11 ) | 0.43 ( 0.09 ) |

Abbreviation: ROR, reporting odds ratio; PRR, proportional reporting ratio; EBGM, empirical Bayesian geometric mean; EBGM05, the lower limit of the 95% CI of EBGM; IC, information component; IC025, the lower limit of the 95% CI of the IC; CI, confidence interval; PT,preferred term; AEs, adverse events.
