# Supplementary material for: Real-world safety assessment of Ixekizumab based on the FDA Adverse Event Reporting System (FAERS)
Source: PLoS One. 2025 May 23;20(5):e0323973. doi: 10.1371/journal.pone.0323973 (PMC12101745; doi:10.1371/journal.pone.0323973)
Supplement: S6 Table — (DOCX) [file pone.0323973.s006.docx]

Supplementary Table 6:

Top 50 most frequent positive signal adverse events of Ixekizumab at the PT level in patients aged under 18 from FAERS data

| PT | Case numbers | ROR(95%CI) | PRR(χ^2^) | EBGM(EBGM05) | IC(IC025) |
| --- | --- | --- | --- | --- | --- |
| Injection site pain | 25 | 9.85 ( 6.58 - 14.75 ) | 9.37 ( 187.26 ) | 9.34 ( 6.66 ) | 3.22 ( 2.64 ) |
| Psoriasis | 22 | 82.03 ( 53.13 - 126.67 ) | 78.15 ( 1627.06 ) | 75.87 ( 52.75 ) | 6.25 ( 5.62 ) |
| Injection site reaction | 16 | 53.24 ( 32.18 - 88.1 ) | 51.42 ( 776.07 ) | 50.43 ( 33.09 ) | 5.66 ( 4.94 ) |
| Injection site erythema | 16 | 25.83 ( 15.65 - 42.63 ) | 24.96 ( 364.98 ) | 24.73 ( 16.26 ) | 4.63 ( 3.91 ) |
| Injection site swelling | 14 | 23.54 ( 13.8 - 40.17 ) | 22.86 ( 290.4 ) | 22.66 ( 14.49 ) | 4.5 ( 3.74 ) |
| Illness | 10 | 18.27 ( 9.74 - 34.27 ) | 17.9 ( 158.62 ) | 17.78 ( 10.51 ) | 4.15 ( 3.27 ) |
| Therapy interrupted | 10 | 37.98 ( 20.2 - 71.38 ) | 37.17 ( 347.15 ) | 36.65 ( 21.62 ) | 5.2 ( 4.31 ) |
| Fatigue | 9 | 3.82 ( 1.97 - 7.4 ) | 3.77 ( 18.36 ) | 3.76 ( 2.17 ) | 1.91 ( 0.99 ) |
| Injection site pruritus | 8 | 30.16 ( 14.93 - 60.92 ) | 29.65 ( 219.07 ) | 29.32 ( 16.28 ) | 4.87 ( 3.9 ) |
| Injection site rash | 7 | 40.32 ( 19 - 85.54 ) | 39.72 ( 260.28 ) | 39.13 ( 20.85 ) | 5.29 ( 4.25 ) |
| Diarrhoea | 7 | 2.19 ( 1.04 - 4.62 ) | 2.17 ( 4.46 ) | 2.17 ( 1.16 ) | 1.12 ( 0.09 ) |
| Nasopharyngitis | 6 | 5.44 ( 2.43 - 12.18 ) | 5.38 ( 21.39 ) | 5.37 ( 2.73 ) | 2.42 ( 1.32 ) |
| Pharyngitis streptococcal | 6 | 22.42 ( 9.99 - 50.35 ) | 22.14 ( 120.16 ) | 21.96 ( 11.16 ) | 4.46 ( 3.35 ) |
| Covid-19 | 6 | 6.42 ( 2.87 - 14.38 ) | 6.35 ( 27.02 ) | 6.34 ( 3.23 ) | 2.66 ( 1.56 ) |
| Product dose omission issue | 6 | 2.18 ( 0.98 - 4.89 ) | 2.17 ( 3.8 ) | 2.17 ( 1.1 ) | 1.12 ( 0.02 ) |
| Urticaria | 6 | 3 ( 1.34 - 6.71 ) | 2.97 ( 7.87 ) | 2.97 ( 1.51 ) | 1.57 ( 0.47 ) |
| Injection site urticaria | 5 | 19.97 ( 8.24 - 48.36 ) | 19.76 ( 88.42 ) | 19.62 ( 9.36 ) | 4.29 ( 3.1 ) |
| Drug hypersensitivity | 5 | 6.26 ( 2.59 - 15.14 ) | 6.21 ( 21.83 ) | 6.19 ( 2.96 ) | 2.63 ( 1.44 ) |
| Alopecia | 5 | 10.52 ( 4.35 - 25.44 ) | 10.42 ( 42.44 ) | 10.38 ( 4.96 ) | 3.38 ( 2.19 ) |
| Eye infection | 5 | 100.49 ( 40.94 - 246.7 ) | 99.41 ( 468.97 ) | 95.74 ( 45.16 ) | 6.58 ( 5.37 ) |
| Cough | 5 | 2.56 ( 1.06 - 6.19 ) | 2.55 ( 4.71 ) | 2.54 ( 1.22 ) | 1.35 ( 0.16 ) |
| Injection site mass | 5 | 24.59 ( 10.14 - 59.61 ) | 24.33 ( 110.87 ) | 24.11 ( 11.49 ) | 4.59 ( 3.4 ) |
| Oropharyngeal pain | 5 | 7.67 ( 3.17 - 18.55 ) | 7.6 ( 28.62 ) | 7.58 ( 3.62 ) | 2.92 ( 1.73 ) |
| Injection site warmth | 4 | 52.52 ( 19.44 - 141.95 ) | 52.08 ( 196.43 ) | 51.06 ( 22.22 ) | 5.67 ( 4.36 ) |
| Treatment failure | 4 | 5.73 ( 2.14 - 15.36 ) | 5.69 ( 15.46 ) | 5.68 ( 2.49 ) | 2.51 ( 1.21 ) |
| Skin infection | 4 | 35.07 ( 13.02 - 94.47 ) | 34.78 ( 129.5 ) | 34.32 ( 14.98 ) | 5.1 ( 3.79 ) |
| Accidental underdose | 3 | 79.02 ( 24.96 - 250.18 ) | 78.51 ( 222.78 ) | 76.21 ( 29.05 ) | 6.25 ( 4.78 ) |
| Injection site bruising | 3 | 7.02 ( 2.25 - 21.89 ) | 6.98 ( 15.35 ) | 6.97 ( 2.69 ) | 2.8 ( 1.35 ) |
| Sinusitis | 3 | 6.99 ( 2.24 - 21.79 ) | 6.95 ( 15.26 ) | 6.93 ( 2.68 ) | 2.79 ( 1.34 ) |
| Throat irritation | 3 | 16.2 ( 5.19 - 50.58 ) | 16.1 ( 42.23 ) | 16 ( 6.17 ) | 4 ( 2.55 ) |
| Vitiligo | 3 | 188.89 ( 58.28 - 612.19 ) | 187.66 ( 519.05 ) | 174.94 ( 65.4 ) | 7.45 ( 5.94 ) |
| Eye swelling | 3 | 6.62 ( 2.13 - 20.65 ) | 6.59 ( 14.2 ) | 6.57 ( 2.54 ) | 2.72 ( 1.27 ) |
| Extra dose administered | 3 | 6.06 ( 1.95 - 18.89 ) | 6.03 ( 12.57 ) | 6.02 ( 2.32 ) | 2.59 ( 1.14 ) |
| Conjunctivitis | 3 | 9.32 ( 2.99 - 29.08 ) | 9.27 ( 22.07 ) | 9.24 ( 3.57 ) | 3.21 ( 1.76 ) |
| Burning sensation | 2 | 6.73 ( 1.68 - 27.04 ) | 6.71 ( 9.69 ) | 6.69 ( 2.09 ) | 2.74 ( 1.07 ) |
| Stress | 2 | 10.86 ( 2.7 - 43.7 ) | 10.82 ( 17.76 ) | 10.78 ( 3.36 ) | 3.43 ( 1.75 ) |
| Injection site hypersensitivity | 2 | 111.99 ( 27.11 - 462.73 ) | 111.51 ( 209.92 ) | 106.91 ( 32.62 ) | 6.74 ( 5.02 ) |
| Ear infection | 2 | 4.34 ( 1.08 - 17.44 ) | 4.33 ( 5.12 ) | 4.32 ( 1.35 ) | 2.11 ( 0.44 ) |
| Maternal exposure during pregnancy | 2 | 13.66 ( 3.39 - 54.99 ) | 13.61 ( 23.24 ) | 13.54 ( 4.22 ) | 3.76 ( 2.08 ) |
| Influenza like illness | 2 | 10.1 ( 2.51 - 40.61 ) | 10.06 ( 16.26 ) | 10.02 ( 3.13 ) | 3.33 ( 1.65 ) |
| Sacroiliitis | 2 | 88.82 ( 21.63 - 364.76 ) | 88.44 ( 167.15 ) | 85.52 ( 26.23 ) | 6.42 ( 4.71 ) |
| Acne fulminans | 2 | 97.2 ( 23.62 - 400.05 ) | 96.78 ( 182.7 ) | 93.3 ( 28.56 ) | 6.54 ( 4.83 ) |
| Hidradenitis | 2 | 57.24 ( 14.06 - 233.1 ) | 56.99 ( 107.64 ) | 55.78 ( 17.23 ) | 5.8 ( 4.1 ) |
| Sapho syndrome | 2 | 139.24 ( 33.46 - 579.42 ) | 138.63 ( 259.28 ) | 131.58 ( 39.91 ) | 7.04 ( 5.31 ) |
| Swelling | 2 | 5.26 ( 1.31 - 21.12 ) | 5.24 ( 6.85 ) | 5.23 ( 1.63 ) | 2.39 ( 0.71 ) |
| Reaction to colouring | 2 | 343.46 ( 78.32 - 1506.2 ) | 341.96 ( 599.95 ) | 301.85 ( 87.62 ) | 8.24 ( 6.43 ) |
| Therapeutic response decreased | 2 | 3.67 ( 0.91 - 14.72 ) | 3.66 ( 3.86 ) | 3.65 ( 1.14 ) | 1.87 ( 0.2 ) |
| Fungal infection | 2 | 10.22 ( 2.54 - 41.09 ) | 10.18 ( 16.49 ) | 10.14 ( 3.16 ) | 3.34 ( 1.67 ) |
| Injection site haemorrhage | 2 | 4.02 ( 1 - 16.13 ) | 4 ( 4.51 ) | 4 ( 1.25 ) | 2 ( 0.33 ) |
| Streptococcal infection | 2 | 15.42 ( 3.83 - 62.1 ) | 15.36 ( 26.69 ) | 15.27 ( 4.76 ) | 3.93 ( 2.25 ) |

Abbreviation: ROR, reporting odds ratio; PRR, proportional reporting ratio; EBGM, empirical Bayesian geometric mean; EBGM05, the lower limit of the 95% CI of EBGM; IC, information component; IC025, the lower limit of the 95% CI of the IC; CI, confidence interval; PT, preferred term.
