# Supplementary material for: Real-world safety assessment of Ixekizumab based on the FDA Adverse Event Reporting System (FAERS)
Source: PLoS One. 2025 May 23;20(5):e0323973. doi: 10.1371/journal.pone.0323973 (PMC12101745; doi:10.1371/journal.pone.0323973)
Supplement: S7 Table — (DOCX) [file pone.0323973.s007.docx]

Supplementary Table 7:

Top 50 most frequent positive signal adverse events of Ixekizumab at the PT level in patients aged 18 to 65 from FAERS data

| PT | Case numbers | ROR(95%CI) | PRR(χ^2^) | EBGM(EBGM05) | IC(IC025) |
| --- | --- | --- | --- | --- | --- |
| Injection site pain | 1,050 | 7.71 ( 7.24 - 8.2 ) | 7.45 ( 5801.6 ) | 7.35 ( 6.98 ) | 2.88 ( 2.79 ) |
| Injection site erythema | 774 | 13.94 ( 12.97 - 14.99 ) | 13.58 ( 8778.31 ) | 13.22 ( 12.44 ) | 3.72 ( 3.62 ) |
| Psoriasis | 769 | 11.74 ( 10.92 - 12.62 ) | 11.44 ( 7168.36 ) | 11.19 ( 10.53 ) | 3.48 ( 3.38 ) |
| Drug ineffective | 640 | 1.23 ( 1.13 - 1.33 ) | 1.22 ( 26.28 ) | 1.22 ( 1.14 ) | 0.29 ( 0.17 ) |
| Injection site swelling | 629 | 16.5 ( 15.22 - 17.88 ) | 16.14 ( 8645.8 ) | 15.63 ( 14.61 ) | 3.97 ( 3.85 ) |
| Therapy interrupted | 533 | 14.06 ( 12.89 - 15.33 ) | 13.8 ( 6155.03 ) | 13.43 ( 12.49 ) | 3.75 ( 3.62 ) |
| Covid-19 | 496 | 3.58 ( 3.27 - 3.91 ) | 3.53 ( 896.59 ) | 3.51 ( 3.26 ) | 1.81 ( 1.68 ) |
| Injection site reaction | 490 | 15.37 ( 14.04 - 16.83 ) | 15.12 ( 6262.39 ) | 14.67 ( 13.6 ) | 3.87 ( 3.74 ) |
| Incorrect dose administered | 329 | 3.12 ( 2.8 - 3.48 ) | 3.1 ( 466.27 ) | 3.08 ( 2.82 ) | 1.63 ( 1.46 ) |
| Injection site pruritus | 316 | 9.17 ( 8.2 - 10.25 ) | 9.07 ( 2229.25 ) | 8.92 ( 8.12 ) | 3.16 ( 2.99 ) |
| Nasopharyngitis | 315 | 3.18 ( 2.84 - 3.55 ) | 3.15 ( 461.74 ) | 3.14 ( 2.86 ) | 1.65 ( 1.49 ) |
| Rash | 286 | 1.46 ( 1.3 - 1.64 ) | 1.46 ( 41.42 ) | 1.46 ( 1.32 ) | 0.54 ( 0.37 ) |
| Sinusitis | 284 | 4.85 ( 4.31 - 5.46 ) | 4.81 ( 850.57 ) | 4.77 ( 4.33 ) | 2.25 ( 2.08 ) |
| Arthralgia | 275 | 1.26 ( 1.12 - 1.42 ) | 1.26 ( 14.83 ) | 1.26 ( 1.14 ) | 0.33 ( 0.16 ) |
| Therapy cessation | 269 | 7.86 ( 6.96 - 8.87 ) | 7.79 ( 1567.22 ) | 7.68 ( 6.94 ) | 2.94 ( 2.76 ) |
| Product dose omission issue | 256 | 1.75 ( 1.54 - 1.98 ) | 1.74 ( 80.63 ) | 1.74 ( 1.57 ) | 0.8 ( 0.62 ) |
| Infection | 247 | 3.87 ( 3.41 - 4.39 ) | 3.84 ( 515.99 ) | 3.82 ( 3.44 ) | 1.93 ( 1.75 ) |
| Malaise | 236 | 1.23 ( 1.08 - 1.4 ) | 1.23 ( 10.08 ) | 1.23 ( 1.1 ) | 0.3 ( 0.11 ) |
| Pruritus | 233 | 1.28 ( 1.12 - 1.45 ) | 1.27 ( 13.84 ) | 1.27 ( 1.14 ) | 0.35 ( 0.16 ) |
| Injection site warmth | 227 | 23.24 ( 20.33 - 26.57 ) | 23.06 ( 4563.91 ) | 22.01 ( 19.68 ) | 4.46 ( 4.26 ) |
| Injection site urticaria | 219 | 16.32 ( 14.25 - 18.68 ) | 16.2 ( 3018.15 ) | 15.68 ( 14 ) | 3.97 ( 3.77 ) |
| Illness | 218 | 4.73 ( 4.14 - 5.41 ) | 4.7 ( 629.97 ) | 4.66 ( 4.17 ) | 2.22 ( 2.02 ) |
| Psoriatic arthropathy | 213 | 8.08 ( 7.06 - 9.26 ) | 8.03 ( 1289.54 ) | 7.91 ( 7.06 ) | 2.98 ( 2.78 ) |
| Inappropriate schedule of product administration | 206 | 1.99 ( 1.74 - 2.28 ) | 1.98 ( 100.39 ) | 1.98 ( 1.76 ) | 0.98 ( 0.78 ) |
| Injection site mass | 201 | 9.56 ( 8.31 - 11 ) | 9.5 ( 1498.93 ) | 9.33 ( 8.3 ) | 3.22 ( 3.02 ) |
| Injection site rash | 190 | 12.12 ( 10.49 - 14.01 ) | 12.05 ( 1876.93 ) | 11.77 ( 10.43 ) | 3.56 ( 3.34 ) |
| Urticaria | 188 | 2.17 ( 1.88 - 2.5 ) | 2.16 ( 116.6 ) | 2.15 ( 1.91 ) | 1.11 ( 0.89 ) |
| Influenza | 182 | 3.03 ( 2.62 - 3.5 ) | 3.01 ( 243.79 ) | 3 ( 2.65 ) | 1.59 ( 1.37 ) |
| Urinary tract infection | 181 | 2.6 ( 2.25 - 3.01 ) | 2.59 ( 176.33 ) | 2.58 ( 2.28 ) | 1.37 ( 1.15 ) |
| Injection site haemorrhage | 180 | 4.49 ( 3.87 - 5.2 ) | 4.46 ( 479.74 ) | 4.43 ( 3.92 ) | 2.15 ( 1.93 ) |
| Injection site bruising | 176 | 4.49 ( 3.87 - 5.21 ) | 4.47 ( 470.31 ) | 4.44 ( 3.92 ) | 2.15 ( 1.93 ) |
| Ear infection | 166 | 11.77 ( 10.09 - 13.74 ) | 11.71 ( 1585.84 ) | 11.44 ( 10.05 ) | 3.52 ( 3.29 ) |
| Pneumonia | 153 | 1.22 ( 1.04 - 1.43 ) | 1.22 ( 5.95 ) | 1.22 ( 1.07 ) | 0.28 ( 0.05 ) |
| Hypersensitivity | 146 | 1.73 ( 1.47 - 2.03 ) | 1.72 ( 44.28 ) | 1.72 ( 1.5 ) | 0.78 ( 0.54 ) |
| Cellulitis | 137 | 5.68 ( 4.8 - 6.73 ) | 5.66 ( 519.77 ) | 5.6 ( 4.87 ) | 2.49 ( 2.24 ) |
| Bronchitis | 113 | 3.33 ( 2.77 - 4.01 ) | 3.33 ( 182.64 ) | 3.31 ( 2.83 ) | 1.73 ( 1.45 ) |
| Upper respiratory tract infection | 111 | 4.49 ( 3.73 - 5.42 ) | 4.48 ( 297.32 ) | 4.45 ( 3.8 ) | 2.15 ( 1.88 ) |
| Oropharyngeal pain | 107 | 2.01 ( 1.66 - 2.43 ) | 2.01 ( 53.82 ) | 2 ( 1.71 ) | 1 ( 0.72 ) |
| Arthritis | 105 | 3.23 ( 2.67 - 3.92 ) | 3.23 ( 160.28 ) | 3.21 ( 2.73 ) | 1.68 ( 1.4 ) |
| Fungal infection | 97 | 6.35 ( 5.2 - 7.76 ) | 6.33 ( 429.9 ) | 6.26 ( 5.29 ) | 2.65 ( 2.35 ) |
| Swelling | 84 | 1.66 ( 1.34 - 2.06 ) | 1.66 ( 21.99 ) | 1.66 ( 1.39 ) | 0.73 ( 0.41 ) |
| Pharyngitis streptococcal | 78 | 12.27 ( 9.8 - 15.37 ) | 12.24 ( 784.46 ) | 11.95 ( 9.9 ) | 3.58 ( 3.25 ) |
| Myocardial infarction | 78 | 2.12 ( 1.7 - 2.65 ) | 2.12 ( 46.09 ) | 2.12 ( 1.76 ) | 1.08 ( 0.76 ) |
| Herpes zoster | 74 | 2.67 ( 2.12 - 3.35 ) | 2.66 ( 76.46 ) | 2.65 ( 2.19 ) | 1.41 ( 1.07 ) |
| Treatment failure | 73 | 1.94 ( 1.54 - 2.45 ) | 1.94 ( 33.15 ) | 1.94 ( 1.6 ) | 0.95 ( 0.62 ) |
| Candida infection | 71 | 7.63 ( 6.03 - 9.65 ) | 7.61 ( 401.24 ) | 7.5 ( 6.17 ) | 2.91 ( 2.56 ) |
| Intentional dose omission | 71 | 5.41 ( 4.28 - 6.84 ) | 5.4 ( 251.64 ) | 5.35 ( 4.4 ) | 2.42 ( 2.08 ) |
| Underdose | 70 | 2.1 ( 1.66 - 2.66 ) | 2.1 ( 40.3 ) | 2.1 ( 1.72 ) | 1.07 ( 0.72 ) |
| Therapeutic product effect incomplete | 70 | 1.67 ( 1.32 - 2.11 ) | 1.67 ( 18.73 ) | 1.67 ( 1.37 ) | 0.74 ( 0.39 ) |
| Injection site induration | 69 | 10.72 ( 8.44 - 13.61 ) | 10.69 ( 592.66 ) | 10.47 ( 8.57 ) | 3.39 ( 3.04 ) |

Abbreviation: ROR, reporting odds ratio; PRR, proportional reporting ratio; EBGM, empirical Bayesian geometric mean; EBGM05, the lower limit of the 95% CI of EBGM; IC, information component; IC025, the lower limit of the 95% CI of the IC; CI, confidence interval; PT, preferred term.
