# Supplementary material for: Real-world safety assessment of Ixekizumab based on the FDA Adverse Event Reporting System (FAERS)
Source: PLoS One. 2025 May 23;20(5):e0323973. doi: 10.1371/journal.pone.0323973 (PMC12101745; doi:10.1371/journal.pone.0323973)
Supplement: S8 Table — (DOCX) [file pone.0323973.s008.docx]

Supplementary Table 8:

Top 50 most frequent positive signal adverse events of Ixekizumab at the PT level in patients aged 65 to 85 from FAERS data

| PT | Case numbers | ROR(95%CI) | PRR(χ^2^) | EBGM(EBGM05) | IC(IC025) |
| --- | --- | --- | --- | --- | --- |
| Injection site pain | 186 | 9.41 ( 8.13 - 10.9 ) | 9.17 ( 1346.47 ) | 9.1 ( 8.05 ) | 3.19 ( 2.97 ) |
| Drug ineffective | 179 | 1.96 ( 1.69 - 2.28 ) | 1.94 ( 81.97 ) | 1.93 ( 1.71 ) | 0.95 ( 0.73 ) |
| Psoriasis | 153 | 20.18 ( 17.16 - 23.72 ) | 19.71 ( 2674.69 ) | 19.39 ( 16.94 ) | 4.28 ( 4.04 ) |
| Therapy interrupted | 112 | 10.77 ( 8.93 - 12.99 ) | 10.6 ( 965.91 ) | 10.51 ( 8.98 ) | 3.39 ( 3.12 ) |
| Injection site erythema | 95 | 16.5 ( 13.46 - 20.24 ) | 16.27 ( 1343.37 ) | 16.05 ( 13.53 ) | 4 ( 3.71 ) |
| Covid-19 | 92 | 2.98 ( 2.43 - 3.67 ) | 2.96 ( 119.36 ) | 2.95 ( 2.48 ) | 1.56 ( 1.26 ) |
| Urinary tract infection | 72 | 2.76 ( 2.18 - 3.48 ) | 2.74 ( 79.43 ) | 2.73 ( 2.25 ) | 1.45 ( 1.11 ) |
| Injection site swelling | 71 | 19.09 ( 15.08 - 24.17 ) | 18.89 ( 1183.91 ) | 18.6 ( 15.27 ) | 4.22 ( 3.87 ) |
| Pain | 69 | 1.38 ( 1.09 - 1.75 ) | 1.38 ( 7.12 ) | 1.37 ( 1.13 ) | 0.46 ( 0.11 ) |
| Pneumonia | 67 | 1.33 ( 1.05 - 1.69 ) | 1.33 ( 5.41 ) | 1.33 ( 1.08 ) | 0.41 ( 0.05 ) |
| Arthralgia | 65 | 1.54 ( 1.2 - 1.96 ) | 1.53 ( 12.01 ) | 1.53 ( 1.25 ) | 0.61 ( 0.26 ) |
| Incorrect dose administered | 63 | 3.01 ( 2.35 - 3.86 ) | 2.99 ( 83.41 ) | 2.98 ( 2.42 ) | 1.58 ( 1.21 ) |
| Pruritus | 61 | 1.68 ( 1.31 - 2.16 ) | 1.67 ( 16.64 ) | 1.67 ( 1.35 ) | 0.74 ( 0.37 ) |
| Product dose omission issue | 61 | 2.39 ( 1.85 - 3.07 ) | 2.37 ( 48.62 ) | 2.37 ( 1.92 ) | 1.25 ( 0.88 ) |
| Rheumatoid arthritis | 57 | 5.47 ( 4.21 - 7.11 ) | 5.43 ( 205.42 ) | 5.41 ( 4.35 ) | 2.44 ( 2.05 ) |
| Injection site reaction | 57 | 23.67 ( 18.19 - 30.8 ) | 23.46 ( 1201.33 ) | 23.01 ( 18.45 ) | 4.52 ( 4.14 ) |
| Infection | 55 | 3.77 ( 2.89 - 4.92 ) | 3.75 ( 110.64 ) | 3.74 ( 2.99 ) | 1.9 ( 1.51 ) |
| Drug intolerance | 53 | 5.29 ( 4.03 - 6.93 ) | 5.25 ( 181.77 ) | 5.23 ( 4.17 ) | 2.39 ( 1.99 ) |
| Condition aggravated | 50 | 1.65 ( 1.25 - 2.17 ) | 1.64 ( 12.55 ) | 1.64 ( 1.3 ) | 0.71 ( 0.31 ) |
| Drug hypersensitivity | 49 | 1.93 ( 1.46 - 2.56 ) | 1.92 ( 21.81 ) | 1.92 ( 1.52 ) | 0.94 ( 0.53 ) |
| Therapeutic product effect decreased | 49 | 13.82 ( 10.41 - 18.33 ) | 13.72 ( 571.11 ) | 13.56 ( 10.71 ) | 3.76 ( 3.35 ) |
| Nasopharyngitis | 46 | 2.52 ( 1.88 - 3.37 ) | 2.51 ( 41.71 ) | 2.5 ( 1.96 ) | 1.32 ( 0.9 ) |
| Sinusitis | 46 | 5.15 ( 3.85 - 6.89 ) | 5.12 ( 152.17 ) | 5.1 ( 4 ) | 2.35 ( 1.93 ) |
| Therapy cessation | 46 | 6.73 ( 5.03 - 9 ) | 6.68 ( 221.3 ) | 6.65 ( 5.21 ) | 2.73 ( 2.31 ) |
| Cellulitis | 44 | 7.09 ( 5.27 - 9.55 ) | 7.05 ( 227.26 ) | 7.01 ( 5.47 ) | 2.81 ( 2.38 ) |
| Inappropriate schedule of product administration | 43 | 2.27 ( 1.68 - 3.06 ) | 2.26 ( 30.23 ) | 2.26 ( 1.76 ) | 1.17 ( 0.74 ) |
| Psoriatic arthropathy | 39 | 15.84 ( 11.53 - 21.74 ) | 15.74 ( 531.27 ) | 15.54 ( 11.92 ) | 3.96 ( 3.5 ) |
| Injection site pruritus | 38 | 12.45 ( 9.04 - 17.16 ) | 12.38 ( 393.45 ) | 12.26 ( 9.37 ) | 3.62 ( 3.15 ) |
| Injection site haemorrhage | 38 | 5.5 ( 4 - 7.57 ) | 5.47 ( 138.44 ) | 5.45 ( 4.17 ) | 2.45 ( 1.98 ) |
| Musculoskeletal stiffness | 35 | 4.11 ( 2.94 - 5.73 ) | 4.09 ( 81.45 ) | 4.08 ( 3.09 ) | 2.03 ( 1.54 ) |
| Illness | 30 | 2.59 ( 1.81 - 3.7 ) | 2.58 ( 28.97 ) | 2.57 ( 1.91 ) | 1.36 ( 0.84 ) |
| Therapeutic product effect incomplete | 29 | 3.2 ( 2.22 - 4.61 ) | 3.19 ( 43.46 ) | 3.18 ( 2.34 ) | 1.67 ( 1.14 ) |
| Chest pain | 29 | 1.68 ( 1.16 - 2.41 ) | 1.67 ( 7.86 ) | 1.67 ( 1.23 ) | 0.74 ( 0.21 ) |
| Injection site mass | 27 | 8.37 ( 5.72 - 12.23 ) | 8.34 ( 173.09 ) | 8.28 ( 6.03 ) | 3.05 ( 2.5 ) |
| Injection site warmth | 27 | 39.69 ( 27.02 - 58.3 ) | 39.52 ( 979.52 ) | 38.22 ( 27.7 ) | 5.26 ( 4.7 ) |
| Injection site bruising | 27 | 4.69 ( 3.21 - 6.85 ) | 4.67 ( 77.66 ) | 4.66 ( 3.39 ) | 2.22 ( 1.67 ) |
| Bronchitis | 27 | 2.87 ( 1.96 - 4.18 ) | 2.86 ( 32.56 ) | 2.85 ( 2.08 ) | 1.51 ( 0.96 ) |
| Urticaria | 27 | 2.31 ( 1.58 - 3.37 ) | 2.31 ( 19.95 ) | 2.3 ( 1.68 ) | 1.2 ( 0.66 ) |
| Chest discomfort | 27 | 2.63 ( 1.8 - 3.84 ) | 2.62 ( 27.06 ) | 2.62 ( 1.91 ) | 1.39 ( 0.84 ) |
| Joint swelling | 26 | 1.91 ( 1.3 - 2.81 ) | 1.91 ( 11.27 ) | 1.91 ( 1.38 ) | 0.93 ( 0.38 ) |
| Fungal infection | 26 | 8.79 ( 5.97 - 12.94 ) | 8.76 ( 177.38 ) | 8.7 ( 6.29 ) | 3.12 ( 2.56 ) |
| Hyperhidrosis | 26 | 2.34 ( 1.59 - 3.44 ) | 2.33 ( 19.76 ) | 2.33 ( 1.69 ) | 1.22 ( 0.66 ) |
| Herpes zoster | 25 | 3.29 ( 2.22 - 4.88 ) | 3.28 ( 39.62 ) | 3.28 ( 2.36 ) | 1.71 ( 1.14 ) |
| Treatment failure | 25 | 5.41 ( 3.65 - 8.02 ) | 5.39 ( 89.12 ) | 5.37 ( 3.86 ) | 2.43 ( 1.86 ) |
| Injection site rash | 24 | 16.38 ( 10.94 - 24.53 ) | 16.32 ( 340.41 ) | 16.11 ( 11.49 ) | 4.01 ( 3.43 ) |
| Bowel movement irregularity | 24 | 22.44 ( 14.97 - 33.63 ) | 22.36 ( 480.16 ) | 21.94 ( 15.64 ) | 4.46 ( 3.87 ) |
| Flushing | 24 | 4.18 ( 2.8 - 6.25 ) | 4.17 ( 57.63 ) | 4.16 ( 2.97 ) | 2.06 ( 1.48 ) |
| Skin exfoliation | 23 | 3.76 ( 2.5 - 5.67 ) | 3.75 ( 46.34 ) | 3.74 ( 2.66 ) | 1.9 ( 1.31 ) |
| Therapy non-responder | 23 | 5.7 ( 3.78 - 8.59 ) | 5.68 ( 88.35 ) | 5.66 ( 4.01 ) | 2.5 ( 1.91 ) |
| Lymphadenopathy | 22 | 7.47 ( 4.91 - 11.37 ) | 7.45 ( 122.12 ) | 7.41 ( 5.21 ) | 2.89 ( 2.28 ) |

Abbreviation: ROR, reporting odds ratio; PRR, proportional reporting ratio; EBGM, empirical Bayesian geometric mean; EBGM05, the lower limit of the 95% CI of EBGM; IC, information component; IC025, the lower limit of the 95% CI of the IC; CI, confidence interval; PT, preferred term.
