# Supplementary material for: Real-world safety assessment of Ixekizumab based on the FDA Adverse Event Reporting System (FAERS)
Source: PLoS One. 2025 May 23;20(5):e0323973. doi: 10.1371/journal.pone.0323973 (PMC12101745; doi:10.1371/journal.pone.0323973)
Supplement: S9 Table — (DOCX) [file pone.0323973.s009.docx]

Supplementary Table 9:

Top 50 most frequent positive signal adverse events of Ixekizumab excluding common medication co-usage at the PT level from FAERS data

| PT | Case numbers | ROR(95%CI) | PRR(χ^2^) | EBGM(EBGM05) | IC(IC025) |
| --- | --- | --- | --- | --- | --- |
| Injection site pain | 3,422 | 16.92 ( 16.33 - 17.52 ) | 15.82 ( 46687.16 ) | 15.5 ( 15.05 ) | 3.95 ( 3.9 ) |
| Psoriasis | 2,025 | 18.22 ( 17.42 - 19.06 ) | 17.52 ( 30865.86 ) | 17.13 ( 16.49 ) | 4.1 ( 4.03 ) |
| Injection site erythema | 1,912 | 27.35 ( 26.1 - 28.65 ) | 26.33 ( 45012.26 ) | 25.43 ( 24.46 ) | 4.67 ( 4.6 ) |
| Drug ineffective | 1,900 | 1.63 ( 1.56 - 1.71 ) | 1.61 ( 448.33 ) | 1.61 ( 1.55 ) | 0.69 ( 0.62 ) |
| Injection site swelling | 1,434 | 29.34 ( 27.82 - 30.96 ) | 28.52 ( 36670.5 ) | 27.47 ( 26.27 ) | 4.78 ( 4.7 ) |
| Injection site reaction | 1,408 | 32.28 ( 30.58 - 34.07 ) | 31.39 ( 39728.87 ) | 30.12 ( 28.78 ) | 4.91 ( 4.83 ) |
| Incorrect dose administered | 759 | 4.13 ( 3.84 - 4.44 ) | 4.08 ( 1763.96 ) | 4.07 ( 3.83 ) | 2.02 ( 1.92 ) |
| Covid-19 | 749 | 3.46 ( 3.22 - 3.72 ) | 3.42 ( 1285.05 ) | 3.41 ( 3.21 ) | 1.77 ( 1.66 ) |
| Product dose omission issue | 686 | 2.53 ( 2.35 - 2.73 ) | 2.51 ( 625.41 ) | 2.51 ( 2.35 ) | 1.33 ( 1.21 ) |
| Therapy interrupted | 682 | 10.46 ( 9.69 - 11.29 ) | 10.33 ( 5672.85 ) | 10.2 ( 9.57 ) | 3.35 ( 3.24 ) |
| Injection site pruritus | 639 | 14.63 ( 13.52 - 15.83 ) | 14.46 ( 7854.24 ) | 14.19 ( 13.29 ) | 3.83 ( 3.71 ) |
| Injection site urticaria | 577 | 33.97 ( 31.23 - 36.94 ) | 33.58 ( 17432.66 ) | 32.13 ( 29.95 ) | 5.01 ( 4.88 ) |
| Rash | 550 | 1.55 ( 1.42 - 1.68 ) | 1.54 ( 104.92 ) | 1.54 ( 1.43 ) | 0.62 ( 0.5 ) |
| Injection site mass | 522 | 15.86 ( 14.54 - 17.31 ) | 15.71 ( 7039 ) | 15.39 ( 14.31 ) | 3.94 ( 3.82 ) |
| Injection site haemorrhage | 490 | 8.2 ( 7.49 - 8.96 ) | 8.13 ( 3031.17 ) | 8.05 ( 7.47 ) | 3.01 ( 2.88 ) |
| Pruritus | 471 | 1.58 ( 1.44 - 1.73 ) | 1.57 ( 98.36 ) | 1.57 ( 1.46 ) | 0.65 ( 0.52 ) |
| Arthralgia | 467 | 1.36 ( 1.24 - 1.49 ) | 1.36 ( 43.85 ) | 1.36 ( 1.26 ) | 0.44 ( 0.3 ) |
| Nasopharyngitis | 463 | 3.01 ( 2.75 - 3.3 ) | 2.99 ( 613.29 ) | 2.98 ( 2.76 ) | 1.58 ( 1.44 ) |
| Injection site rash | 441 | 21.15 ( 19.23 - 23.26 ) | 20.97 ( 8152.5 ) | 20.4 ( 18.84 ) | 4.35 ( 4.21 ) |
| Inappropriate schedule of product administration | 427 | 2.29 ( 2.08 - 2.52 ) | 2.28 ( 305.98 ) | 2.27 ( 2.1 ) | 1.18 ( 1.04 ) |
| Injection site warmth | 412 | 38.5 ( 34.85 - 42.52 ) | 38.18 ( 14168.51 ) | 36.31 ( 33.41 ) | 5.18 ( 5.04 ) |
| Urticaria | 399 | 3.14 ( 2.84 - 3.46 ) | 3.12 ( 573.59 ) | 3.11 ( 2.86 ) | 1.64 ( 1.49 ) |
| Injection site bruising | 397 | 7.01 ( 6.35 - 7.74 ) | 6.96 ( 2009.97 ) | 6.91 ( 6.35 ) | 2.79 ( 2.64 ) |
| Sinusitis | 385 | 4.63 ( 4.18 - 5.12 ) | 4.6 ( 1079.48 ) | 4.58 ( 4.21 ) | 2.19 ( 2.05 ) |
| Therapy cessation | 361 | 6.5 ( 5.86 - 7.21 ) | 6.46 ( 1653.63 ) | 6.41 ( 5.88 ) | 2.68 ( 2.53 ) |
| Infection | 357 | 2.97 ( 2.67 - 3.29 ) | 2.95 ( 459.8 ) | 2.94 ( 2.7 ) | 1.56 ( 1.4 ) |
| Hypersensitivity | 308 | 1.99 ( 1.78 - 2.23 ) | 1.98 ( 150.51 ) | 1.98 ( 1.8 ) | 0.99 ( 0.82 ) |
| Urinary tract infection | 307 | 2.19 ( 1.96 - 2.45 ) | 2.18 ( 196.87 ) | 2.18 ( 1.98 ) | 1.12 ( 0.96 ) |
| Illness | 304 | 3.13 ( 2.79 - 3.5 ) | 3.11 ( 435.39 ) | 3.11 ( 2.83 ) | 1.63 ( 1.47 ) |
| Psoriatic arthropathy | 281 | 7.66 ( 6.81 - 8.61 ) | 7.62 ( 1599.96 ) | 7.55 ( 6.84 ) | 2.92 ( 2.74 ) |
| Influenza | 276 | 2.92 ( 2.59 - 3.28 ) | 2.91 ( 344.3 ) | 2.9 ( 2.62 ) | 1.54 ( 1.36 ) |
| Underdose | 232 | 3.34 ( 2.94 - 3.81 ) | 3.33 ( 377.65 ) | 3.32 ( 2.98 ) | 1.73 ( 1.54 ) |
| Cellulitis | 210 | 5.37 ( 4.69 - 6.15 ) | 5.35 ( 738.43 ) | 5.32 ( 4.75 ) | 2.41 ( 2.21 ) |
| Ear infection | 207 | 9.25 ( 8.06 - 10.62 ) | 9.22 ( 1498.07 ) | 9.11 ( 8.12 ) | 3.19 ( 2.99 ) |
| Accidental underdose | 189 | 14.02 ( 12.14 - 16.19 ) | 13.97 ( 2232.75 ) | 13.72 ( 12.16 ) | 3.78 ( 3.57 ) |
| Upper respiratory tract infection | 178 | 4.77 ( 4.12 - 5.53 ) | 4.76 ( 525.46 ) | 4.73 ( 4.18 ) | 2.24 ( 2.03 ) |
| Bronchitis | 167 | 2.9 ( 2.49 - 3.38 ) | 2.9 ( 206.64 ) | 2.89 ( 2.54 ) | 1.53 ( 1.31 ) |
| Oropharyngeal pain | 167 | 2.17 ( 1.87 - 2.53 ) | 2.17 ( 105.16 ) | 2.17 ( 1.91 ) | 1.12 ( 0.89 ) |
| Fungal infection | 161 | 6.01 ( 5.14 - 7.02 ) | 5.99 ( 664.17 ) | 5.95 ( 5.22 ) | 2.57 ( 2.34 ) |
| Injection site induration | 150 | 18.27 ( 15.54 - 21.49 ) | 18.22 ( 2381.55 ) | 17.8 ( 15.54 ) | 4.15 ( 3.92 ) |
| Therapy non-responder | 148 | 3.23 ( 2.74 - 3.79 ) | 3.22 ( 225.51 ) | 3.21 ( 2.8 ) | 1.68 ( 1.44 ) |
| Arthritis | 141 | 2.21 ( 1.87 - 2.61 ) | 2.21 ( 93 ) | 2.2 ( 1.92 ) | 1.14 ( 0.9 ) |
| Herpes zoster | 131 | 2.71 ( 2.28 - 3.21 ) | 2.7 ( 140.01 ) | 2.7 ( 2.33 ) | 1.43 ( 1.18 ) |
| Oral candidiasis | 123 | 13.04 ( 10.91 - 15.59 ) | 13.01 ( 1339.88 ) | 12.8 ( 11.02 ) | 3.68 ( 3.42 ) |
| Candida infection | 114 | 7.25 ( 6.03 - 8.73 ) | 7.24 ( 607.16 ) | 7.18 ( 6.15 ) | 2.84 ( 2.57 ) |
| Injection site discomfort | 112 | 13.39 ( 11.1 - 16.15 ) | 13.36 ( 1257.72 ) | 13.14 ( 11.23 ) | 3.72 ( 3.44 ) |
| Influenza like illness | 110 | 1.97 ( 1.63 - 2.37 ) | 1.96 ( 52.05 ) | 1.96 ( 1.68 ) | 0.97 ( 0.7 ) |
| Maternal exposure during pregnancy | 110 | 1.29 ( 1.07 - 1.55 ) | 1.29 ( 7.12 ) | 1.29 ( 1.1 ) | 0.37 ( 0.09 ) |
| Swelling | 109 | 1.35 ( 1.12 - 1.63 ) | 1.35 ( 10.07 ) | 1.35 ( 1.16 ) | 0.44 ( 0.16 ) |
| Pharyngitis streptococcal | 108 | 12.26 ( 10.13 - 14.83 ) | 12.23 ( 1095.45 ) | 12.04 ( 10.27 ) | 3.59 ( 3.31 ) |

Abbreviation: ROR, reporting odds ratio; PRR, proportional reporting ratio; EBGM, empirical Bayesian geometric mean; EBGM05, the lower limit of the 95% CI of EBGM; IC, information component; IC025, the lower limit of the 95% CI of the IC; CI, confidence interval; PT, preferred term.
